# AAV gene therapy for hereditary spastic paraplegia type 50: a phase 1 trial in a single patient

**DOI:** 10.1038/s41591-024-03078-4

**Published:** 2024-06-28

**Authors:** James J. Dowling, Terry Pirovolakis, Keshini Devakandan, Ana Stosic, Mia Pidsadny, Elisa Nigro, Mustafa Sahin, Darius Ebrahimi-Fakhari, Souad Messahel, Ganapathy Varadarajan, Benjamin M. Greenberg, Xin Chen, Berge A. Minassian, Ronald Cohn, Carsten G. Bonnemann, Steven J. Gray

**Affiliations:** 1 Precision Child Health, Hospital for Sick Children, Toronto, Ontario Canada; 2 Division of Neurology and Program for Genetics and Genome Biology, Hospital for Sick Children, Toronto, Ontario Canada; 3 Departments of Paediatrics and Molecular Genetics, University of Toronto, Toronto, Ontario Canada; 4 CureSPG50 Foundation, Toronto, Ontario Canada; 5 Department of Neurology, Boston Children’s Hospital, Boston, MA USA; 6 Department of Pediatrics, University of Texas Southwestern Medical Center, Dallas, TX USA; 7 Neuromuscular & Neurogenetic Diseases of Childhood, Neurogenetics Branch (NGB), Bethesda, MD USA

**Keywords:** Paediatric neurological disorders, Drug safety, Drug development

## Abstract

There are more than 10,000 individual rare diseases and most are without therapy. Personalized genetic therapy represents one promising approach for their treatment. We present a road map for individualized treatment of an ultra-rare disease by establishing a gene replacement therapy developed for a single patient with hereditary spastic paraplegia type 50 (SPG50). Through a multicenter collaboration, an adeno-associated virus-based gene therapy product carrying the *AP4M1* gene was created and successfully administered intrathecally to a 4-year-old patient within 3 years of diagnosis as part of a single-patient phase 1 trial. Primary endpoints were safety and tolerability, and secondary endpoints evaluated efficacy. At 12 months after dosing, the therapy was well tolerated. No serious adverse events were observed, with minor events, including transient neutropenia and *Clostridioides difficile* gastroenteritis, experienced but resolved. Preliminary efficacy measures suggest a stabilization of the disease course. Longer follow-up is needed to confirm the safety and provide additional insights on the efficacy of the therapy. Overall, this report supports the safety of gene therapy for SPG50 and provides insights into precision therapy development for rare diseases. Clinical trial registration: NCT06069687.

## Abstract (web summary)

In a phase 1 trial in a single patient with hereditary spastic paraplegia type 50, a personalized AAV9-based gene therapy was developed within 3 years from diagnosis and was well tolerated, showing preliminary evidence of disease stabilization.

## Main

Rare diseases affect more than 400 million persons. They are associated with considerable disabilities, early mortality and disproportionate impacts on the healthcare system. Less than 5% have treatments, highlighting a critical need for new therapies. There is now the conceptual ability to develop gene- and/or mutation-specific treatments for many rare diseases^1,2^. However, important barriers exist, particularly related to patient numbers, development costs and lack of financial incentives.

Hereditary spastic paraplegia type 50 (SPG50) is a prototypical ultra-rare (affecting <1 in 50,000) disease, with fewer than 100 affected individuals identified^3,4^. It is caused by biallelic pathogenic variants in the *AP4M1* gene, encoding a subunit of the adaptor protein complex 4 (AP-4)^5–9^. Symptom onset is typically in infancy and includes global developmental delay, progressive microcephaly and abnormalities on brain magnetic resonance imaging (MRI)^3,4,10^. The disease is progressive, with loss of motor skills due to worsening spasticity, and is associated with serious morbidities^3,11^. By the second decade of life, most affected individuals are wheelchair dependent and manifest severe cognitive dysfunction. Lifespan is not fully established, but the disorder is considered life-limiting.

SPG50 is an ideal candidate disease for gene therapy. The coding sequence is small (1,359 base pairs) and fits within a self-complementary adeno-associated virus (scAAV) vector. Causative mutations result in loss of expression/function, so gene re-expression is anticipated to be effective, and the nature of the AP-4 complex as an obligate heterotetramer may protect against overexpression-related toxicity^12^. There is a relatively large therapeutic window, as disease progression occurs over years, with potential for functional benefit likely before irreversible disability. However, the disorder’s rarity precludes typical drug development pathways.

**Fig. 1:**
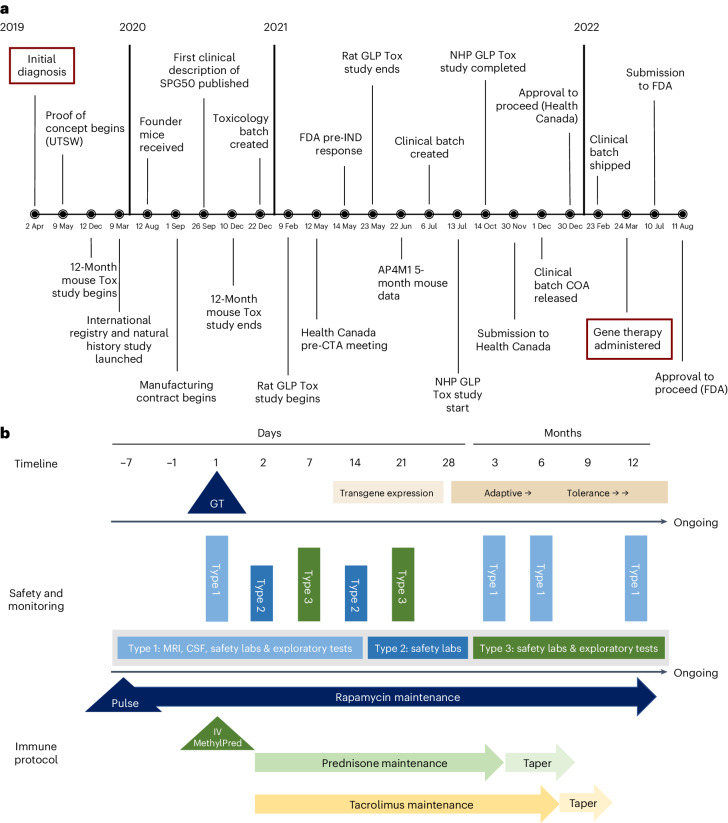
Development and implementation of individual gene therapy for SPG50. **a**, Timeline of the development of SPG50 gene therapy, from patient diagnosis through patient dosing, with key milestones highlighted. Note that the entire process, from diagnosis to dosing, took approximately 2.5 years. UTSW, University of Texas Southwestern; FDA, Food and Drug Administration; IND, investigational new drug; GLP, Good Laboratory Practice; NHP, nonhuman primate; Tox, toxicology; CTA, clinical trial application; COA, certificate of analysis. **b**, Outline of the single-patient clinical trial. The schematic depicts the postdosing safety and efficacy monitoring time points, along with the immunosuppression protocol. The comprehensive immunosuppression program was implemented to attempt to minimize the innate and adaptive immune responses and to promote tolerance to the gene therapy product. ‘GT’ indicates the gene therapy dosing. MRI of the brain and spine (with and without contrast) was done at baseline and at 3, 6 and 12 months after dosing. CSF analysis included cell count, protein concentration, oligoclonal bands and cytokine analysis. Exploratory tests included measurement of the AAV9 neutralizing antibody titer, serum cytokine analysis and ELISpot assay. Safety laboratory tests (‘safety labs’) included complete blood count with differential, erythrocyte sedimentation rate, C-reactive protein, liver function tests (alanine aminotransferase, aspartate aminotransferase, γ-glutamyl transferase, alkaline phosphatase), blood urea nitrogen/creatinine, urinalysis, electrocardiography and cardiac safety panel (troponin, pro-B-type natriuretic peptide, creatine kinase isotype MB). IV MethylPred, intravenous methylprednisolone.

We present a case wherein gene therapy was developed for a single male patient with SPG50 (Fig. 1a). The disease was diagnosed at age 18 months by whole-exome sequencing (*AP4M1* c.916 C>T, p.R306X; c.696dupG, p.E232GfsX21) based on a presentation of developmental delay (unable to stand or walk independently, no word production) and microcephaly. At diagnosis, based on our international registry (NCT04712812), the proband was the only Canadian individual with SPG50. Shortly after diagnosis, the family created the CureSPG50 Foundation with the goal of developing SPG50 gene therapy. At the predosing baseline, the patient could crawl 5 feet, pull himself up to stand momentarily at a table and walk a few steps with assistance. He had a pincer grasp and could feed himself with his hands, stack two blocks and scribble. He was nonverbal and had limited communication with gestures and nonword sounds. Physical examination was most notable for diffuse spasticity (lower extremity more affected than upper extremity) and hyperreflexia.

**Extended Data Fig. 1:**
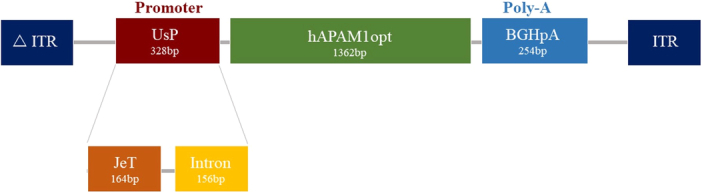
Schematic of the investigational product. Human, codon optimized AP4M1 (hAP4M1opt) with a bGH poly A tail was encapsulated into self-complementary (sc) AAV9. AP4M1 expression was governed by a ubiquitous promoter (UsP = JeT promoter with intron). See Chen et al., 2023, Journal of Clinical Investigation.

The investigational product was designed based on similar vectors made for CLN7 disease and giant axonal neuropathy^13^ and includes codon-optimized human *AP4M1* driven from the JeT promoter and encapsulated into scAAV9 (AAV9-*AP4M1*; Extended Data Fig. 1)^14^. Based on preclinical data^14^, a safety, toxicity and efficacy package for AAV9-*AP4M1* was filed to Health Canada, along with a clinical protocol and information on chemistry, manufacturing and control. A ‘no objection letter’ was received in December 2021, 2 years and 8 months after diagnosis. The study protocol enumerated the eligibility criteria and safety assessments based on the gene therapy trial for giant axonal neuropathy (NCT02362438)^15^, and efficacy measures were derived from the ongoing SPG50 natural history study (NCT04712812). Institutional ethics board approval was obtained in February 2022. Although the trial was not registered with ClinicalTrials.gov until October 2023, all inclusion and exclusion criteria, safety studies and outcome measures were established before study initiation and patient enrollment.

A single-patient trial (NCT06069687) was initiated (Fig. 1b), with dosing in March 2022, 2 years and 11 months after diagnosis. The primary outcome was safety, and secondary efficacy measures were related to spasticity. AAV9-*AP4M1* was administered at 1 × 10^15^ vector genomes (vg) through intrathecal delivery. This is among the largest doses of AAV9-based gene therapy ever administered into the cerebrospinal fluid (CSF).

**Extended Data Fig. 2:**
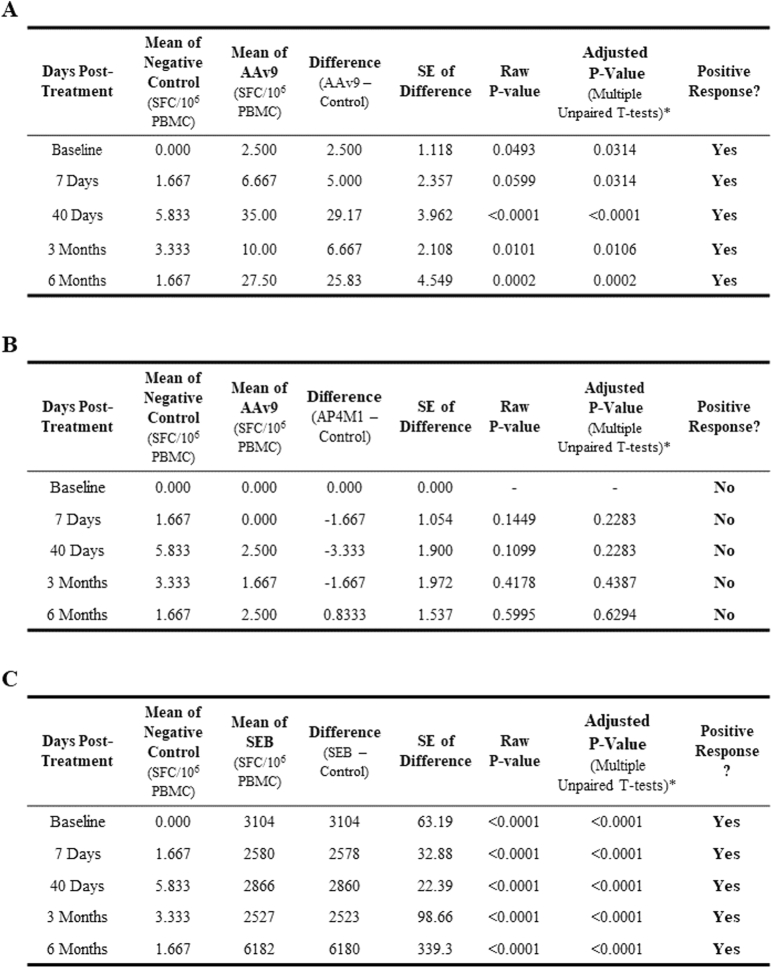
ELISpot assay reveals lack of immune response to AP4M1. (**a**) ELISpot to show IFN-y T-cell Responses toward AAV9. As expected, there is clear evidence of an immune response against AAV9. (**b**) ELISpot to show IFN-y T-cell Responses toward AP4M1. No significant response against AP4M1 was identified. (**c**) Positive control ELISpot used to confirm that the success of the assay. Multiple two-tailed t-tests were conducted to assess for significant differences between treatment responses and negative controls. *P-values were adjusted for multiple comparisons using the two-stage linear step-up method of Benjamini, Krieger, and Yekutieli (FDR = 5%).

We used an extensive immunosuppression protocol (prednisolone, sirolimus and tacrolimus) designed to reduce adverse immune responses and promote tolerance to the AP4M1 protein, given the patient’s predicted lack of endogenous expression. Based on enzyme-linked immunospot (ELISpot) data, the patient has not developed any appreciable anti-AP4M1 response (Extended Data Fig. 2).

**Fig. 2:**
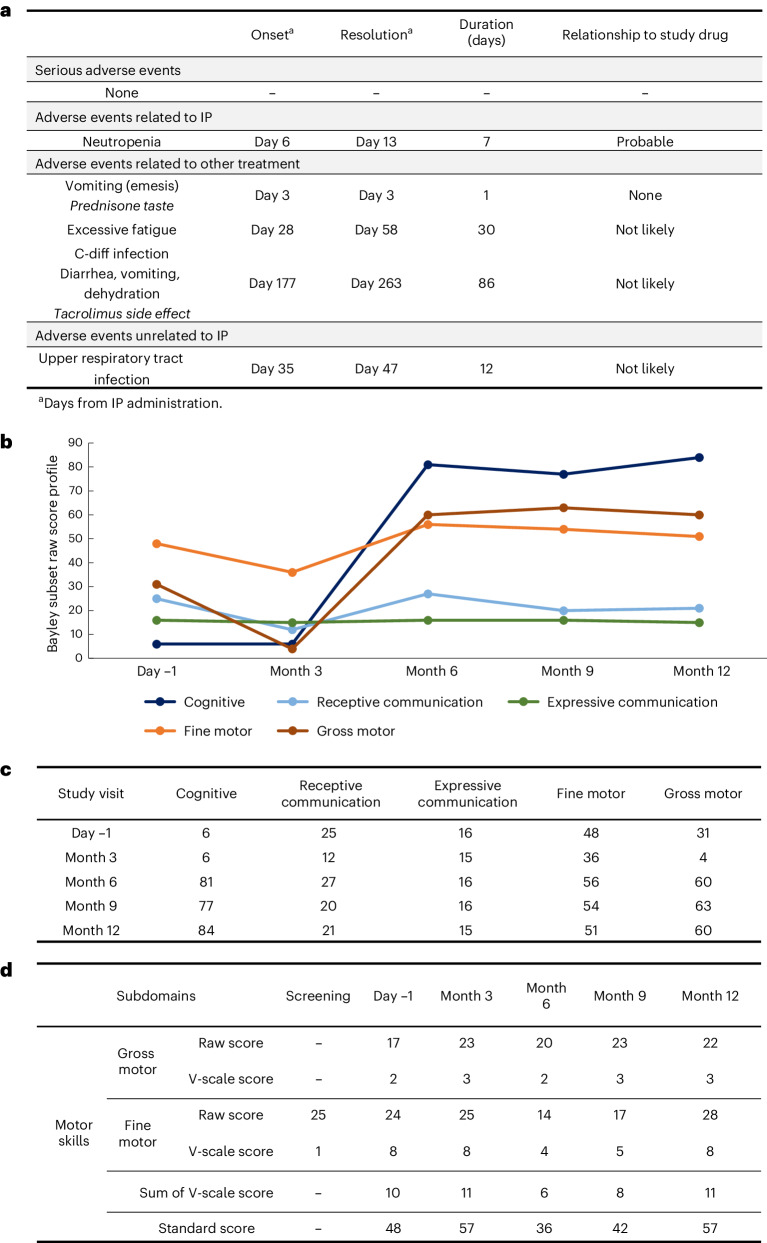
Safety and efficacy (Bayley Scale of Infant Development) in the SPG50 single-patient therapy trial. **a**, Enumeration of the adverse events reported in the clinical trial over the 1 year after dosing (IP, investigational product). No serious adverse events were observed. The patient experienced transient, asymptomatic neutropenia noted at 6 days after dosing. This resolved without intervention by day 13 after dosing. There was a prolonged episode of abdominal symptoms that included emesis, diarrhea, vomiting and abdominal pain. This episode prompted extensive evaluation, with the ultimate conclusion that the symptoms were due to side effects of tacrolimus plus *C. difficile* (C-diff) infection. **b**, Graphical representation of the longitudinal results of the Bayley Scale of Infant and Toddler Development, fourth edition. From 6 months after dosing, there were consistent increases in scores for all domains except expressive communication. This mirrors what was qualitatively observed by both the family and the examination team. **c**, Presentation of the longitudinal raw data from the Bayley scale (visualized graphically in **b**). Of note, the baseline and 3-month studies were complicated by challenges with the patient’s tolerance of the test. **d**, Scores from the motor skills submodule of the Vineland Adaptive Behavior Scale. Improvements were noted in both fine and gross motor performance.

**Extended Data Fig. 3:**
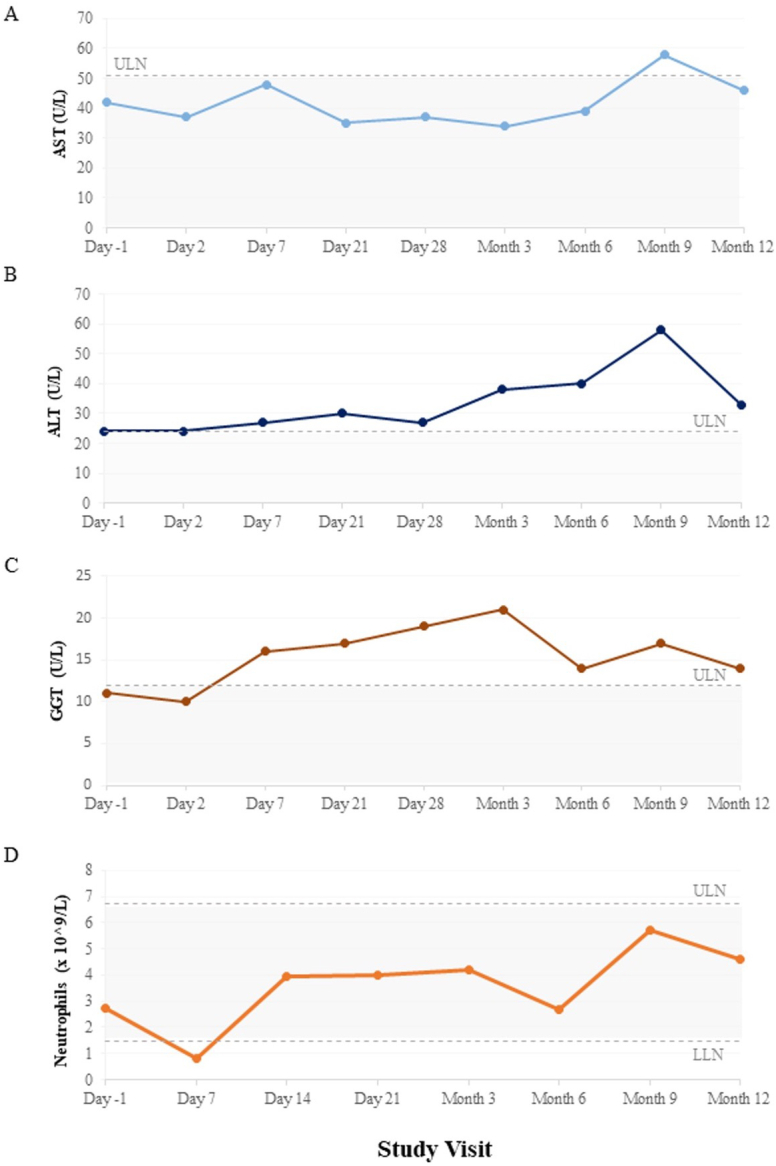
Safety lab trends during the 12 months post dosing. (**a**-**d**) Presented are the main safety laboratory studies, AST (**a**), ALT (**b**), GGT (**c**), and neutrophils (**d**), from baseline to 12 months post- dosing. Normative values for age are highlighted in gray. There was a single instance of neutropenia at day 7 post- dosing (0.8×109/L). ALT and GGT values were consistently outside of the normal range, but never reached a clinically meaningful increased level, and remained < 2-fold above normal limits.

**Extended Data Fig. 4:**
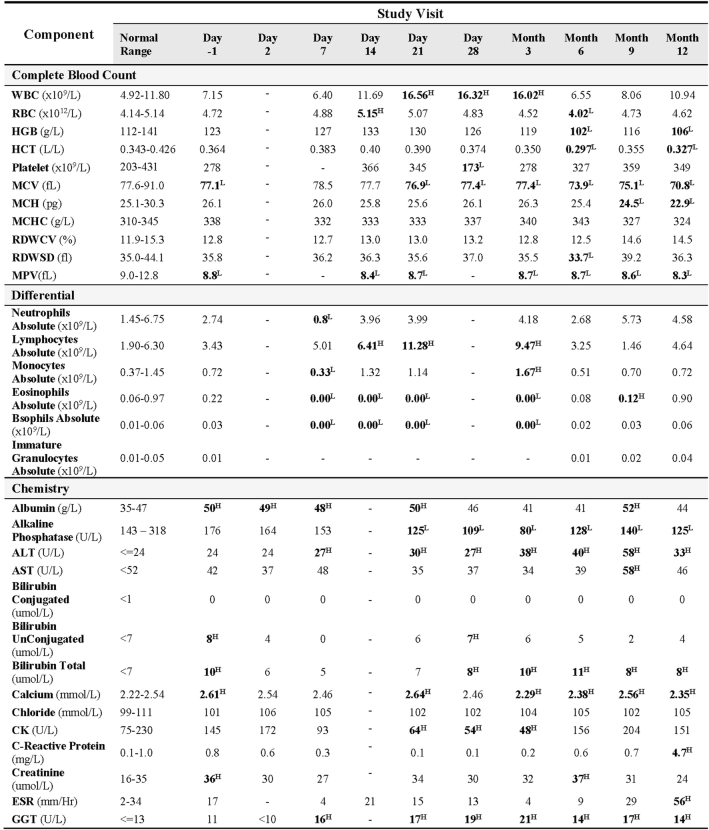
Listing of laboratory studies performed during the study. Laboratory values obtained are listed from baseline through the first 12 months of the study. Abnormal values (i.e. values outside the normal range) are highlighted in bold. Normative values, when available, are listed in the left most column.

**Extended Data Fig. 5:**
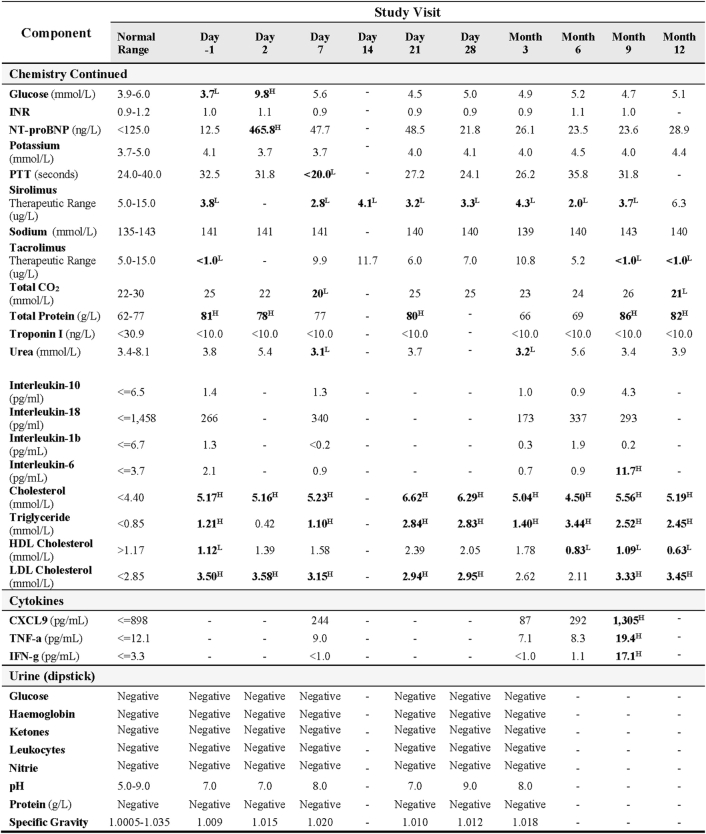
Listing of laboratory studies performed during the study (continued). Laboratory values obtained are listed from baseline through the first 12 months of the study. Abnormal values (i.e. values outside the normal range) are highlighted in bold. Normative values, when available, are listed in the left most column.

**Extended Data Fig. 6:**
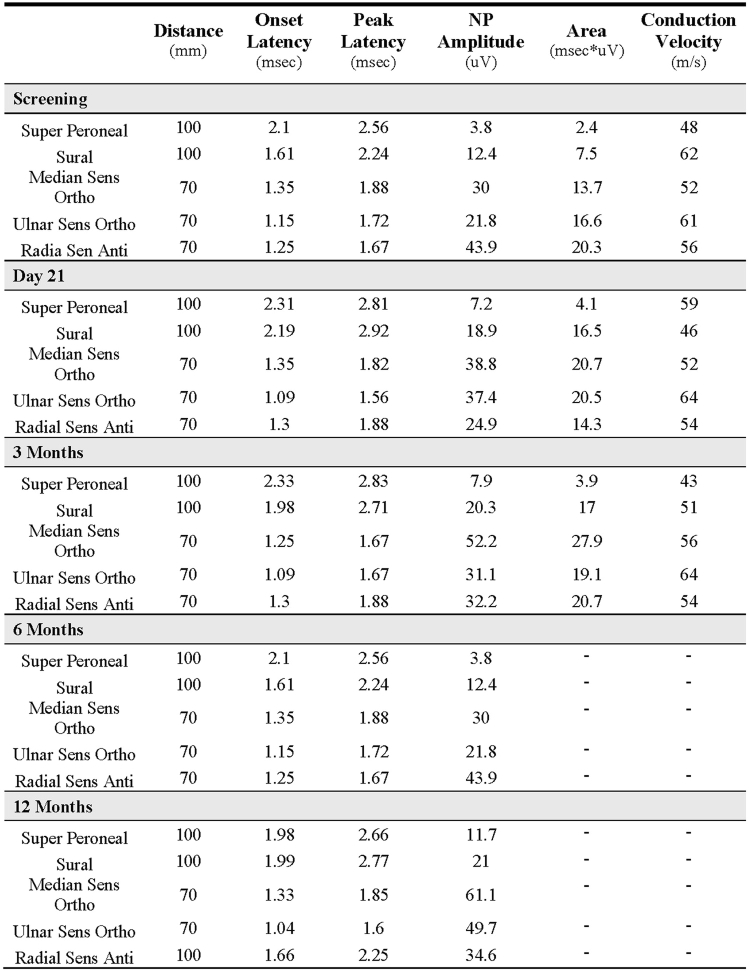
Sensory nerve analyses performed during the study. Standard nerve conduction studies were performed at baseline and then at 3 weeks, 3 months, 6 months, and 12 months. Presented are the data for the 5 sensory nerves that were studied. Values were within the normal range at all time points. Intriguingly, amplitudes increased post-dosing, suggesting, if anything, improvements in sensory nerve function. Of note, NCS was also performed on the Tibial motor nerve, and all values were within normal limits (data not shown).

No serious adverse events were detected through 12 months after dosing. Notable safety-related events are presented in Fig. 2a, with all safety data listed in Extended Data Figs. 3–5. Neutropenia was noted 6 days after dosing, which resolved without intervention within 1 week. At 5 months after dosing, the patient experienced severe abdominal discomfort, which has since resolved and was ultimately attributed to both *Clostridioides difficile* gastroenteritis and side effects of tacrolimus. We detected no clinical or electrophysiological evidence of dorsal root ganglion (DRG) toxicity; there were no neuropathic pain complaints, and the results of sensory examination and nerve conduction studies were normal (Extended Data Fig. 6). Contrast-enhanced brain and spine MRI at 3, 6 and 12 months after dosing showed no inflammatory changes and no progression in brain atrophy.

**Extended Data Fig. 7:**
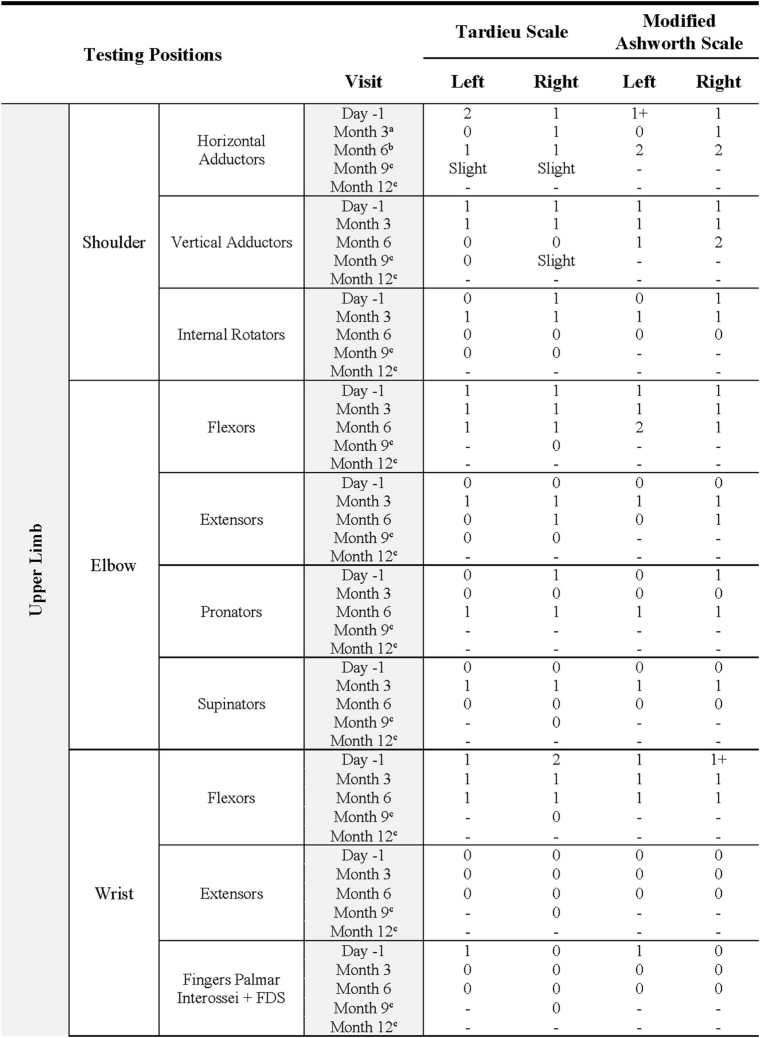
Scores of the Tardieu and modified Ashworth scales for the upper limbs. Scores from two measures of joint spasticity, the Tardieu and modified Ashworth scales. Existing natural history suggests worsening of spasticity in SPG50 over a 12-month period. We observed stabilization of scores on both scales, with no clear worsening. However, the patient poorly tolerated both outcome measures, resulting in missing data points at essentially all time points. Tardieu scale values are 0 = no resistance to passive movement, 1 = slight resistance, 2 = clear ‘catch’, interrupting passive movement, 3 = clonus ( < 10 seconds), 4 = sustained clonus. Ashworth scale values are 0 = no increase in tone, 1 = slight increase in tone, 1 + = slight increase in tone, catch/release through range of motion, 2 = marked increase in tone, 3 = marked increase in tone AND passive movement difficult, 4 = fixed contracture.

**Extended Data Fig. 8:**
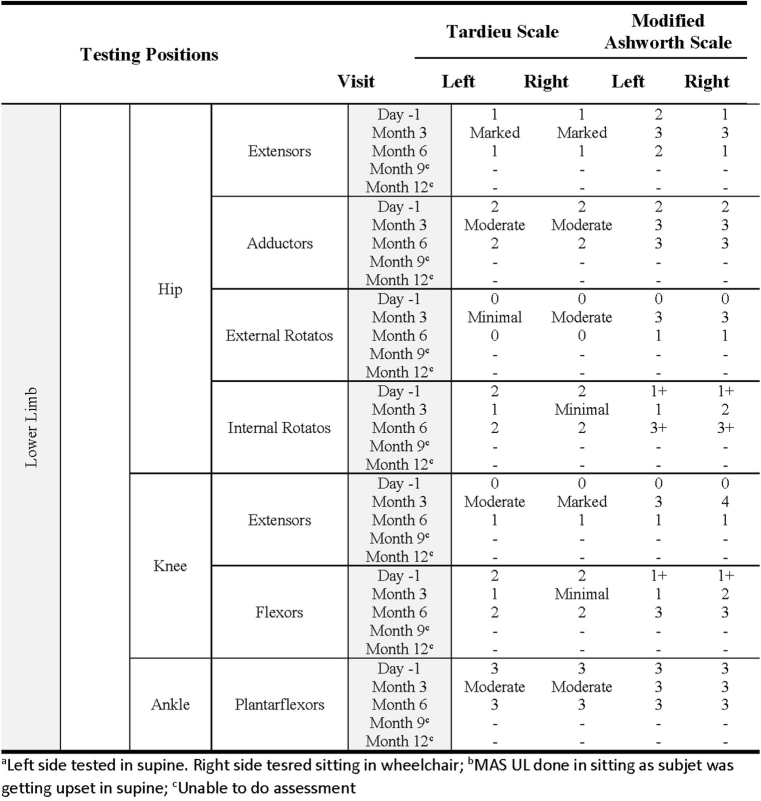
Scores of the Tardieu and modified Ashworth scales for the lower limbs. Scores for the lower limbs for the Tardieu and Ashworth scales. Score values are presented in Extended Data Figure 7.

Progressive limb spasticity is a major SPG50 disease component^11^. We measured spasticity using two scales previously developed for cerebral palsy: the Tardieu^16^ and modified Ashworth^17^ scales. These were not well tolerated (due to the patient’s discomfort with passive joint manipulation), and data points across several assessments are missing (Extended Data Figs. 7 and 8). However, compared to predosing assessments, there was no negative change in successfully scored joints.

**Extended Data Fig. 9:**
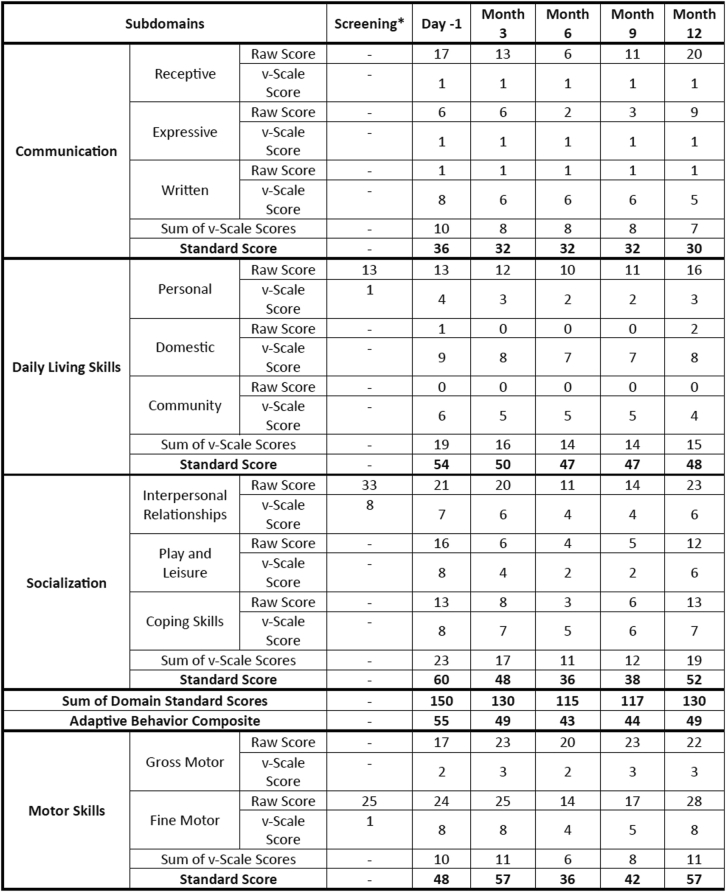
Vineland Adaptive Behavior Scale (version 2). Results from the Vineland adaptive behavior scale, parent reported. Substantial gains were noted in both gross and fine motor skills (from baseline of 48 composite to 57 at 12 months post dosing). Small declines in scores were noted in adaptive behavior. This may be related in part to non-Melpida related side effects, including prolonged gastroenteritis and abdominal pain secondary to tacrolimus, as well as social impacts of immune suppression (such as prolonged absence from school).

Developmental delay is also an important feature of SPG50. We examined this using two exploratory measures: the Bayley Scale of Infant and Toddler Development^18^ and the Vineland Adaptive Behavior Scale^19^. Bayley scores increased across multiple domains (Fig. 2b). Vineland scores were more variable, with a modest decline in adaptive behavior and improvements in motor domains (Fig. 2c and Extended Data Fig. 9).

At the time of the last examination, the patient was able to stand with his heels on the ground (Clinical Global Impression (CGI) of Improvement (CGI-I) level 3 = minimally improved; Methods)—something that had not been achieved before dosing—and to subjectively tolerate longer periods of standing in a stander and walking with an assist device. No subjective disease worsening or loss of skills was observed. The parent log data showed that, since receiving the therapy, the patient has not experienced falls or seizures. Before dosing, the patient had infrequent seizures (one seizure in the previous 24 months).

Overall, we describe the full development cycle of a single-patient gene therapy for SPG50. Typically, the implementation of new treatments comes too slowly to help the patient(s) that initially inspired them. This study represents an example of AAV-based gene therapy that was rapidly developed and administered in a timely fashion to benefit the original ‘inspirational’ patient. Therefore, it provides a potential road map for individualized genetic therapy for other ultra-rare disorders.

The primary outcome was safety, and no serious adverse events were identified despite the large dose of AAV administered intrathecally. Our immunosuppression protocol was more extensive than that used in many previous gene therapy trials, reflecting our concern about immune-mediated toxicities and our desire to promote lasting immune tolerance to the gene therapy product. While some observed side effects were attributable to immunosuppression, our patient also did not develop an anti-AP4M1 immune response. Determining whether this represents an optimal immunomodulation strategy for AAV9 gene therapy will require its use in additional patients and gene therapy programs. Of note, our patient experienced transient neutropenia and a T cell reaction to AAV9 (Extended Data Fig. 2), suggesting that some AAV9 had entered the systemic circulation.

Regarding efficacy, our assessments indicated possible disease stabilization after AAV9-*AP4M1* treatment. Based on existing natural history data, progression is anticipated over a 1-year period^3^. Thus, our data may represent a modification of the expected disease course.

A notable aspect of this study was its rapid development. The time from diagnosis to dosing was <3 years. The speed of development was aided by several factors, including the use of an existing AAV9-based gene therapy ‘template’ and collaboration between multiple researchers. This latter aspect was facilitated by the CureSPG50 Foundation, which nucleated the work and established connections between researchers, clinicians, contract research organizations and industry partners.

There may be opportunities to accelerate future projects further. Preclinical SPG50 models had to be established. For other diseases, these could be developed in advance of therapy conception. Toxicity experiments in nonhuman primates were strongly encouraged by regulatory agencies. As more gene therapy trials are successfully completed, the requirement for such studies may be reduced. None of the preclinically identified adverse findings presented in our patient, including DRG toxicity. This highlights a broader question of the predictiveness of animal studies for safety and toxicity, something that has come to light with other gene therapy programs, in which there has been safety signal discordance between animal toxicology studies and human clinical trials^20,21^.

The trial design was innovative although not unique, as other single-patient genetic therapy trials have been completed^22^. We used emerging data on the disease’s natural history combined with the patient’s pretreatment data to monitor and assess efficacy—a strategy potentially applicable to future studies. Spasticity was a challenging outcome to measure, particularly in this young, nonverbal patient who did not tolerate extensive direct examination. Therefore, existing scales may not be suitable for some patients with spastic paraplegia. One future outcome could be timed heel versus toe standing, as this reflects ankle spasticity and range of motion and has functional links with pathologic toe walking.

More generally, for single-patient trials, it is crucial to establish objective and easily measurable outcomes. In individuals with epilepsy or abnormal involuntary movements, quantification of seizures or movements can provide a robust measure of treatment response. Activity-monitoring wearables may also have a role, particularly in ambulant individuals. Early-phase studies can thus serve important value in identifying and testing outcome measures that inform subsequent pivotal trials. In our case, we enumerated a potential challenge with existing spasticity scales and identified a new possible outcome measure (maximally tolerated stand time). Through outcome assessments, small-*n* trials can additionally provide insights into which disease elements are modifiable, as it is likely that some aspects of a genetic disorder will not be amenable to intervention even when the treatment addresses the root cause of the disease.

It is important to emphasize the limitations of single-patient studies like this one. For instance, safety data from one individual may not generalize to a broader cohort and could potentially either provide false reassurance of safety or, conversely, overestimate the expectation of harm. This could lead in subsequent patients to unanticipated risk or, instead, premature discontinuation of a promising drug program. In terms of treatment effectiveness, in the absence of a pronounced deviation from the pretreatment baseline (such as a nonambulant individual obtaining the ability to walk), single-patient data are challenging to interpret. This is particularly true for a disorder like SPG50, the natural history of which is still being established. Small improvements may be missed or else overstated as treatment associated. Furthermore, in a slowly and variably progressive condition, it may take years in a single patient to understand whether progression has truly been modified.

There were several ethical considerations, particularly as the parent-created foundation provided substantial support to product development^23,24^. To evaluate these considerations, we established a special review committee. The committee (Methods) reviewed the protocol and study design and provided input on the handling of several topics, including informed consent and mitigation of bias. Subsequent to the trial, and based on the experiences gained during the process, we formalized this committee into our Advanced Therapeutics Review Board at the Hospital for Sick Children (SickKids), which now serves to address the ethical challenges of individualized therapy development for ultra-rare diseases.

A key aspect of this study is cost. The CureSPG50 Foundation estimated that the total cost of the project for preclinical development was Canadian $3,500,000, and the cost of the clinical trial was approximately $250,000, plus expenses related to concomitant medicines (tacrolimus and sirolimus) and in-kind contributions that were difficult to estimate. While our overall workflow provides a road map applicable to other genetic diseases, it is challenging (given the cost) to consider this as a widely iterative strategy for ultra-rare disease gene therapy. Cost-reducing innovations are clearly needed. Manufacturing expenses are extremely high, particularly related to batch production for small patient numbers. A paradigm leap in production is likely required to make gene therapy viable for the largest number of patients. More immediately, consideration of the required investigational new drug-enabling preclinical studies could aid in cost reduction. Large animal studies, in particular, are key drivers of cost and development time that may be unnecessary in settings like this (ultra-rare disease, high unmet need, use of an existing vector backbone).

In conclusion, we present an individualized gene therapy trial and outline a path for future similar studies for ultra-rare diseases. Subsequently, this study has motivated a larger United States-based trial to treat additional patients with SPG50 (NCT05518188).

## Methods

### Regulatory information and trial oversight

Approval to proceed (that is, a no objection letter) was obtained from Health Canada on 30 December 2021. The protocol (version 5) and supporting documentation were submitted to the SickKids Research Ethics Board (REB) on 7 January 2022. REB approval (REB no. 1000079110) was obtained on 15 February 2022. Protocol version 5 established the inclusion/exclusion criteria and prespecified all safety and efficacy outcome measures. Recruitment for the trial was opened at the time of the approval of protocol version 5. Subsequent amendments (versions 5.1 and 6) to this protocol addressed minor changes to the immunosuppression regimen, minor clarifications to the Bayley scale (removal of the Growth Scale Value score) and reporting change for the Vineland scale (switch from examiner to caregiver reporting).

Before submission to Health Canada, a review of the proposed study was completed by an internal ethics committee. This committee included the chair of the REB, an expert bioethicist, in-house legal counsel and members of the hospital executive leadership. The committee discussed the challenges posed by this single-patient study, including issues related to conflicts of interest and informed consent. Study submission proceeded after committee evaluation and incorporation of guidance related to trial elements, including consent and monitoring.

Informed consent was obtained following the standard operating procedure set by SickKids. Capacity assessment of the participant was completed by the study doctor. Appropriately delegated research study team members discussed the informed consent statement with both parents. The study doctor was not present during the signing of the consent form (to avoid undue influence) but was available for discussion and clarification. Ample time was provided for the family to ask questions and consider the trial. Upon discussion, the consent form was signed by the delegated study coordinator and a parent on 11 March 2022. The capacity to consent is assessed by the study doctor on an ongoing basis. If and when applicable, appropriate assent or consent will be obtained from the study participant.

Throughout the study, study conduct and data were monitored by the Clinical Research Quality and Education Board at SickKids.

Per Health Canada specifications, registration of trials in a public database is encouraged. Owing to our uncertainty at the time of obtaining the no objection letter regarding single-patient studies, the study was initially not registered. It was retrospectively registered at ClinicalTrials.gov in October 2023 (NCT06069687).

### Vector design, manufacturing and dosing

**Extended Data Fig. 10:**
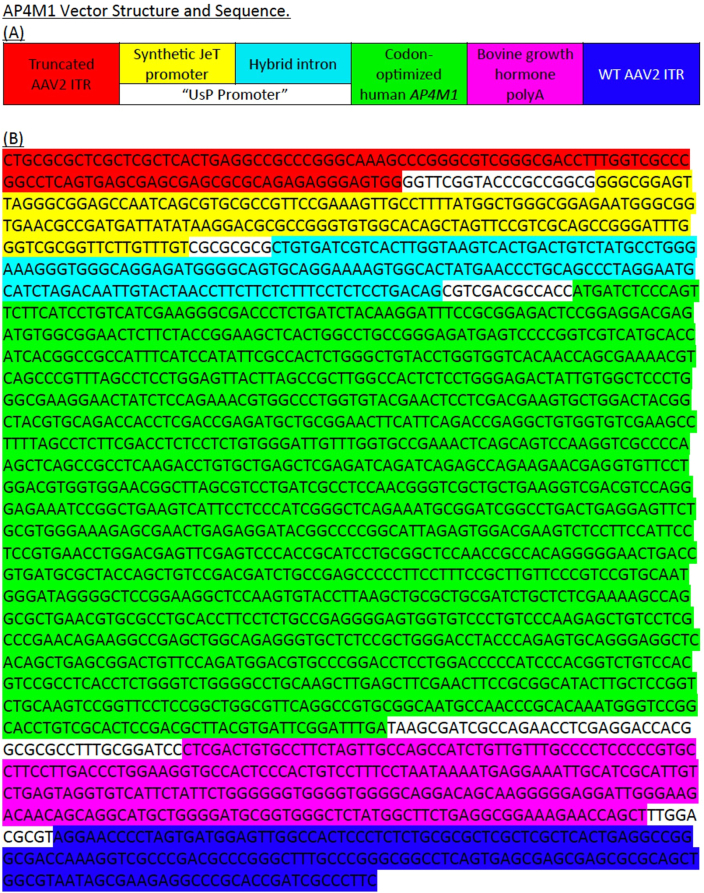
AP4M1 vector structure and sequence. Melpida consists of a vector containing codon optimized AP4M1 and surrounding sequences (UsP promoter and bGH polyA tail) inserted between truncated AAV2 ITR sequences and encapsulated in AAV9. (**a**) Schematic of the vector structure. (**b**) Sequence of the vector.

The design of AAV9-*AP4M1* has been described previously^14^. The vector structure and sequence are presented in Extended Data Fig. 10. The clinical AAV9-*AP4M1* vector (MELPIDA) was manufactured by Viralgen in accordance with current Good Manufacturing Practice standards. Briefly, it was manufactured using Viralgen’s proprietary process involving triple-plasmid transfection into suspension HEK293 cells, followed by downstream processing to remove impurities and enrich for genome-containing AAV particles. The final solution of AAV9-*AP4M1* was formulated in PBS containing 5% d-sorbitol and 0.001% pluronic F68. The final certificate of analysis is provided as Supplementary Data. A total dose of 1 × 10^15^ vg was delivered to the patient over 10 min at a volume of 10 ml by lumbar intrathecal administration with the patient in 15° Trendelenburg positioning (head down). The patient was maintained in the Trendelenburg position for 1 h after infusion. The dose was derived from preclinical studies and extrapolated from calculations of normative CSF volumes.

### Study objectives

The primary objective of this study was to evaluate the safety and tolerability of a single dose of AAV-AP4M1 (that is, MELPIDA) administered intrathecally to a single child with SPG50. Safety was evaluated as described below; the evaluation included serum studies related to hematologic, immune and liver function and/or injury, as well as assessment of DRG toxicity by nerve conduction studies. The secondary objective was to assess efficacy, which was determined by examining the patient for stability or improvement in spasticity (as assessed using the modified Ashworth and Tardieu scales).

Exploratory assessments included measurement of AAV9 antibody titers, evaluation of T cell responses to AAV9 and A4PM1 by whole-blood ELISpot assay, evaluations based on rating scales (Vineland Adaptive Behavior Scale (Comprehensive Parent/Caregiver Form), CGI of Overall Change by Physician, Bayley Scale of Infant and Toddler Development (fourth edition)), and use of logbooks to record the number and duration of seizures and falls daily.

The CGI assesses changes from the pretreatment baseline (CGI-I) and the severity of the current illness (CGI-S). CGI-I is a seven-point scale (1 = very much improved, 2 = much improved, 3 = minimally improved, 4 = no change, 5 = minimally worse, 6 = much worse and 7 = very much worst). CGI-S is also a seven-point scale (1 = normal (shows no signs of illness), 2 = borderline ill, 3 = slightly ill, 4 = moderately ill, 5 = markedly ill, 6 = severely ill and 7 = among the most extremely ill of patients).

### Inclusion and exclusion criteria

#### Inclusion criteria

- Age <5 years
- Confirmed diagnosis of SPG50 disease by
  - Genomic DNA mutation analysis demonstrating homozygous or compound heterozygous, pathogenic and/or likely pathogenic variants in the *AP4M1* gene
  - Clinical history or physical examination consistent with SPG50
- Parent/legal guardian willing to provide written informed consent for their child before study participation
- Patient able to comply with all protocol requirements and procedures

#### Exclusion criteria

- Inability of the patient to participate in study procedures, as determined by the site investigator
- Presence of a concomitant medical condition that precludes lumbar puncture (LP) or use of anesthetics
- History of a bleeding disorder or any other medical condition or circumstance in which LP is contraindicated according to local institutional policy
- Inability of the patient to be safely sedated, in the opinion of the clinical anesthesiologist
- Active infection at the time of dosing, based on clinical observations
- Concomitant illness or requirement for chronic drug treatment that, in the opinion of the principal investigator, creates unnecessary risks for gene transfer
- Inability of the patient to undergo MRI according to local institutional policy
- Inability of the patient to undergo any other procedure required in this study
- Presence of non-SPG50-related CNS impairment or behavioral disturbances that would confound the scientific rigor or the interpretation of the study results
- Received an investigational drug within 30 days before screening or plan to receive an investigational drug (other than gene therapy) during the study
- Enrollment and participation in another interventional clinical trial
- Contraindication to AAV-AP4M1 or any of its ingredients
- Contraindication to any of the immunosuppressive medications used in this study
- Clinically significant abnormal laboratory values (γ-glutamyl transferase (GGT), alanine aminotransferase (ALT) and aspartate aminotransferase (AST) or total bilirubin more than three times the upper limit of normal, creatinine ≥1.5 mg dl^−1^, hemoglobin <6 or >20 g dl^−1^, white blood cell count >20,000 per mm^3^) before therapy

#### Study procedure

- Study initiation. A potential participant was identified. The study team presented the study to the participant’s parents, and forms were given to the family for review. Time was provided for questions and study review. After discussion and consideration, the delegated study coordinator obtained verbal and written informed consent from the participant’s parents on 11 March 2022.
- Screening visit. A ‘screening visit’ was conducted. The screening visit (−28 to −8 days before vector infusion) included confirmation of the genetic diagnosis, review of medical history and concomitant medications, a complete physical examination, vital sign assessment, height and weight measurements, 15-lead electrocardiography, liver ultrasonography, blood and urine collections for safety laboratory tests, and spasticity assessments (modified Ashworth and Tardieu scales) performed by a trained examiner.
- Safety laboratory tests. These tests included complete blood count with differential, coagulation tests (international normalized ratio, prothrombin time, partial thromboplastin time), erythrocyte sedimentation rate, C-reactive protein, Na, K, Cl, Ca, CO_2_, blood urea nitrogen, creatinine, glucose, ALT, AST, total/direct/indirect bilirubin, alkaline phosphatase, GGT, serum total protein, cardiac safety panel (troponin, pro-B-type natriuretic peptide, creatine kinase isotype MB) and urinalysis (for protein, cells, glucose and bacteria). Laboratory samples were drawn at −28, −7 and −1 days before dosing and 2 days, 7 days, 14 days, 21 days, 28 days, 3 months, 6 months, 9 months and 12 months after dosing.
- Dosing. Dosing was accomplished through infusion into the intrathecal space. LP was performed through interventional radiology with an anesthesiologist present to administer sedation before infusion. The participant was placed in the Trendelenburg position (head down). An atraumatic Sprotte needle (Pajunk, item no. 321151-31A) was inserted percutaneously at the lumbar level L4/L5 interspace. Needle placement was confirmed with fluoroscopic intraoperative imaging before and after administration. Before infusion, 10 ml of CSF was withdrawn from the lumbar space. MELPIDA solution was loaded into a 20-ml BD syringe connected to the needle with 60-inch mini-volume intravenous extension tubing and a Braun four-way stopcock. The infusion was administered at a rate of 1 ml min^−1^, for a total of 10 ml, using a CareFusion Alaris 8110 syringe pump. Following administration, the participant remained in the Trendelenburg position (head down) for 1 h with turning (left to right, right to left) every 15 min. In addition, vital signs, including heart rate, respiratory rate, blood pressure and pulse oximetry, were monitored continuously for 1 h and then every 15 min until 2 h after infusion, every 30 min for the following 2 h (third and fourth hour following infusion), hourly for an additional 4 h and subsequently every 4 h until discharge. The patient was discharged without complications on the day following MELPIDA administration.
- Immunosuppression. Three immunosuppressive agents were used (sirolimus, tacrolimus and prednisone). Sirolimus was initiated 1 week before infusion, with an initial load of 1 mg m^−2^ every 4 h for three doses, followed by daily enteral dosing at 1 mg m^−2^ per day divided two times a day. Levels were checked after 5 days of treatment and deemed to be within the acceptable range; thus, the therapy was continued at this dose. Prednisone (1 mg kg^−1^ per day) and tacrolimus (0.2 mg kg^−1^ per day divided two times a day) were started 1 day after infusion. The levels of both tacrolimus and sirolimus were monitored monthly. At 3 months, prednisone taper was started, with completion in 4 weeks. At 6 months, tacrolimus taper was initiated, with completion in 4 weeks. Both tapers were initiated after a review of brain MRI and CSF analysis results confirmed no concern for active infection or inflammation. Sirolimus wean is planned to start at 18 months.
- Postdosing assessments. At 7, 14, 21 and 28 days after infusion, the participant was brought on-site for a review of vital signs, safety laboratory tests, brief physical examination, collection of viral shedding samples, documentation of concomitant medications and enumeration of any adverse events. On days 7 and 21, exploratory laboratory tests were performed. In addition, on day 21, nerve conduction studies were performed, and on day 28 a comprehensive neurological physical examination was completed. At 3, 6, 9 and 12 months, the participant was assessed for all outcome measures. In addition, as a safety measure to monitor for CNS inflammation or infection, brain and spine MRI (with and without gadolinium) and an LP for CSF analysis were performed at baseline, 3, 6 and 12 months. For all LPs, a 21-gauge standard LP needle was used. EMLA (a eutectic mixture of local anesthetics) was applied for local anesthesia, and then the LP needle was inserted into the intrathecal space between L4/L5. An appropriate quantity of CSF was removed for relevant safety laboratory studies (complete blood cell count with differential, protein, glucose, bacterial culture). Liver ultrasonography was conducted at 6 and 12 months. Nerve conduction studies were performed at 3, 6 and 12 months. Nerve conduction studies and brain MRI are planned at 18 months and 2 years after dosing and then yearly thereafter. An additional LP will be performed at 18 months, before the planned sirolimus wean.
- Documentation. Adverse events and concomitant medications were monitored on a continuous basis over the course of enrollment and reviewed at each study visit. Any adverse events were reported and documented in a timely manner and in accordance with the regulatory requirements of SickKids and Health Canada. Data collection began at the time of informed consent signing. Source data included all information, original records of clinical findings, observations and all clinical trial activities, as necessary for the reconstruction and evaluation of the trial. Electronic case report forms were used to collect and store all study data in addition to maintenance of the original source documentation. The electronic data capture platform used was REDCap. Interim analyses were performed at 6 and 12 months after dosing and are planned for yearly thereafter up to 5 years. As presented in section 9.1 of the protocol (‘Database locks’ in Supplementary Information), interim analyses were prespecified to be performed at periodic intervals per the judgment of the study team (to review ‘key deliverables requiring analysis’).

### Sex and gender as biologic variables

Given that this was a single-patient study (one male participant), we are not able to adequately study or make conclusions regarding the potential impact of sex and/or gender on SPG50 and AAV9-*AP4M1* gene therapy.

### Reporting summary

Further information on research design is available in the Nature Portfolio Reporting Summary linked to this article.

## Online content

Any methods, additional references, Nature Portfolio reporting summaries, source data, extended data, supplementary information, acknowledgements, peer review information; details of author contributions and competing interests; and statements of data and code availability are available at 10.1038/s41591-024-03078-4.

### Supplementary information

Supplementary Information Clinical trial protocol v.5.0 and v.6.0.

Reporting Summary

Supplementary Data MELPIDA certificate of analysis.

## Data Availability

All data relevant to supporting the findings reported in this study are available within the paper and in the Supplementary Information. Restrictions apply to some information related to the study, which are protected per institutional review board requirements. The sequence and structure of MELPIDA are included as Extended Data Fig. 10. For all data inquiries, please contact Ana Stosic (ana.stosic@sickkids.ca) and/or James Dowling (james.dowling@sickkids.ca). Data or material transfer agreements may be required and will be assessed at the time of request (approximate timeline for review = 8 weeks).
