# Supplementary material for: AAV gene therapy for hereditary spastic paraplegia type 50: a phase 1 trial in a single patient
Source: Nat Med. 2024 Jun 28;30(7):1882–7. doi: 10.1038/s41591-024-03078-4 (PMC11271397; doi:10.1038/s41591-024-03078-4)
Supplement: Supplementary file 1 — Clinical trial protocol v.5.0 and v.6.0. [file 41591_2024_3078_MOESM1_ESM.pdf]

nature medicine

Supplementary information https://doi.org/10.1038/s41591-024-03078-4

AAV gene therapy for hereditary spastic paraplegia type 50: a phase 1 trial in a single patient

In the format provided by the authors and unedited

Clinical Trial Protocol

A Phase 1 Open-label Intrathecal Administration of MELPIDA to Determine the Safety and Efficacy for Patients with Spastic Paraplegia Type 50 (SPG50) caused by a Mutation in the AP4M1 gene.

**Clinical Trial Protocol #:**	MELPIDA for SPG50
**Protocol Version #:**	5.0
**Protocol Date:**	December 23rd 2021
**Phase of Study:**	Phase I
**Sponsor:**	James Dowling, MD, PhD and<br>The Hospital for Sick Children
**Sponsor Address:**	The Hospital for Sick Children<br>555 University Avenue<br>Toronto, ON Canada M5G 1X8
**HC**	TBD
**FDA IND**	TBD

CONFIDENTIALITY STATEMENT

This document contains confidential information, which should not be copied, referred to, released or published without written approval from The Hospital for Sick Children and James Dowling. Investigators are cautioned that the information given in this brochure might be subject to change and revision. Any conclusion regarding efficacy and safety must be considered provisional.

SIGNATURE PAGE AND REVISION HISTORY

Dr J Dowling, MD Date

The Hospital for Sick Children
555 University Ave
Toronto, ON
M5G 1X8

Amendments: The following sections/appendices have been updated:

Section/Appendix	Description	Date
Amendment 1 [date]		

TABLE OF CONTENTS

1. STUDY SYNOPSIS 7

2. INTRODUCTION AND BACKGROUND 9

3. RATIONALE FOR THE STUDY 10

4. STUDY OBJECTIVES & ENDPOINTS 11
4.1. Primary Objectives 11
4.2. Primary EndpPoints 11
4.3. Secondary Objectives 11
4.4. Secondary Endpoints 11
4.5. Exploratory EndpointsObjectives 11

5. INVESTIGATIONAL PLAN 12
5.1. Study Design 12
5.2. Investigational Product 12
5.3. Packaging 12
5.4. Labeling and Storage 12
5.5. Dose and Route 12
5.5.1. Dose Selection Rationale 13

6. SCHEDULE OF EVENTS 18
6.1. Screening: 18
6.2. Enrollment and pre-dosing schedule: 20
6.3. Dosing day: 20
6.3.1. Administration of Study intervention (MELPIDA): 20
6.3.2. Anesthesia Safety 21
6.3.3. Post-Procedure Recovery 21
6.4. Day 2 21
6.5. Days 7, 14, 21, 28 (+/- 2 days) 22
6.6. Months 3, 6, 9 and 12 (+/- 14 days) 22
6.7. Months 18, 24, 36, 48, 60 (+/- 14 days) 22
6.8. Specific Study Procedures 25
6.8.1. Nerve conduction study (NCS) 25
6.8.2. Magnetic Resonance Imaging of Brain (MRI) 25
6.8.3. Liver Ultrasound 25
6.8.4. Lumbar puncture 25
6.9. Adverse Event and Concomitant Medication Monitoring 26
6.10. Immune Modulation Protocol 26

7. INCLUSION/EXCLUSION CRITERIA 27
7.1. Inclusion Criteria 27
7.2. Exclusion Criteria 28
7.3. Participant Withdrawal 29

8. STATISTICAL ANALYSIS 29
8.1. Data Monitoring 29
8.1.1. General Plan 29
8.1.2. Monitoring Entity 30

8.2. Plans for Assuring Participant Safety, Adverse Event Collection, and Reporting 30
8.3. Definitions 30
8.3.1. Adverse Event 30
8.3.2. Classification of Adverse Events 31
8.4. Dose Limiting Toxicity 33
8.5. Reporting Procedures to the REB 33
8.6. Reporting Procedures to Health Canada 33
8.7. Reporting Procedures to the Research Ethics Board 34
8.8. Protocol Deviations and Continuing Review 35
8.9. Stopping Rules 35

9. DATA COLLECTION 36
9.1. Database Locks 36
9.2. Study Monitoring Plan 36
9.3. Quality Assurance of Data 37

10. INSTRUMENTS FOR THE ASSESSMENT OF DISEASE 37
10.1. Modified Ashworth Scale 37
10.2. Tardieu Scale 38

11. PRIVACY AND CONFIDENTIALITY 40

12. REFERENCES 41

List of Abbreviations

AAV	adeno-associated virus
Ab	antibody
AE	Adverse event
Ag	antigen
ALT	Alanine aminotransferase
ALP	Alkaline phosphatase
AP-4	Adaptor Protein complex
AP4M1	Adaptor protein complex, μ4
AST	Aspartate aminotransferase
ATG9A	Autophagy Related 9A
BCH	Boston Children's Hospital
BGH	Bovine growth hormone
BUN	Blood urea nitrogen
Ca	calcium
CBC	Complete blood counts
CDMO	Contract development and manufacturing organization
CK-MB	Creatine kinase – isotype MB
Cl	chloride
CMC	Chemistry, manufacturing and controls
CNS	Central nervous system
CO_2	Carbon dioxide
Cr	Creatinine
CRP	C-reactive protein
CSF	Cerebrospinal fluid
CTA	Clinical Trial Application
DAPI	4′,6-diamidino-2-phenylindole
DNA	Deoxyribonucleic acid
DTI	Diffusion tensor imaging
EEG	electroencephalogram
EKG	electrocardiogram
ESR	Erythrocyte sedimentation rate
FDA	Food and Drug Administration
GFP	Green fluorescent protein
GGT	Gamma-glutamyl transferase
GLP	Good laboratory practice
h	Human
hAP4M1opt	Human optimized AP4M1
HSP	Hereditary spastic paraplegia
HC	Health Canada
HCT	hematocrit
HIV	Human immunodeficiency virus
HTLV1	Human T-Lymphotropic Virus Type 1
IND	Investigational New Drug
INR	International normalized ratio
iPSCs	Induced pluripotent stem cells
IRNHS	International registry Natural History Study
IT	Intrathecal

ITR	Inverted terminal repeat(s)
K	potassium
kg	Kilogram
LFT	Liver function tests
LP	Lumbar puncture
MCV	Mean corpuscular volume
MOI	Multiplicity of Infection
MRI	Magnetic resonance imaging
Na	sodium
NCS	Nerve conduction studies
NAb	Neutralizing antibody
NHP	Non human primates
NIH	National Institutes of Health
opt	Optimized
OOPD	Office of Orphan Products Development
PACU	Post anesthesia care unit
PBMC	Peripheral blood mononuclear cell
PI	Principal investigator
PICU	Pediatric intensive care unit
PLT	platelets
PPD	Purified protein derivative
ProBNP	Pro B-type Natriuretic peptide
PT	Prothrombin time
PTT	Partial Prothrombin time
sc	Self-complimentary
SickKids	The Hospital for Sick Children
SPG50	Spastic Paraplegia 50
RNA	Ribonucleic acid
SAE	Serious adverse event
SUSAR	Suspected unexpected serious adverse reaction
TB	tuberculosis
TGN	Trans-Golgi network
Tn	troponin
US	Unites States of America
UTSW	University of Texas Southwestern Medical Center
VCC	Viralgen Vector Core, Spain
vg	Vector genome(s)
WT	Wild type

1. STUDY SYNOPSIS

Title	A Phase 1 Open-label Intrathecal Administration of MELPIDA to Determine its Safety and Efficacy for Patients with Spastic Paraplegia Type 50 (SPG50) caused by Mutation in the AP4M1 gene.
Study Description	This will be a first-in-human Phase I, open-label, single dose clinical study of MELPIDA administered intrathecally (IT) through a lumbar puncture (LP) to a single subject with confirmed pathogenic mutations in the AP4M1 gene.
Number of Subjects	N = 1
Clinical Study Phase	Phase I
Sponsor	Dr James Dowling and The Hospital for Sick Children, Toronto
PI	Dr. James Dowling
Study Objectives	Primary outcome: determination of the safety and tolerability of MELPIDA in patients with SPG50, based on development of toxicity<br>Secondary outcome: preliminary exploration of efficacy
Study Intervention	MELPIDA, a recombinant serotype 9 adeno-associated virus (AAV) encoding a codon-optimized human AP4M1 transgene
Study Dose	A single intrathecal infusion of 10 mL at 1E14 vg/mL for a total dose of 1E15 vg
Study Population	Children with a confirmed mutation in the AP4M1 gene
Study Duration	The total study duration is 5 years post dosing.<br>The participant will be tested at screening/baseline (-28 to -7 days), return for dosing, and then follow-up visits post-dosing on Days 7 (+/-2), 30 (+/-2), 60 (+/-2), 90 (+/-14), 180 (+/-14), 270 (+/-14), 360 (+/-14), 540 (+/-14), and 720 (+/-14) days, then annually for the last 3 years.
Inclusion Criteria	• Age < 5 years old<br>• Confirmed diagnosis of SPG50 disease by:<br>• Genomic DNA mutation analysis demonstrating homozygous or compound heterozygous, pathogenic and/or potentially pathogenic variants in the *AP4M1* gene<br>• Clinical history or examination features consistent with SPG50 and that include neurologic dysfunction<br>• Parent/legal guardian willing to provide written informed consent for their child prior to participation in the study<br>• Subject able to comply with all protocol requirements and procedures
Exclusion Criteria	• Inability to participate in study procedures (as determined by the site investigator)<br>• Presence of a concomitant medical condition that precludes lumbar puncture (LP) or use of anesthetics<br>• History of bleeding disorder or any other medical condition or circumstance in which lumbar puncture is contraindicated according to local institutional policy<br>• Inability to be safely sedated in the opinion of the clinical anesthesiologist<br>• Active infection, at the time of dosing, based on clinical observations

	• Concomitant illness or requirement for chronic drug treatment that in the opinion of the PI creates unnecessary risks for gene transfer<br>• Inability of the patient to undergo MRI according to local institutional policy<br>• Inability of the patient to undergo any other procedure required in this study<br>• The presence of significant non-SPG50 related CNS impairment or behavioral disturbances that would confound the scientific rigor or interpretation of results of the study<br>• Have received an investigational drug within 30 days prior to screening or plan to receive an investigational drug (other than gene therapy) during the study.<br>• Enrollment and participation in another interventional clinical trial<br>• Contraindication to MELPIDA or any of its ingredients<br>• Contraindication to any of the immune suppression medications used in this study<br>• Clinically significant abnormal laboratory values (GGT, ALT, and AST, or total bilirubin > 3 × ULN, creatinine ≥ 1.5 mg/dL, hemoglobin [Hgb] < 6 or > 20 g/dL; white blood cell [WBC] > 20,000 per cmm) prior to gene replacement therapy. Patients with an elevated bilirubin level that is unequivocally the result of neonatal jaundice shall not be excluded
Study Design	This will be a first-in-human Phase I, open-label, single dose clinical study of MELPIDA administered intrathecally through a lumbar puncture to a single subject with confirmed pathogenic mutations in the AP4M1 gene.
Primary Endpoints	Incidence of anticipated treatment-related toxicities, Grade 3 or higher, will be determined from the collection of occurrence and severity of serious adverse events (SAEs). In addition, change from baseline in nerve conduction velocity and amplitude, and determination of liver safety (e.g. laboratory and image studies) will be conducted at timepoints listed in the Schedule Of Events
Secondary Endpoints	Efficacy will be determined by the stability or improvement in spasticity as assessed using the Modified Ashworth scale (MAS) and Tardieu scale.
Exploratory Endpoints	• Bayley Scales of Infant and Toddler Development 4th edition (Growth Scale Value) (Fine Motor & Activities of Daily Living)<br>• Vineland (Inter-personal Domain, Fine Motor Domain, Personal Domain)<br>• Log Book of Seizure frequency and duration<br>• Log Book of Number of Falls<br>• Clinical Global Impression of Overall Change by Physician (CGI)
Sample Size	This is a single dose study in a single subject

The Schedule of Events can be found in Table 3.

2. INTRODUCTION AND BACKGROUND

MELPIDA is a gene therapy product being developed for the treatment of Spastic Paraplegia Type 50 (SPG50), which is one of a group of four genetic disorders (SPG47, SPG50, SPG51 and SPG52) comprising AP-4 related Spastic Paraplegia (AP4-SPG). Inherited in an autosomal recessive pattern, AP-4- SPG is caused by biallelic pathogenic variants in one of 4 genes that encode components of the heterotrimeric adaptor protein complex 4 (AP4). Mutations in any of the components result in disrupted AP-4 function, and result in a common, shared clinical phenotype (Behne et al, 2020; Ebrahimi-Fakhari et al, 2020; Ebrahimi-Fakhari et al, 2018). Adaptor protein complexes such as AP-4 play key roles in signal-mediated trafficking of integral membrane proteins. They mediate vesicle formation and the cargo contained within these vesicles (Jamra et al, 2011). While the precise function of the AP-4 complex is not fully understood, recent data suggests it plays an important role in protein sorting through the golgi, including regulation of trafficking of components required for autophagy (Davies et al., 2018). Deficiency in AP-4 leads to progressive neurodegeneration.

AP-4-HSP is an ultra-rare autosomal recessive disease with ~156 patients identified worldwide, 59 of which have the SPG50 subtype. There are approximately 9 patients with SPG50 in North America (OMIM #612936) (source: Ebrahimi-Fakhari et al, 2020), ClinicalTrials.gov Identifier: NCT04712812. SPG50 is caused by biallelic pathogenic variants in the AP4M1 gene.

The AP4-deficiency syndrome (AP-4-HSP) is characterized by progressive spasticity, microcephaly, intellectual deficiency, dysmorphic traits, and growth retardation (Roubertie et al., 2018). Symptoms of AP-4-HSP begin in infancy, though patients are often not correctly identified and diagnosed until age 5 to 10 years. Patients experience progressive spastic paraplegia in the first decade of life, resulting in quadriplegia by adolescence or early adulthood with associated wheelchair dependence. There is also the presence of severe, progressive cognitive impairment. Epilepsy is an important co-morbidity present in the majority of cases (Ebrahimi-Fakhari et al, 2018). Only a few affected individuals have been identified to survive beyond age 30 year, though the extent of early mortality is yet to be fully elucidated (Ebrahimi-Fakhari et al, 2020).

Based on an AP-4-HSP natural history study currently in progress at Boston Children’s Hospital (BCH), it is evident that disease severity ranges from child to child, but that most children fall into the severely affected (i.e. severe spasticity with paralysis and severe cognitive impairment) category. A small proportion of children, considered least severe, are able to speak in short sentences, walk with an abnormal gait, and have few to no seizures early on in the disease (less than 10 years of age). However, most children in this less severe category still experience progressive decline, ultimately losing the ability to walk and becoming quadriplegic between the ages of 10 and 20 years.

The majority of children with the SPG50 subtype of AP-4-HSP conform to a severe presentation, and are completely non-verbal, have microcephaly, never walk, have epilepsy and are severely cognitively impaired by the age of 10. It is not known how patients are affected later in life as very few have been identified beyond the age of 30. SPG50 is thus a degenerative neurological disease, affecting both cognitive and motor capabilities. Importantly, there is significant care

giver burden, as all patients eventually require complete support for all activities of daily living from family and/or caregivers. There are no treatments currently available for patients with SPG50.

Additional details on the disease pathophysiology and its progression are available in the Investigator's Brochure (Section 2).

3. RATIONALE FOR THE STUDY

SPG50 is a monogenetic disease caused by biallelic variants in the *AP4M1* gene. *APM41* encodes a subunit of the AP complex; mutations in *APM41* result in failure to produce functional APM41 protein and impairment of the AP complex. Due to the small size of the *APM41* gene, and the loss of expression/function nature of AP4M1 mutations, SPG50 is ideally suited for gene replacement therapy. MELPIDA is a recombinant serotype 9 adeno-associated virus (AAV9) encoding a codon-optimized human AP4M1 transgene (Figure 1). The final product consists of AAV9 capsids that are packaged with the self-complementary AAV genome comprising a mutant AAV2 inverted terminal repeat (ITR) with the D element deleted, the synthetic "UsP" promoter, codon-optimized human AP4M1 deoxyribonucleic acid (DNA) coding sequence, the bovine growth hormone (BGH) polyadenylation signal, and wild-type AAV2 ITR. As a gene therapy, MELPIDA is expected to provide a fully functional human AP4M1 cDNA copy to targeted neuronal and non-neuronal cells of the participant. Production of a fully functional AP4M1 subunit is hypothesized to halt neurodegeneration through the production of functional AP complex.

Figure 1 Schematic of MELPIDA

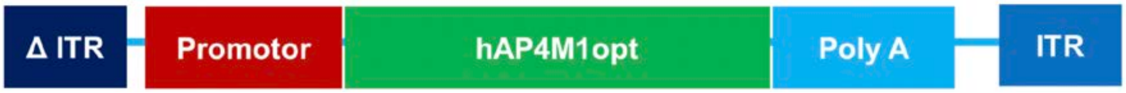

MELPIDA is an AAV9-based gene therapy vector that expresses the fully functional form of AP4M1 under thecontrol of a synthetic promoter. MELPIDA will be delivered intrathecally and is designed to achieve stable, potentially life-long expression of AP4M1 in non-dividing cells. This clinical study is a first-in-human study designed to assess safety and tolerability of MELPIDA in SPG50 participants, as well as examine the clinical impact of the gene therapy on disease progression.

Numerous investigators have utilized recombinant AAV9 directed at the central nervous system (CNS) in on-going gene therapy clinical trials (clinical trial.gov identifiers NCT02122952, NCT02362438, NCT02725580,NCT02716246, NCT03315182). These vectors are non-pathogenic, non-replicating, and transduce non-dividing cells. However, the recombinant vectors are incapable of coding viral proteins or actively integrating with the hostgenome, making them

ideal vectors for gene delivery. Additionally, AAV9 can be purified in large quantities at high concentrations for potential use in delivering a functional copy of a gene to cells with aberrant, disease-causing mutations. In disorders of neurologic origin, targeted CNS-focused administration (via intrathecal administration) achieves broad transgene distribution throughout the CNS. An approach utilizing intrathecal (IT) administration of AAV9 was first advanced as a treatment of Giant Axonal Neuropathy (GAN). The laboratory of Dr. Steven Gray, in partnership with Hannah's Hope Fund, initiated the first intrathecal AAV9 gene therapy trial, which was a first-in-human Phase I gene therapy clinical trial for GAN, in collaboration with Dr. Carsten Bonnemann at the US National Institutes of Health Clinical Center (NCT02362438) in 2015.

There are no approved treatments for SPG50, leaving an unmet medical need for this serious, rare, progressive, and ultimately fatal neurodegenerative disease.

4. STUDY OBJECTIVES & ENDPOINTS

4.1. Primary Objectives

The primary objective of this study is to evaluate the safety and tolerability of a single dose of MELPIDA administered intrathecally to a single child with SPG50 disease.

4.2. Primary Endpoints

Incidence of unanticipated anticipated treatment-related toxicities, Grade 3 or higher, will be determined from the collection of occurrence and severity of serious adverse events (SAEs). In addition, change from baseline in nerve conduction velocity and amplitude, and determination of liver safety (e.g. laboratory and image studies) will be conducted at timepoints listed in the Schedule Of Events

4.3. Secondary Objectives

The secondary objectives will be efficacy of the drug.

4.4. Secondary Endpoints

Efficacy will be determined by the stability or improvement in spasticity as assessed using the Modified Ashworth scale (MAS) and Tardieu scale. These assessments are summarized in the Table 1 and explained in section 6.4.2. 6.0 Investigational Plan

4.5. Exploratory Endpoints

Participants will undergo motor function, neuropsychological, and disease burden assessments every 3 months starting at screening/baseline to 24 months, then annually until 5 years post-dose. Additional assessments to evaluated as exploratory objectives include:

- Bayley 4 (Growth Scale Value) (Fine Motor & ADLS)
- Vineland (Inter-personal Domain, Fine Motor Domain, Personal Domain)
- Log Book Seizures
- Log Book # Of Falls
- Clinical Global Impression of Overall Change by Physician (CGI)

5. INVESTIGATIONAL PLAN

5.1. Study Design

This will be a first-in-human Phase I, open-label, single dose clinical study of MELPIDA administered intrathecally (IT) through a lumbar puncture (LP) in a single subject with confirmed pathogenic mutations in the AP4M1 gene and clinical signs/symptoms of SPG50 disease.

5.2. Investigational Product

MELPIDA vials will be formulated as a concentrated stock in phosphate-buffered saline (PBS) containing 5% D-sorbitol and 0.001% Poloxamer 188, and stored at ≤ -60˚C until the day of the administration. The solution will be thawed within 4 hours prior to administration and diluted to the appropriate final dosage concentration and volume using PBS with 5% D-sorbitol and 0.001% Poloxamer 188 (if necessary).

5.3. Packaging

MELPIDA is supplied as a 2 mL Daikyo CZ® vial containing 1.15 mL of a sterile clear solution. Each mL contains 1E14 vector genome-containing particles (vg) of MELPIDA in phosphate buffered saline (PBS) containing 5% sorbitol and 0.001% Poloxamer 188

5.4. Labeling and Storage

MELPIDA and diluent are labeled with the lot/batch number, individual vial number, contents, and manufacture date, along with a warning that they are for investigational use only. They will be stored at or below -80˚C in Room 11123D at the Hospital for Sick Children. This is a locked, certified and monitored freezer located within the Hospital for Sick Children, Research Pharmacy, where the drug product will be stored.

5.5. Dose and Route

The participant will have a spinal needle inserted percutaneously at the lumbar level into the intrathecal space of the spinal column (L4/L5 interspace). A volume of CSF approximately equal to the infusion volume is withdrawn from the lumbar thecal sac. With the patient in the

Trendelenberg position (head down), the vector solution is then infused at a rate of 1 mL per minute for a total of 10 mL for participants 4 years of age and older (see Table 1 for volume adjustments for younger participants). The participant will remain side-lying in the Trendelenburg position (head down) at 15 degrees for one (1) hour following administration, during which time the patient will be turned (from left to right side/right to left side) every 15 minutes. Dosing volumes will be calculated per Table 1, depending on final vector product concentration. The procedure will be performed in a procedure unitwith an anesthesiologist or qualified physician present to administer sedation as needed. Participant will stay in the Pediatric Intensive Care Unit (PICU) overnight. As with the NIH GAN study, an immune suppression regimen will be utilized. Prophylactic enteral prednisone or prednisolone and sirolimuswill be administered to participant. Additional immunosuppression with tacrolimus will be administered as defined in the immune modulation protocol (Section 6.10).

Table 1 Dose Extrapolation Based on Age and Brain Size

Age (years)	Brain Volume (approx. cm3)	Infusion volume (mL)	Total IT High Dose (E14 vg)
4+	1312	10	10
3	1180	9	9
2	1080	8.2	8.2
1	955	7.3	7.3
0.5	525	4	4
Newborn	400	3	3

5.5.1. Dose Selection Rationale

5.5.1.1. Justification of clinical study dose

Nonclinical studies have evaluated toxicity, safety, tolerability, expression and biodistribution of MELPIDA in various models including normal (C57BL/6J) mice, an Ap4m1 knock-out (KO) mouse model of SPG50, Sprague Dawley (SD) rats and non-human primates (NHPs). In vitro studies have also been conducted in patient derived fibroblasts.

These studies support the following conclusions:

- Fibroblasts from 2x patients with SPG50 transduced with MELPIDA (using an AAV2 capsid) restored autophagy related 9A (ATG9A) trafficking and hence AP4 function, at multiplicity of infection (MOI)s of 1E2 to 1E5 vg/cell. This study showed a dose-dependent reduction in ATG9A staining at the TGN, AP4E1 localization to the TGN and unchanged staining for TGN46 with phenotypic rescue in up to 77% of fibroblasts and no associated toxicity.
- An in vivo efficacy study is ongoing in WT, heterozygous and homozygous Ap4m1 knock out (KO) mice dosed intrathecally at post-natal day (PND) 7 to 10 or PND 90 with no

treatment, vehicle, low (1.25E11), mid (2.5E11) or high (5E11) doses of MELPIDA and assessed for potential phenotypic rescue. Interim results demonstrated that MELPIDA increased hAP4M1opt mRNA in all brain regions at 3 weeks post dosing, and improved impaired behaviors, induced minimal immune responses, and did not lead to elevation of serum markers of toxicity at 5 and 8 months post dosing.

- An in vivo 12-month non-GLP toxicology study was carried out in WT C57BL/6J mice dosed intrathecally at the age of 7 weeks with vehicle, low (1.25E11), or high (5E11) doses of MELPIDA. Results demonstrated that MELPIDA was generally safe and well tolerated. Dose-dependent hAP4M1opt mRNA expression was noted in all brain regions at 4 weeks post IT injection, with expression sustained up to at least 12 months post infusion, confirming that MELPIDA reached and achieved transgene expression at the targeted site of action. There were no effects on body weight, hematology or clinical signs; minimal effects on clinical chemistry were noted. Several male animals were found to have hepatocellular adenoma's which are expected in these mice as they age (up to 51% in males aged 9 to 15m).
- An in vivo 3-month GLP toxicology and biodistribution study in WT Sprague Dawley (SD) rats dosed intrathecally at the age of 7 weeks with 0 (vehicle), 0.36E12, 1.1E12, or 3.3E12 vg/rat of MELPIDA was well tolerated. Findings were limited to neurobehavioral effects such as increased excitability and activity and decreases in body weight at 3.3E12 vg, and microscopic findings in the lumbar dorsal nerve roots, lumbar dorsal root ganglion, cauda equina in the injection site, and peripheral nerves (sciatic/tibial nerves). Due to the nature of the neuronal degeneration noted in the lumbar dorsal root ganglion at ≥ 1.1E12 vg and the absence of recovery in this finding, it was considered adverse. Based on these results, the no-observed-adverse-effect level (NOAEL) was considered to be 3.6E11 vg.
- An in vivo 3-month non-GLP toxicology and biodistribution study is ongoing in WT Cynomolgus monkeys (Macaca fascicularis) dosed intrathecally at the age of 2 to 4 years with 0 (vehicle), 8.4E13, or 1.68E14 vg/MELPIDA. Preliminary results indicate MELPIDA was well tolerated with no animals needing to be euthanized. There were no effects on body weight, weight gains, or appetite. There were no MELPIDA-related clinical observations. Clinical observations including, but not limited to, hunched posture, erected fur, tremors, decreased muscle tone, and decrease activity, were considered procedure-related and not due to the administration of MELPIDA due to their low incidence, transient or sporadic nature, or similar incidence in the control group. MELPIDA-related neurological changes were noted on Day 94 for Male No. 3201 and consisted of abnormal general attitude and motor function (slight tremors in the hindlimbs) and decreased proprioceptive positioning in the left hindlimb. There was no irritation noted at the puncture site. Intrathecal administration of MELPIDA at 1.68E14 vg resulted in a slight decrease in mean sural nerve conduction velocity of 11% on Day 45, relative to the control group, accompanied by a decrease in mean response amplitude of 27%. Male No. 3201 exhibited the most significant change with nerve conduction velocity of 44 m/sec compared to a mean of 52 m/sec in control animals. On Days 77 and 92, a significant decrease in mean nerve conduction velocity of 26% and 28%, respectively, was noted at 1.68E14 vg. The changes in conduction velocity were accompanied by associated decreases in mean sural nerve response amplitude which was reduced by 69% on Day 77 and by 74% on Day 92. Male No. 3201 was the most impacted, exhibiting the most pronounced changes with nerve conduction velocity of 34 m/sec (39%

change relative to mean control value) on Day 77 and 32 m/sec (36%) on Day 92 compared to 52-53 m/sec in control animals. The most pronounced decrease in response amplitude was also noted in Male No. 3201, particularly on Day 92, during which an amplitude of 1.2 µV was noted compared to a mean amplitude of 10.2 µV in control animals. No changes in peroneal nerve conduction velocity or amplitude and no changes in the onset latency of the cauda equina were noted up to Day 92. In addition, there were no changes in conduction velocity or amplitude for the sural nerve at 8.4E13 vg.

The findings from these various nonclinical studies provide the proof of concept to support the potential of benefit of MELPIDA to patients with SGP50.

Previous toxicology studies in rodents and large animals have indicated that the potential side effects emerging from the AAV9 capsids were minimal and manageable (Gougeon et al, 2021). Clinical experience for intrathecal AAV9 administration is emerging from several active human trials using AAV9–mediated gene replacement for treating CNS disorders initiated starting in 2015 (GAN, NCT02362438; CLN3, NCT03770572; CLN6, NCT02725580; MPS I, NCT03580083; SMA, NCT03461289, CLN7, NCT04737460, GM2, NCT04798235), with no serious safety concerns publicly disclosed from any of those trials. The primary anticipated complications from intrathecal AAV administration are likely to be anti-capsid immune responses, which appear to be manageable with transient immunosuppressive regimens (Gougeon et al, 2021; Ramsingh et al, 2018). Approximately 47% of humans are seropositive for AAV9, and naturally-occurring AAV9 is not known to cause any human disease (Boutin et al, 2010).

Vector distribution of MELPIDA after a single intrathecal administration measured in two studies was consistent with expected AAV9 biodistribution.

Tissue transduction and expression of the biologically active gene product in vivo were demonstrated in both WT rats, and WT and Ap4m1 KO mice. Efficacy endpoints were measured only in the Ap4m1 KO mouse model, which demonstrated a dose response in expression of hAP4M1 mRNA across both sexes, with a dose of 5E11 vg providing maximal and near normalization of behavioral tests and a lower dose of 2.5E11 providing significant (albeit lower) benefit to the mice. Of note, the lowest dose of 1.25E11 vg in mice did not provide a clear behavioral benefit, indicating a minimally effective dose of 2.5E11 vg in mice.

Safety data was gathered in rodents and NHPs. All studies indicated MELPIDA was generally safe and well tolerated at all doses with some neurobehavioral effects such as increased excitability and activity and decreases in body weight in the rat GLP study at 12 weeks post dose at the highest dose of 3.3E12 vg, which corresponds to a human dose of 1.8E15 vg. Other toxicities of note included neuronal degeneration in the lumbar dorsal root ganglion at doses of ≥ 1.1E12 vg with no recovery. The NOAEL was considered to be 3.6E11 vg corresponding to a human dose of 2.0E14 vg. In the NHP non-GLP study, NCV was reduced in the sural nerve in one animal at the 1.68E14 vg dose, corresponding to a human dose of 2E15 vg.

Safety concerns in humans are (i) possible immunological issues and (ii) the potential for DRG toxicity. The immunological issues relate to the possibility of a cytotoxic lymphocyte response against an expressed foreign antigen and the likelihood of a deleterious immune response to the

high intrathecal load of AAV9 capsid. The anti-AAV9 response is theoretical, but dose-responsive pleocytosis has been reported (Gougeon et al, 2021; Abstract #637 by Bharucha-Goebel et al, 2019 at American Society of Gene and Cell Therapy) that was not associated with clinical symptoms. Pleocytosis was managed with an extended steroid regimen and justifies adding additional transient T cell suppression strategies (Chu et al, 2021). These findings support the incorporation of the immune management protocol proposed with MELPIDA treatment in the clinic.

The toxicity noted within the dorsal root ganglia (DRG) in NHPs was a histological finding without any clinical/functional correlate (Hinderer et al, 2018; Hordeaux et al, 2020a). It was noted with MELPIDA in the rat GLP study (CRL-5550008) and presumed in the non-GLP NHP study due to the slowing of NCV in the sural nerve at Day 92 after the highest dose of 1.68E14 vg. However, the clinical significance of this finding in humans remains unknown at the present time.

The proposed dose for this N of 1 patient is 1E15 vg in 10 mL (as the subject will be 4 yo when dosed). When considering the CSF volume in the various species, this corresponds to a dose equivalent of 7.0E12 vg per mL of CSF in humans, mice, rats and NHPs. The comparative absolute vg dose across species is 1E15 vg in humans, 8.4E13 vg in NHPs, 1.8E12 vg in rats, and 2.4E11 vg in mice (Table 15; Figure 22). The pharmacology studies in mice predict a benefit to patients at this dose, considering a dose of 2.5E11 vg in mice provided a clear behavioral benefit with a 5E11 vg dose providing a greater benefit. Of note, 1.25E11 vg (equivalent to 5E14 vg in a human) did not provide a clear behavioral benefit, justifying 1E15 vg as the minimally effective human dose.

Table 1 Relationship between preclinical study doses and proposed human intrathecal dose

Species	Low dose (vg)	Low dose per CSF (vg/mL)	Mid dose (vg)	Mid dose per CSF (vg/mL)	Upper dose (vg)	Upper dose per CSF (vg/mL)	CSF Volume (mL)	HED of NOAEL (vg per mL CSF)	Intended human dose (vg)	Intended human dose (vg/mL)	Safety margin (vg/mL)
Mouse	1.3E11	3.6E12	2.5E11	7.1E12	5.0E11	1.4E13**	0.035	1.4E13	1E15	6.6E12	2.12x**
Rat	3.6E11	1.4E12	1.1E12*	4.4E12*	3.3E12	1.3E13	0.25	4.4E12			0.6x*
NHP	8.4E13*	7.0E12*	-	-	1.7E14	1.4E13	12	7E12			1.1x*
Human ≥4 y							140				

HED, human equivalent dose; CSF Volumes taken from the following references: Morgan et al, 2004; Sullivan et al, 1979; Pardridge, 2011; Pardridge, 1991.
*NOAEL in rat had minimal microscopic findings in lumbar dorsal nerve roots (note NHP study is still preliminary)
**based on upper mouse dose per CSF volume (vg/mL)

Figure 2 Summary of nonclinical findings to support clinical dosing

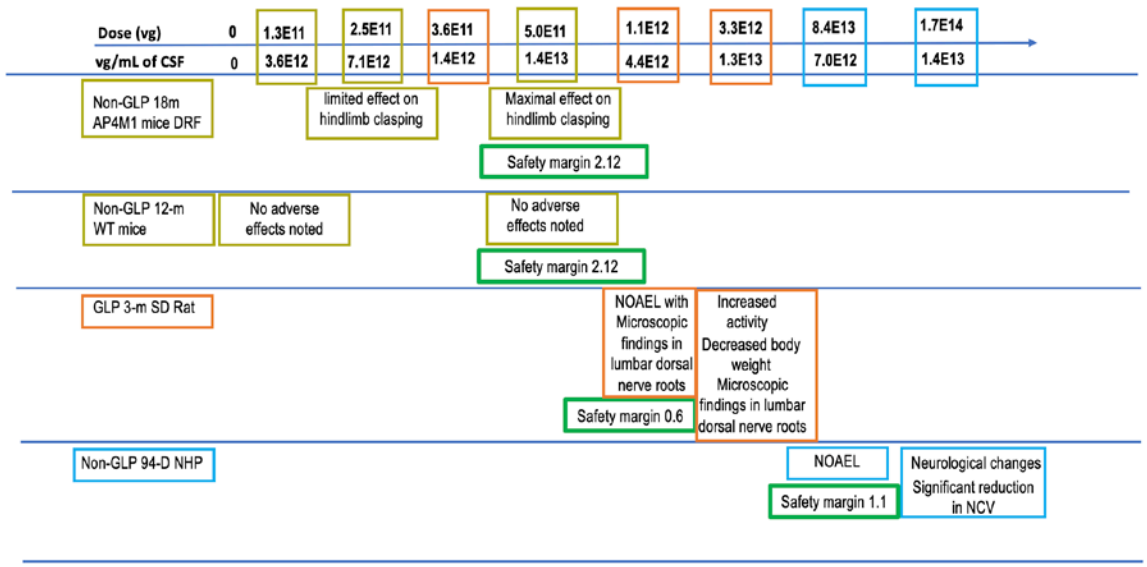

A feature of some SPG50 patients is microcephaly. Since dosing extrapolation across species is based on CSF or brain volume, this raises a possible concern that patients with microcephaly may receive a higher relative dose, since dosing extrapolations assume a patient with normal CSF and brain volume. According to Peterson et al (2021) and Centers for Disease Control growth charts, a patient with microcephaly (3rd percentile) would have a brain volume approximately 6% lower than an average person. A <10% correction factor for dosing would not be expected to have a strong impact on either safety or efficacy. Conversely, the added trial complexity and increased sedation risk to do a detailed volumetric MRI does not seem justified to impose such a corrective dosing factor individualized for each pediatric patient, especially those younger than 4 y. Considering this, the proposed plan is for any patient ≥4 years old to receive the same 1E15 vg dose in 10 mL.

In terms of safety, studies in mice up to 5E11 vg (equivalent to 2.0E15 vg human dose) were tolerated well to one year post-injection, with no significant drug-related effects on survival, body weight, body condition, blood chemistry, or histopathology. Studies in rats found increased activity, reduced weight, and histopathological findings at 3.3E12 vg (equivalent to 1.8E15 vg in humans), whereas lower doses were better tolerated with only sporadic minimal to mild histopathological findings. Studies in NHPs found reduced sensory nerve conduction at 1.7E14 vg (equivalent to 2E15 vg in humans), but otherwise showed minimal in-life adverse effects, and the lower dose of 8.4E13 vg (equivalent to 9.7E14 vg in humans) was tolerated well. Overall, the toxicology studies across 3 species provide safety data up to an approximately 2-fold overdose in the human equivalent dose (HED). While the preclinical toxicology studies predict the possibility of dorsal root ganglion specific pathology at the human 1E15 vg dose, this HED was not associated with adverse clinical findings in the animals. Considering MELPIDA as a one-time treatment for this severe neurodegenerative condition for this single patient without an

option to redose, a proposed dose of 1E15 vg in this single patient should maximize benefit with acceptable risks. Preclinical pharmacology data in the SPG50 mouse model did not clearly support a benefit at any lower dose.

Based on studies in rodents, there is some debated evidence of increased cancer risk associated with AAV vectors (Bolt et al, 2020; Bell et al, 2006; Rosas et al, 2012; Donsante et al, 2007). One large-scale study in mice found no evidence for tumorigenesis following AAV administration (Bell et al, 2005), despite other studies having found evidence for limited AAV integration (Chandler et al, 2015, 2016). While clonal integration of wildtype AAV2 has been detected in patient hepatocellular carcinomas (Nault et al, 2015), integration and increased cancer risk from rAAVs has not been identified in a clinical setting, and it is considered a very low risk for the proposed clinical trial. Moreover, our vector utilizes a relatively weak promoter, minimizing the risk of transactivation (overexpression) of oncogenes if the vector genome integrates nearby.

In summary, AAV9-based AP4M1 gene therapy has promise to treat SPG50 disease, but as with any other new modality, it has some development challenges. Preclinical safety studies in mice, rats, and NHPs demonstrate a favorable safety profile of MELPIDA, at up to twice the proposed human dose of 1E15 vg (doses extrapolated across species by CSF volume, Table 15). Pharmacology studies in the AP4M1 KO mouse model demonstrated a dose-dependent benefit of MELPIDA, with the greatest benefit seen at a dose of 5E11 vg in mice (scaled to approximately 1E15 to 2E15 vg in humans by CSF volume). Importantly, the safety concerns noted in the toxicology studies are balanced against the severe unmet medical need of this patient population and the potential benefit of intrathecal administration of MELPIDA.

6. SCHEDULE OF EVENTS

6.1. Screening:

The participant's parents/legal guardians will provide written informed consent for their child. The informed consent process can take place remotely via video call; the study and the risks/ benefits to study participation will be discussed in detail. If consent is obtained virtually via video conference, the family will be emailed a copy of the consent form to sign and return electronically. No study procedures will occur prior to consent/assent.

Between 28 and 8 days before potential dosing, the participant will be screened in-person at the Hospital for Sick Children and inclusion/exclusion criteria assessed. If the requirements (all of the inclusion criteria and none of the exclusion criteria) are met, the participant will be enrolled. The PI will also confirm that local care can supply adequate support to the subject in between visits.

At the screening visit, confirmation of genetic diagnosis, medical history, a review of concomitant medications, and the following clinical evaluations will be performed:

- A complete physical exam

- Vital signs – including heart rate, respiration rate, blood pressure, temperature, and oxygen saturation
- Height and weight
- EKG – a 15-lead electrocardiogram will record the participant's electrical heart rhythm
- A neurologic exam to include (but not be limited to) the testing of cranial nerves, muscle bulk/tone andstrength, sensation, cerebellar function, involuntary movements, myotatic reflexes, toe sign, gait, and stance.
- Liver ultrasound
- Blood and urinalysis
    - safety labs - Complete blood count (CBC) with differential, coagulation (INR, PT, PTT), ESR, CRP, Na, K, Cl, Ca, CO_2, BUN, Cr, glucose, ALT, AST, total bilirubins, direct bilirubins, ALP, GGT, serum total protein, cardiac safety panel (Tn, ProBNP, CK-MB), and urinalysis
    - Screening labs - Specifically for screening, the following will be assessed: HIV Ab, Hepatitis A Ab, Hepatitis B Surface Ab, Hepatitis Surface Ag, Hepatitis C Core Ab, PPD skin test for TB, HTLV1
- Spasticity assessments – the Modified Ashworth and Tardieu scales will be administered by a physical therapist to assess spasticity

The following exploratory assessments will also be performed at screening:

- AAV9 Antibody titers
- ELISPOT on whole blood for T cell response to AAV9 and A4PM1
- Bayley Scales of Infant and Toddler Development 4th edition (Growth Scale Value) (Fine Motor & Activities of Daily Living)
- Vineland Adaptive Behaviour Scale (Inter-personal Domain, Fine Motor Domain, Personal Domain)
- Log Book of Seizures – starting at the screening visit, the number and duration of seizures will be recorded daily
- Log Book Number of Falls – starting at the screening visit, the number of falls will be recorded daily
- Clinical Global Impression of Overall Change by Physician (CGI)
- Nerve conduction studies

6.2. Enrollment and pre-dosing schedule:

Following the Screening study visit, if the participant is eligible for enrollment, he will begin the immunosuppression regimen. Starting 7 days before dosing, the participant will begin Sirolimus loading at 1mg/m^2 every 4 hours on days -7, -6, and -5. On days, -4, -3, -2, and -1, the sirolimus dose will be lowered to 0.5 mg/m^2 divided into twice a day dosing, with the goal level being 4 to 8 ng/ml.
The day before dosing, the following procedures will be done:

- Physical exam
- Vital signs
- Recording of adverse events and concomitant medications
- Main efficacy outcome measures (Ashworth and Tardieu scales)
- Exploratory measures (Vineland, Bayley, log books of seizures and falls, CGI)
- Safety laboratories

6.3. Dosing day:

The following will be done on the day of dosing:

1) Exploratory labs: addition of Elispot and serum cytokines
2) Sirolimus dosing will be 0.5 mg/m^2 divided in twice a day dosing
3) Administration of additional immunosuppressants and analgesic:
    a. Methyl Prednisolone (IV) - 10 mg/kg to a maximum single dose 500 mg, infused over 30 minutes
    b. Acetaminophen - 15 mg/kg dose (maximum 650 mg/ dose)
    c. Diphenhydramine - 0.5 mg/kg/dose (maximum 50 mg/dose)
4) Lumbar puncture/Intrathecal administration of MELPIDA under anesthesia
    a. Removal of spinal fluid to equal volume of MELPIDA dose
    b. Cerebral-spinal fluid analysis, including cell count, differential, protein, glucose, gram stain, culture.
    c. Administration of MELPIDA (see below)
5) Collection of viral shedding samples (urine, saliva, stool) post dosing

6.3.1. Administration of Study intervention (MELPIDA):

A Pajunk atraumatic Sprotte needle (part number 321151-31A) will be inserted percutaneously at the lumbar level into the intrathecal space of the spinal column. Spinal needle placement will be

confirmed using fluoroscopic intraoperative imaging (CArm-) scanner at the chosen injection site prior to and after vector administration.

A volume of CSF approximately equal to the infusion volume (10 ml) will be withdrawn from the lumbar thecal sac. The MELPIDA vector solution will be loaded into a 20 mL BD syringe, connected to the needle with 60 inch mini volume IV extension tubing and a Braun 4-way stopcock. The vector solution is then infused at a rate of 1 mL per minute, using a CareFusion Alaris 8110 syringe pump or similar pump able to deliver at a rate of 1 mL/min.

6.3.2. Anesthesia Safety

The participant will undergo a comprehensive pre-anesthesia evaluation prior to dosing (within two weeks of dosing). Physiologic monitoring in accordance with the standards set by the American Society of Anesthesiologists will be utilized while they are receiving analgesia/anesthesia and until they have fully recovered from its effects. Active warming devices will be used during anesthesia as needed since patients are prone to hypothermia during the anesthesia.

6.3.3. Post-Procedure Recovery

After the procedure, if not conducted in the Pediatric Intensive Care Unit (PICU), the participant will be transported to a Post Anesthesia Care Unit (PACU) or (PICU) with continuous pulse oximetry monitoring and oxygen if needed by bag/mask/nasal canula or blow-by. Vital signs including heart rate, respiratory rate, blood pressure, and pulse oximetry will be monitored every 15 minutes for the first 2 hours post infusion, every 30 minutes during the third and fourth hour post infusion, then hourly for 4 hours,and finally every 4 hours until discharge. In the event that abnormalities are detected, appropriate medical intervention will occur, including the possibility of extending the hospitalization and/or subsequent testing. If low oxygen saturation is observed (< 93%), the patient will be evaluated and treated as clinically indicated. This may include, but are not limited to, assessments on physical examination, initiating of supplemental oxygen therapy, measurement of blood oxygen levels by ABG (arterial blood gas), Chest X-ray, CT imaging of the chest, PFTs (pulmonary function tests),or aerosol therapy. In the unlikely event that a severe allergic reaction should occur, the medical and nursing staff will follow the anaphylaxis guidelines.

6.4. Day 2

Vital signs, physical exam, neurologic exam, safety labs and a review of concomitant medications and any adverse events will be assessed prior to discharge. The first dose of tacrolimus at 0.1 mg/kg/day divided into twice daily dosing (goal level: 4-8 ng/mL) The first dose of prednisolone at 1 mg/kg/day will also be given. When the attending physician determines that the participant is stable, then he may be discharged. Immunosuppressive medications (prednisolone, tacrolimus and sirolimus) will be continued daily starting from Day 2.

6.5. Days 7, 14, 21, 28 (+/- 2 days)

The participant will return to SickKids on Day 7, 14, 21 and 28. Vitals, safety labs, brief physical exam, viral shedding samples, concomitant medications and adverse events will be collected. On Day 7 and 21 exploratory labs will also be done. Immunosuppression with prednisolone, tacrolimus and sirolimus will continue daily. Nerve conduction studies will be performed at 28 days.

6.6. Months 3, 6, 9 and 12 (+/- 14 days)

The participant will return to SickKids to repeat the majority of the study procedures (see Schedule of events), including all secondary outcome measures. Brain MRI and lumbar puncture with CSF analysis will be performed at 3, 6, 9, and 12 months (via one coordinated anesthesia, with MRI first). A liver ultrasound will be conducted at 6 and 12 months. Nerve conduction studies will be performed at 3, 6 and 12 months. If at month 3, there is no evidence of inflammation, prednisolone tapering may begin at month 4 (see Immune Modulation Protocol Section 6.10). Tacrolimus and sirolimus dosing will continue without tapering. If there is evidence of inflammation at Month 3, prednisolone tapering will not commence until after the next examination at Month 6. When there is no evidence of inflammation, then the schedule for tapering may commence. If signs of inflammation continue to be present at 6 months, immunosuppressive medicines will be continued, and MRI and LP will be repeated at 9 months to again assess.

6.7. Months 18, 24, 36, 48, 60 (+/- 14 days)

The month 18 study visit at SickKids will primarily be for safety (physical exam, vitals, height and weight, safety and exploratory labs) and efficacy assessments (spasticity, Bayley IV, and Vineland). Immunosuppression should be in the tapering phase for sirolimus and tacrolimus.

The participant/parents/guardians will be encouraged to contact the investigator for any suspected adverse event reporting between visits. Unscheduled visits may occur if the PI determines that they are necessary to assess safety, repeat labs, etc.

A complete schedule of events is found in Table 3.

Table 2 Schedule of Events

Visit/ Screen¥§ Day	-28 to -8	-7 to -1	-1	1	2	7	14	21	28	3 Month	6 Month	9 Month	12 Month	18 Month	24 Month	36 Month	48 Month	60 Month
Informed Consent	X																	
Genetic confirmation	X																	
Medical History	X																	
Vitals	X		X	X	X	X	X	X	X	X	X	X	X	X	X	X	X	X
Height and Weight	X			X						X	X	X	X	X	X	X	X	X
Physical Exam	X		X	X	X	X	X	X	X	X	X	X	X	X	X	X	X	X
Concomitant Medications §	X		X		X				X	X	X	X	X	X	X	X	X	X
Adverse Events §			X		X	X	X	X	X	X	X	X	X	X	X	X	X	X
Screen Labs*	X																	
Exploratory Labs****			X			X		X		X	X	X	X	X	X	X	X	X
Safety Labs**	X		X		X	X	X	X	X	X	X	X	X	X	X	X	X	X
MELPIDA Dosing				X														
PICU admit for dosing				X	X													
EKG	X			X						X	X	X	X		X	X	X	X
Neurologic Exam	X								X	X	X	X	X		X			
Lumbar Puncture				X						X	X	X	X		X			
CSF Analysis***				X						X	X	X	X		X	X	X	X
MRI	X****									X+	X	X	X		X		X	X
Ultrasound (Liver)	X										X		X		X	X	X	X
NCS	X							X		X	X		X		X	X	X	X
AAV NAb Titers	X		X		X	X	X	X		X	X		X		X	X	X	X
Anti A4PM1											X	X	X	X	X			
Viral shedding				X		X	X	X	X	X	X	X	X					
Immunosuppressants:																		
Prednisolone [10]					X	X	X	X	X	X [11]	X	X	X					
Acetaminophen [5]				X														
Diphenhydramine [6]				X														
Methylprednisolone (IV) [7]				X														
Tacrolimus (rapamycin) [8]					X	X	X	X	X	X	X [9]	X	X	X				
Sirolimus		X[1]	X[1]	X[2]	X[3]	X	X	X	X	X	X	X	X[4]	X				
Spasticity Assessments [13]	X		X							X	X	X	X	X	X	X	X	X
Bayley Scales of Infant and Toddler Development ed IV	X		X							X	X	X	X	X	X	X	X	X

Vineland Adaptive Behaviour Scale	X	X					X	X	X	X	X	X	X	X	X
Log book of seizures [14]	X	X	X	X	X	X	X	X	X	X	X	X	X	X	X
Log book of falls [14]	X	X	X	X	X	X	X	X	X	X	X	X	X	X	X

¥Some visits are in-patient admissions and may last for more than the day of admittance.
§Between study visits there will be biweekly phone calls to assess con meds and AEs.
*Screening labs include HIV Ab, Hepatitis A Ab, Hepatitis B Surface Ab, Hepatitis Surface Ag, Hepatitis C Core Ab, PPD skin test for TB, HTLV1
**Safety labs include CBC With Differential, ESR, CRP, LFT, HCT, MCV, Na, K, Cl, CO_2, BUN, Cr, glucose, ALT, AST, Total biilrubins, Direct.bilirubins, ALP, GGT, Ca, INR, PT, PTT, Urinalysis, EKG, Cardiac Safety (Tn, ProBNP, CK-MB), Sirolimus Levels, Tacrolimus levels, Lipid Profile
***CSF Analysis includes cell count, differential, protein, glucose, gram stain, culture, oligoclonal bands, cytokine analysis.
****Exploratory Tests = AAV9 NAb, Serum Cytokine Analysis & EliSPOT
*****May use previous baseline MRI if within 2 years of dosing
X+ = MRI Brain & Spine With contrasts (at Baseline & 3M only)

1- Sirolimus load 1 mg/m^2 every 4h for 3 doses (days -7, -6, -5), then 0.5 mg/m^2/day divided in twice a day dosing (goal level 4-8 ng/ml) (days -4, -3, -2, -1)
2- Sirolimus maintenance dose 0.5 mg/m^2 divided in twice a day dosing on day 1
3- Sirolimus maintenance dose 0.5 mg/m^2 divided in twice a day dosing DAILY from day 2 to Month 12
4- Sirolimus taper may begin starting at Month 12. If any evidence of ongoing inflammation in any exam at Month 12, continue with 0.5 mg/m^2 divided in twice a day doses DAILY, and do not begin taper until results of next clinical evaluation of inflammation and reassess.
5- Acetaminophen 15 mg/kg dose (max 650 mg/ dose)
6- Diphenhydramine 0.5 mg/kg/dose (max 50 mg/dose)
7- Methylprednisolone (IV) 10 mg/kg to a max single dose 500 mg, infused over 30 minutes
8- Tacrolimus 0.1 mg/kg twice DAILY (goal level 4-8 ng/ml) from day 2 to month 6
9- Tacrolimus - Based on clinical results of LP, MRIs at Month 6, tacrolimus taper may begin at Month 6 (Week 24). If any evidence of ongoing inflammation in any exam at Month 6, continue with 0.1 mg/kg/day DAILY; do not begin taper until results of next clinical evaluation of inflammation and reassess
10- Prednisone 1 mg/kg/day DAILY until month 4
11- Prednisone - Based on clinical results of LP, MRIs and ophthalmologic exam at Month 3, prednisone taper may begin at Month 4 (Week 16). If any evidence of ongoing inflammation in any exam at Month 3, continue with 1 mg/kg/day DAILY; do not begin taper until results of next clinical evaluation of inflammation and reassess
12- If inflammation evident at month 6, repeat MRI, LP and CSF analysis at month 9
13- Modified Ashworth and Tardieu scales to assess spasticity
14- Starting at screening, the number of falls and seizures will be recorded daily
15- Blood draw will take into account body weight, daily and 30 day reference maximums

6.8. Specific Study Procedures

6.8.1. Nerve conduction study (NCS)

The participant will undergo nerve conduction studies to evaluate for peripheral nerve injury. A baseline study will be performed within 28 days of dosing. Standard methodology, as used in routine clinical care, will be used. At least two sensory and one motor nerve in the upper and lower extremity will be sampled. Nerves to be tested (Sensory action potentials (SAP); Sural, peroneal superficialis, median and ulnar. Compound Muscle action Potentials (CMAP); Tibial, Median) Studies should be always done on the same side each time

Sensory action potentials (SAP) will be recorded with an orthodromic procedure for median, and ulnar nerves, antidromically in the superficial peroneal and sural nerves. Motor distal latencies, compound muscle action potential and minimal F-wave latencies will be recorded for median, and tibial nerves. No EMG will be done unless we observe an axonal neuropathy.

NCVs will be done at baseline, day 21, 3 month, 6 month, 12 month,24 monh, 48 month and 60 month after dosing.

6.8.2. Magnetic Resonance Imaging of Brain (MRI)

Brain MRI (with and without contrast) will be done at baseline and at 3 month intervals. Standard imaging sequences will be obtained (T1, T2, FLAIR, etc). MRI studies will be done at The Hospital for Sick Children. The primary role for brain MRI in the study is to evaluate for signs of inflammation and inflammatory change associated with the study drug. Of note, patients with SPG50 typically have structural brain abnormalities. On MRI, the following changes have been observed in SPG50 patients: (1) thin splenium of the corpus callosum, (2) absent or thin anterior commissure, (3) characteristic signal abnormalities of the forceps minor (“ears of the grizzly sign”), and (4) periventricular white matter. All MR imaging will be supervised by an attending pediatric neuroradiologist to ensure acquisition of complete, high quality scans.

6.8.3. Liver Ultrasound

A liver ultrasound will be conducted at pre-dose, Months 6 and 12 and then yearly. Liver imaging studies will be done at the Hospital for Sick Children. The primary role for the liver ultrasound to is monitor signs of liver toxicity associated with the study drug. All ultrasound imaging will be supervised by an attending pediatric neuroradiologist to ensure acquisition of complete, high quality scans.

6.8.4. Lumbar puncture

The participant will undergo one lumbar puncture via interventional radiology guidance. The participant will undergo additional post-dosing lumbar punctures as performed without IR

guidance by the study PI. For all LPs, a 21 gauge standard LP needle will be used. The area around lumbar 4/5 will be sterilely addressed. EMLA will be applied for local anesthesia. The needle will be inserted into the inter thecal space between L4/L5. An appropriate quantity of CSF will be removed for relevant laboratory studies. The needle will be removed, the site cleaned, and a sterile dressing will be applied.

6.9. Adverse Event and Concomitant Medication Monitoring

The participant’s caregivers are encouraged to contact the study team whenever an adverse event or change in medication occurs in a timely manner. Long term adverse event monitoring will include annual study visits at SickKids.

6.10. Immune Modulation Protocol

In previous gene therapy studies, antigen specific T-cell responses to the AAV9 vector have been reported (Harrison et al, 1977). This is an expected response between 2- and 12-weeks following gene transfer, even when administered IT. One possible consequence to such antigen specific T-cell responses is clearance of the transduced cells and loss of transgene expression.

To reduce the risk of the host immune response to the AAV9-based MELPIDA, an initial proposal for an immunosuppression regimen has been designed based on advice from investigators in the ongoing trial of AAV9 gene transfer to CSF for giant axonal neuropathy (Clinicaltrials.gov # NCT02362438).

<u>1 Week Prior to Vector Administration:</u>
- Sirolimus load: 1 mg/m2 every 4 hours x 3 doses (load only given on one day)
- Starting the day after the sirolimus load, begin enteral daily dosing at 0.5 mg/m2/day, divided in twice per day dosing (goal level: 4-8 ng/mL)

<u>Day of Vector Administration (Day 1):</u>
- Acetaminophen (15 mg/kg/dose enteral; maximum 650 mg per dose)
- Diphenhydramine (0.5 mg/kg/dose enteral; maximum 50 mg/dose)
- IV methylprednisolone (10 mg/kg to a maximum single dose of 500 milligrams, infused over 30 minutes)

<u>Day after Vector Administration (Day 2):</u>
- Begin daily enteral prednisone/prednisolone at 1 mg/kg/day x 3 months
- Continue enteral daily sirolimus dosing at 0.5mg/m2/day, divided in twice per day dosing (goal level: 4-8 ng/mL)
- Tacrolimus at 0.1 mg/kg/day divided into twice daily dosing (goal level: 4-8 ng/mL)

<u>Maintenance:</u>
- Enteral prednisone/prednisolone at 1 mg/kg/day x 3 months, then taper according to schedule

- Sirolimus 0.5 mg/m2/day, divided in twice per day dosing. If there are signs or symptoms of transgene mediated CNS inflammation by examination, brain imaging, and/or laboratory testing, longer administration of immunomodulatory medications and possibly addition of other immunomodulatory agents may be required
- Tacrolimus at 0.1 mg/kg/day divided into twice daily dosing (goal level: 4-8 ng/mL); tacrolimus will be continued for 6 months and will begin taper by 7 months after gene transfer. The taper will be started if there are no signs or symptoms of transgene mediated CNS inflammation by examination, brain imaging, and/or laboratory testing, which if present may require longer administration of immunomodulatory medications

<u>Monitoring:</u>
- Weekly BP checks x 4 weeks
- Sirolimus troughs every 1 week x 4, then every 2 weeks x 4, then monthly once levels are stable within the desired range
- Tacrolimus troughs every 1 week x 4, then every 2 weeks x 4, then monthly once levels are stable within the desired range
- CBC with differential testing at every blood draw
- 8 AM cortisol level when participants are on 5 mg dose prednisone/prednisolone for 1 week
- Monthly fasting lipid profile while on immunomodulation and at PI's discretion

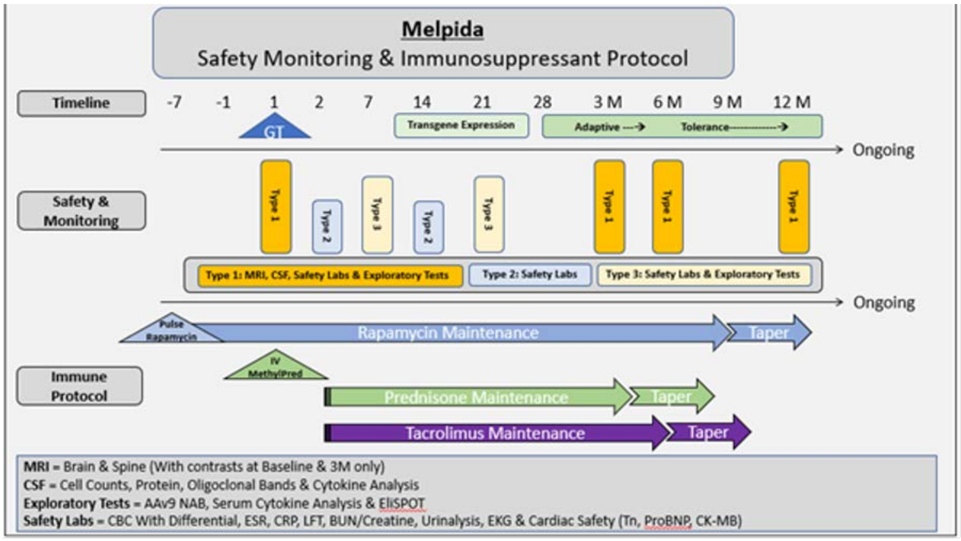

7. INCLUSION/EXCLUSION CRITERIA

7.1. Inclusion Criteria

- Age <5 years old

- Confirmed diagnosis of SPG50 disease by:
  - Genomic DNA mutation analysis demonstrating homozygous or compound heterozygous, pathogenic and/or potentially pathogenic variants in the AP4M1 gene
  - Clinical history or examination features consistent with SPG50 and that include neurologic dysfunction

- Parent/legal guardian willing to accompany the participant to all study visits and who will provide permission for their child's participation.

7.2. Exclusion Criteria

- Inability to participate in the clinical evaluation
- Presence of a concomitant medical condition that precludes lumbar puncture or use of anesthetics
- Bleeding disorder or any other medical condition or circumstance in which a lumbar puncture is contraindicated according to local institutional policy
- Inability to be safely sedated in the opinion of the clinical anesthesiologist
- Active infection based on clinical observations
- Concomitant illness or requirement for chronic drug treatment that in the opinion of the PI creates unnecessary risks for gene transfer
- Any item which would exclude the patient from being able to undergo MRI according to local institutional policy
- Any other situation that would exclude the patient from undergoing any other procedure required in this study
- The presence of significant non-SPG50 related CNS impairment or behavioral disturbances that would confound the scientific rigor or interpretation of results of the study
- Have received an investigational drug within 30 days prior to screening or plan to receive an investigational drug (other than gene therapy) during the study.
- Enrollment and participation in another interventional clinical trial
- Contraindication to MELPIDA or any of its ingredients
- Contraindication to any of the immune suppression medications used in this study
- Clinically significant abnormal laboratory values (GGT, ALT, and AST, or total bilirubin $> 2 \times$ ULN, creatinine ≥ 1.0 mg/dL, hemoglobin [Hgb] < 8 or > 18 g/dL; white blood cell [WBC] $> 20{,}000$ per cmm) prior to gene replacement therapy. Patients with an elevated bilirubin level that is unequivocally the result of neonatal jaundice shall not be excluded

7.3. Participant Withdrawal

The participant/parents/guardians will be consented prior to their enrollment in the study. They will be made aware that participation in the study is voluntary and they can withdraw at any time. Participants are free to withdraw from participation in the study at any time upon request. If possible, the study site should attempt to have the participant return for one last study visit (for t = 36 month study visit procedures).

Early withdrawal may also occur for any of the following reasons:
1. Protocol deviation (at the Investigator's discretion)
2. Investigator discretion
3. Study termination by Investigator
4. Lost to follow up
5. Participant enrollment in a different interventional study for SPG50

An investigator may discontinue or withdraw a participant from the study for the following reasons:
- If any clinical adverse event (AE), laboratory abnormality, or other medical condition or situation occurs such that continued participation in the study would not be in the best interest of the participant
- Disease progression which requires discontinuation of the study intervention
- If the participant meets an exclusion criterion (either newly developed or not previously recognized) that precludes further study participation

Follow-up Procedures following Withdrawal
For the safety of the participant, every effort will be made for a final evaluation (per t = 36 month study visit). This includes follow-up for any unresolved adverse events.

8. STATISTICAL ANALYSIS

Data collected from this study will be presented in aggregate. Rates of adverse events and serious adverse events will be reported by category. Given that this is an N of 1 trial, analytic statistics at a population level will not be possible. Descriptive statistics will be provided. Furthermore, rates of change in disability scores will beprovided, but the study is not powered to discern the effect size of the therapy.

8.1. Data Monitoring

8.1.1. General Plan

For this Phase I study of Gene Therapy the safety oversight will be focused not only on the initial treatment but also on an extended observation.

8.1.2. Monitoring Entity

Dr. James Dowling (SickKids Toronto) is the lead investigator and will be responsible for assuring ongoing safety monitoring of the trial. All medical decisions will be made in the best interest of the participant. Management of toxicities will be at the discretion of the lead investigator in consultation with experts in the field.

8.2. Plans for Assuring Participant Safety, Adverse Event Collection, and Reporting

Expected adverse events could be due to the infusion and are listed above. Other adverse events could be due to immunomodulatory drugs or to an inflammatory response to the AAV.

Maximum efforts will be undertaken to ensure the safety of study participant as per the requirements of HC and SickKids REB.

The primary and secondary endpoint assessment is identifying the safety and tolerability of intrathecal administration of MELPIDA. Monitoring for safety will be performed by recording and evaluating type and occurrences of Adverse Events (AEs), concomitant medication usage, and by conducting physical examinations, vital sign assessments, cardiovascular evaluations, and laboratory evaluations (chemistry, hematology, coagulation,immunology).

8.3. Definitions

8.3.1. Adverse Event

An Adverse Event (AE) is any untoward medical occurrence associated with the use of an intervention in a study participant, which does not necessarily have a causal relationship with the intervention. An AE can therefore be any unfavourable and unintended sign (including an abnormal laboratory finding), symptom or disease temporally associated with the use of the intervention, whether or not considered related to the investigational intervention.

Stable chronic conditions which are present prior to entry in the study and do not worsen are not considered AE. These pre-existing conditions will be documented in the participant's medical history.

These events will be reviewed by the PI and determined if they are clinically significant requiring adjustments to medications or interventions as per HC regulations. Reporting to HC and REB are outlined in Section 8.5, Section 8.6 and Section 8.7.

Clinically significant signs and symptoms or lab abnormalities will be recorded as an AE. This could include a laboratory result for which there is no intervention, but the abnormal value suggests a disease or organ toxicity. The PI will evaluate all AEs with respect to Seriousness, Severity (intensity or grade), and Causality (relationship to study agent and relationship to research) according to the following guidelines. All AEs will be classified in accordance with the

CTCAE v.5. AEs will be coded in accordance with the most current version of the MedDRA coding dictionary.

.

8.3.2. Classification of Adverse Events

Monitoring AEs requires that they be classified as to seriousness, expectedness, and potential relationship to the investigational product, all of which drive the reporting process.

8.3.2.1. Seriousness

A serious adverse event (SAE) is one that:

- Results in death,
- Is life-threatening (the participant was in immediate danger of death from the event as it occurred),
- Requires inpatient hospitalization or prolongation of existing hospitalization,
- Results in persistent or significant disability/incapacity, or
- Is a congenital anomaly/birth defect in the offspring of a participant.

All SAEs that occur after any patient has been enrolled, before vector dosing, during vector dosing, or up through the last study visit, whether or not they are related to the study, must be recorded on case report forms.

CTCAE v.5 provides a grading system that is used to categorize the severity of adverse events, as follows:

- Grade 1 Mild: transient, requires no special treatment or intervention, does not interfere with daily activities
- Grade 2 Moderate: alleviated with simple treatments, may limit daily activities
- Grade 3 Severe: requires therapeutic intervention and interrupts daily activities
- Grade 4 Life-threatening or disabling
- Grade 5 Death

An SAE, as defined above, encompasses CTCAE grades 4 and 5, and any Grade 3 event that requires or prolongs hospitalization, or that is disabling. Other SAEs that are considered Important Medical Events (IME) requiring medical judgement that need reporting is when the event does not fit the outcomes listed above, but the event mayjeopardize the patient and may require medical or surgical intervention (treatment) to prevent one of the other outcomes.

8.3.2.2. Expectedness

The purpose of reporting is to provide new, important information on serious reactions or events previously unobserved or undocumented. Therefore, all AEs will be evaluated as to the expectedness of its occurrence as follows:

- Unexpected: An unexpected AE or adverse drug reaction is one for which the nature or severity is not consistent with information in the protocol, consent form, or Investigator's brochure.
- Expected: While there remains limited data on the safety of AAV9 and AAV based gene therapy programs in general, some AEs have emerged in across different programs. Ones that have been noted in multiple individuals and in more than one patient include:
    a. Transient thrombocytopenia
    b. Transient transaminitis
- An AE is considered expected if it is known to be associated with any of the study procedures (i.e., blood draw sticks, LPs, etc)
- Expected adverse events <u>due to underlying disease</u> are listed below:
    - Worsening of spasticity
    - Worsening of ataxia
    - Worsening seizures
    - Progressive atrophy in brain noted in follow-up MRIs

8.3.2.3. Causality

Causality assessment is required in clinical investigations to help determine which events require expedited reporting. The PI must make the determination of relationship to the investigational product for each AE (Unrelated,Possibly Related, Probably Related, or Definitely Related). The PI should decide whether, in his/her medical judgment, there is a reasonable possibility that the event may have been caused by the investigational product. If no valid reason exists for suggesting a relationship, then the AE should be classified as "unrelated." If there is anyvalid reason, even if undetermined, for suspecting a possible causative relationship between the investigational product and the occurrence of the AE, then the AE should be considered "related." If the relationship between the AE/SAE and the investigational product is determined to be "possible" or "probable", the event will be considered to be related to the investigational product for the purposes of expedited regulatory reporting.

The following criteria will be used to determine causality:

- Unrelated: The event is clearly related to other factors, such as the participant's clinical state or non-study drugs or interventions.
- Possibly Related: The event follows a compatible temporal sequence from the time of administration of the study agent, but could have been produced by other factors such as the participant's clinical state or non-studydrugs or interventions.

- Probably Related: The event follows a reasonable temporal sequence from the time of study agent administration, and cannot be reasonably explained by other factors such as the participant's clinical state or non-study drugs or interventions.

8.4. Dose Limiting Toxicity

Dose limiting toxicity (DLT) is defined as any SAE or AE that is possibly, probably, or definitely related to the investigational product. This would include any AE Grade 3 or greater event, according to the CTCAE v.5; these classificationsare outlined below:

- Grade 1 Mild: transient, requires no special treatment or intervention, does not interfere with daily activities
- Grade 2 Moderate: alleviated with simple treatments, may limit daily activities
- Grade 3 Severe: requires therapeutic intervention and interrupts daily activities
- Grade 4 Life-threatening or disabling
- Grade 5 Death

Other Adverse Events

Other adverse events (OAEs) may be identified by the PI. Significant AEs of particular clinical importance, other than SAEs and those AEs leading to discontinuation of the participant from the study, will be classified as OAEs.

8.5. Reporting Procedures to the REB

The REB will have access to review of participant data during the course of the study through access to the participant's eCRFs. All SAEs will also be reported to the REB. Requests for additional data can be made by communicating the request to the PI.

8.6. Reporting Procedures to Health Canada

As the PI is the sponsor-Investigator, the PI will notify HC of potential serious risks, from clinical trials or any other source, as soon as possible. For fatal or life threatening events, the PI will report to HC with 7 days and when neither fatal or life threatening, they will report within 15 days, as per HC regulations.

Information that qualifies reporting:

- *Serious and unexpected suspected adverse reaction.* The PI will report any suspected adverse reaction thatis both serious and unexpected. The PI will report an adverse event as a suspected adverse reaction only if there is evidence to suggest a causal relationship between the drug and the adverse event, such as

- single occurrence of an event that is uncommon and known to be strongly associated with drug exposure.
- One or more occurrences of an event that is not commonly associated with drug exposure, but is otherwise uncommon in the population exposed to the drug.
- An aggregate analysis of specific events observed in a clinical trial (such as known consequences of the underlying disease or condition under investigation or other events that commonly occur in the study population independent of drug therapy) that indicates those events occur more frequentlyfollowing treatment.

- *Findings from other studies.* The PI will report any findings from epidemiological studies, pooled analysisof multiple studies, or clinical studies that suggest a significant risk in humans exposed to the drug.
- *Findings from animal or in vitro testing.* The PI will report any findings from animal or in vitro testing, whether or not conducted by the sponsor, that suggest a significant risk in humans exposed to the drug.
- *Increased rate of occurrence of serious suspected adverse reactions.* The sponsor must report anyclinically important increase in the rate of a serious suspected adverse reaction over that listed in the protocol or investigator brochure.
- *Submission of CTA safety reports.* The PI will submit each CTA safety report in a narrative format or in an electronic format that HC can process, review, and archive. Reports of overall findings or pooled analyses from published and unpublished in vitro, animal, epidemiological, or clinical studies must be submitted in a narrative format.
- *Unexpected fatal or life-threatening suspected adverse reaction reports.* The PI will also notify HC of any unexpected fatal or life-threatening suspected adverse reaction as soon as possible but in no case laterthan 7 calendar days after the PI's initial receipt of the information.

8.7. Reporting Procedures to the Research Ethics Board

Suspected unexpected serious adverse reactions (SUSARs) will be reported to the REB within 5 workingdays of discovery if they follow the following definition:

An event that meets ALL three (3) of the following criteria:

- Unexpected (in nature, severity, or frequency), AND
- Probably or definitely related to participation in research, AND
- Suggests the investigational product places subjects or others at a greater risk of harm than previously known or recognized.

All other research-related events and reports will be summarized at annual continuing review (AR) or notice of study closure, whichever comes first. That includes, but is not limited to:

- Noncompliance events (e.g., deviations)that do not meet the SickKids REB definition of either serious or continuing noncompliance
- AEs/SAEs that do notmeet ALL 3 SUSAR criteria
- Events/reports the sponsor wants submitted to the SickKids REB
- Other safety reports
- Monitoring/audit reports
- Any other new information since the last REB review

A Summary Report of AEs will be prepared by the PI annually and will be sent to the REB at continuing review. The Summary Report will contain the following information:

- A statement as to whether or not the frequency of AEs exceeded what was expected and indicated in the informed consent.
- A statement that if safety concerns are identified, they will be communicated promptly to the investigators.

8.8. Protocol Deviations and Continuing Review

Protocol deviations and unanticipated problems will be reported to the SickKids REB as per their reporting guidelines.

The following items will be reported to the SickKids REB in summary at the time of Continuing Review:

- Serious and non-serious unanticipated problems,
- Expected serious adverse events that are possibly, probably, or definitely related to the investigational product,
- Serious adverse events that are not related to the investigational product
- All adverse events, except expected AEs and death granted a waiver of reporting,
- Any trends or events which in the opinion of the investigator should be reported, and
- Any protocol-specific reporting requirements (as applicable).

8.9. Stopping Rules

Not applicable for this single participant study.

9. DATA COLLECTION

Data will be collected at specified time intervals as outlined in the protocol. Once the participant and/or parent/guardian has signed the informed consent/assent form, data can then be collected, including pertinent retrospective medical records per PI discretion.

Source data is all information, original records of clinical findings, observations, or other activities in a clinical trialnecessary for the reconstruction and evaluation of the trial. Source data are contained in source documents. Examples of these original documents, and data records include: hospital records, clinical and office charts, pathology reports, laboratory notes, memoranda, participants' diaries or evaluation checklists, pharmacy dispensingrecords, recorded data from automated instruments, copies or transcriptions certified after verification as being accurate and complete, microfiches, photographic negatives, microfilm or magnetic media, digitized imaging data,x-rays, participant files, and records kept at the pharmacy, at the laboratories, and at medico-technical departmentsinvolved in the clinical trial.

The study electronic case report forms (eCRF) is where all data collection will be inputted for the study. All data requested on the eCRF will be recorded by the clinical operations team consisting of the clinical research coordinators/managers and research nurses. The electronic data capture (EDC) platform used in this clinical trial will be REDCap. It supports regulatory trials to ensure Good Clinical Practice (GCP). All missing data must be explained, and it will have automatic data verification in place to ensure complete and accurate data is entered. REDCap will be accessed via a secure personalized login, thus allowing for role-appropriate access, and providing audit trails for data entry,exports, and reports. Trial data will be entered using the participant identification number. PHI will not be shared outside of REB approved entities.

9.1. Database Locks

For key deliverables requiring analysis of the trial data, an export of the entire database from REDCap will be performed at such periodic intervals in order to have a locked dataset from which all results will be generated. At trial end, a final lock and export will occur after all data queries are resolved and statistical analysis will be performed. The final trial results and publications will be prepared from this locked dataset.

9.2. Study Monitoring Plan

This study will be monitored according to the outline in the protocol. The PI will allocate adequate time for such monitoring activities. The data entered into REDCap will be reviewed and verified for accuracy by the Investigator.

SickKids Hospital Research Clinical Research Quality and Education (CRQE) will also provide trial monitoring. The ongoing data monitoring responsibilities are performed by the SickKids Hospital PI, Dr. Dowling, and the CRQE, to monitor the study progress and will function independently from the study team. The PI will also ensurethat SickKids Hospital REB and CRQE or other compliance/quality assurance reviewers are given access to all the above noted

study-related documents and study-related facilities and has adequate space to conduct monitoring visits. The PI will permit study-related monitoring, audits, and inspections by the government regulatory bodies such as the HC.

9.3. Quality Assurance of Data

Quality assurance (QA) processes are in place to ensure the data will be collected and entered into the EDC accurately and consistently.

10. INSTRUMENTS FOR THE ASSESSMENT OF DISEASE

10.1. Modified Ashworth Scale

Modified Ashworth Scale Instructions

General Information (derived Bohannon and Smith, 1987):

- Place the patient in a supine position
- If testing a muscle that primarily flexes a joint, place the joint in a maximally flexed position and move to a position of maximal extension over one second (count "one thousand one")
- If testing a muscle that primarily extends a joint, place the joint in a maximally extended position and move to a position of maximal flexion over one second (count "one thousand one")
- Score based on the classification below

Scoring (taken from Bohannon and Smith, 1987):

0	No increase in muscle tone
1	Slight increase in muscle tone, manifested by a catch and release or by minimal resistance at the end of the range of motion when the affected part(s) is moved in flexion or extension
1+	Slight increase in muscle tone, manifested by a catch, followed by minimal resistance throughout the remainder (less than half) of the ROM
2	More marked increase in muscle tone through most of the ROM, but affected part(s) easily moved
3	Considerable increase in muscle tone, passive movement difficult
4	Affected part(s) rigid in flexion or extension

10.2. Tardieu Scale

TARDIEU SCALE

This scale quantifies muscle spasticity by assessing the response of the muscle to stretch applied at specified velocities.

Grading is always performed at the same time of day, in a constant position of the body for a given limb. For each muscle group, reaction to stretch is rated at a specified stretch velocity with 2 parameters x and y.

Velocity to stretch (V)

**V1**	As slow as possible
**V2**	Speed of the limb segment falling
**V3**	As fast as possible (> natural drop)

V1 is used to measure the passive range of Motion. (PROM). Only V2 and V3 are used to rate spasticity

Quality of muscle reaction (X)

**0**	No resistance throughout passive movement
**1**	Slight resistance throughout, with no clear catch at a precise angle
**2**	Clear catch at a precise angle, followed by release
**3**	Fatigable clonus (<10secs) occurring at a precise angle
**4**	Unfatigable clonus (>10secs) occurring at a precise angle
5.	Joint Immobile

Angle of muscle reaction (Y)

Measure relative to the position of minimal stretch of the muscle (corresponding at angle)

Spasticity Angle

**R1**	Angle of catch seen at Velocity V2 or V3
**R2**	Full range of motion achieved when muscle is at rest and tested at V1 velocity

Boyd, Graham 1999

- A large difference between R1 & R2 values in the outer to middle range of normal m. length indicates a large dynamic component
- A small difference in the R1 & R2 measurement in the middle to inner range indicates predominantly fixed contracture

Testing Positions

Upper Limb

To be tested in a sitting position, elbow flexed by 90° at the recommended joint positions and velocities.

**Shoulder**	Horizontal Adductors	V3	
	Vertical Adductors	V3	
	Internal Rotators	V3	
**Elbow**	Flexors	V2	Shoulder adducted
	Extensors	V3	Shoulder abducted
	Pronators	V3	Shoulder adducted
	Supinators	V3	Shoulder adducted
**Wrist**	Flexors	V3	
	Extensors	V3	
	Fingers		Angle PII of digit III- MCP
	Palmar Interossei + FDS	V3	Wrist resting position

Lower Limb

To be tested in supine position, at recommended joint positions and velocities

**Hip**	Extensors	V3	Knee extended
	Adductors	V3	Knee extended
	External Rotators	V3	Knee flexed by 90
	Internal Rotators	V3	Knee flexed by 90
**Knee**	Extensors	V2	Hip flexed by 30
	Flexors	V3	Hip flexed
**Ankle**	Plantarflexors	V3	Knee flexed by 30

11. PRIVACY AND CONFIDENTIALITY

Participant confidentiality and privacy is strictly held in trust by the participating Investigators, their staff, and the Sponsor(s). This confidentiality is extended to cover testing of biological samples and relevant genetic tests in addition to the clinical information relating to participant. Therefore, the study protocol, documentation, data, and all other information generated will be held in strict confidence.

All research activities will be conducted in as private a setting as possible.

Any research information obtained about the patient in this study will be kept confidential. A patient will not be identified by name, only by unique study ID number. The patient's name or any identifying information will not appear in any reports published as a result of this study. All identifying information will be kept behind 2 security measures or as per equivalent institutional policy, under the supervision of the study/site PI and will not be transferred outside of the hospital.

The study monitor, auditor and other authorized representatives of the Sponsor, representatives of the Research Ethics Board (REB) or HC may inspect all documents and records required to be maintained by the Investigator, including but not limited to, medical records and pharmacy records for the participant in this study. The clinical study site will permit access to such records.

Study participant research data, which is for purposes of statistical analysis and scientific reporting, will be transmitted to and stored in REDcap. This will not include the participant's contact or identifying information. Rather, the participant and their research data will be identified by a unique study identification number. The study data entry and study management systems used by SickKids research staff will be secured and password protected. At the end of the study, all study databases will be de-identified and archived.

12. REFERENCES

Aguilar RC, Boehm M, Gorshkova I, et al. Signal-binding specificity of the mu4 subunit of the adaptor protein complex AP-4. J Biol Chem. 2001;276(16):13145-13152. doi:10.1074/jbc.M010591200

Bailey RM, Armao D, Nagabhushan Kalburgi S, Gray SJ. Development of Intrathecal AAV9 Gene Therapy for Giant Axonal Neuropathy. Mol Ther Methods Clin Dev. 2018;9:160-171. Published 2018 Feb 15. doi:10.1016/j.omtm.2018.02.005

Behne R, Teinert J, Wimmer M, et al. Adaptor protein complex 4 deficiency: a paradigm of childhood-onset hereditary spastic paraplegia caused by defective protein trafficking. Hum Mol Genet. 2020;29(2):320-334. doi:10.1093/hmg/ddz310

Bohannon RW, Smith MB. Interrater reliability of a modified Ashworth scale of muscle spasticity. Phys Ther. 1987 Feb;67(2):206-7. doi: 10.1093/ptj/67.2.206. PMID: 3809245.

Bradbury AM, Bagel JH, Nguyen D, et al. Krabbe disease successfully treated via monotherapy of intrathecal gene therapy. J Clin Invest. 2020;130(9):4906-4920. doi:10.1172/JCI133953

Bucher T, Colle MA, Wakeling E, Dubreil L, Fyfe J, Briot-Nivard D, Maquigneau M, Raoul S, Cherel Y, Astord S, Duque S, Marais T, Voit T, Moullier P, Barkats M and Joussemet B (2013) scAAV9 Intracisternal Delivery Results in Efficient Gene Transfer to the Central Nervous System of a Feline Model of Motor Neuron Disease. Hum Gene Ther 24:670-82. doi: 10.1089/hum.2012.218

Burgos PV, Mardones GA, Rojas AL, et al. Sorting of the Alzheimer's disease amyloid precursor protein mediated by the AP-4 complex. Dev Cell. 2010;18(3):425-436. doi:10.1016/j.devcel.2010.01.015

Caviness VS, Kennedy DN, Richelme C, Rademacher J, Filipek PA. The human brain age 7-11 years: a volumetric analysis based on magnetic resonance images. Cereb Cortex 1996;6(5):726-36 doi: 10.1093/cercor/6.5.726.

Chen X, Snanoudj-Verber S, Pollard L, et al. Pre-clinical Gene Therapy with AAV9/AGA in Aspartylglucosaminuria Mice Provides Evidence for Clinical Translation. Mol Ther. 2021;29(3):989-1000. doi:10.1016/j.ymthe.2020.11.012

Choudhury SR, Hudry E, Maguire CA, Sena-Esteves M, Breakefield XO and Grandi P (2017). Viral vectors for therapy of neurologic diseases. Neuropharmacology120:63-80. doi: 10.1016/j.neuropharm.2016.02.013

Davies AK, Itzhak DN, Edgar JR, et al. AP-4 vesicles contribute to spatial control of autophagy via RUSC-dependent peripheral delivery of ATG9A. Nat Commun. 2018;9(1):3958. Published 2018 Sep 27. doi:10.1038/s41467-018-06172-7.

De Pace R, Skirzewski M, Damme M, et al. Altered distribution of ATG9A and accumulation of axonal aggregates in neurons from a mouse model of AP-4 deficiency syndrome. PLoS Genet. 2018;14(4):e1007363. Published 2018 Apr 26. doi:10.1371/journal.pgen.1007363.

Dekaban AS. Changes in brain weights during the span of human life: relation of brain weights to body heights and body weights. Ann Neurol 1978;4(4):345-56 doi: 10.1002/ana.410040410.

Ebrahimi-Fakhari D, Behne R, Davies AK, Hirst J. AP-4-Associated Hereditary Spastic Paraplegia. In: Adam MP, Ardinger HH, Pagon RA, et al., eds. GeneReviews®. Seattle (WA): University of Washington, Seattle; 1993, updated 2018.

Ebrahimi-Fakhari D, Teinert J, Behne R, et al. Defining the clinical, molecular and imaging spectrum of adaptor protein complex 4-associated hereditary spastic paraplegia. Brain. 2020;143(10):2929-2944. doi:10.1093/brain/awz307

Gray SJ, Matagne V, Bachaboina L, Yadav S, Ojeda SR and Samulski RJ (2011) Preclinical Differences of Intravascular AAV9 Delivery to Neurons and Glia: A Comparative Study of Adult Mice and Nonhuman Primates. Mol Ther. (6):1058-1069.

Gray SJ. Timing of Gene Therapy Interventions: The Earlier, the Better. Mol Ther. 2016;24(6):1017-1018. doi:10.1038/mt.2016.20

Harrison T, Graham F, Williams J (1977). Host-range mutants of adenovirus type 5 defective for growth in HeLa cells. Virology, 77:319-329.

Haurigot V, Marco S, Ribera A, Garcia M, Ruzo A, Villacampa P, Ayuso E, Anor S, Andaluz A, Pineda M, Garcia-Fructuoso G, Molas M, Maggioni L, Munoz S, Motas S, Ruberte J, Mingozzi F, Pumarola M and Bosch F (2013) Whole body correction of mucopolysaccharidosis IIIA by intracerebrospinal fluid gene therapy. J Clin Invest. doi: 10.1172/JCI66778

Hirst J, Irving C, Borner GH. Adaptor protein complexes AP-4 and AP-5: new players in endosomal trafficking and progressive spastic paraplegia. Traffic. 2013;14(2):153-164. doi:10.1111/tra.12028

Ivankovic D, Drew J, Lesept F, et al. Axonal autophagosome maturation defect through failure of ATG9A sorting underpins pathology in AP-4 deficiency syndrome. Autophagy. 2020;16(3):391-407. doi:10.1080/15548627.2019.1615302

Jameel M, Klar J, Tariq M, et al. A novel AP4M1 mutation in autosomal recessive cerebral palsy syndrome and clinical expansion of AP-4 deficiency. BMC Med Genet. 2014;15:133. Published 2014 Dec 14. doi:10.1186/s12881-014-0133-2

Manns MP, Czaja AJ, Gorham JD, et al. Diagnosis and management of autoimmune hepatitis. Hepatology. 2010;51(6):2193-2213. doi:10.1002/hep.23584

Markakis EA, Vives KP, Bober J, Leichtle S, Leranth C, Beecham J, Elsworth JD, Roth RH, Samulski RJ and Redmond DE, Jr. (2010) Comparative transduction efficiency of AAV vector serotypes 1-6 in the substantia nigra and striatum of the primate brain. Mol Ther 18:588-93.

Masamizu Y, Okada T, Kawasaki K, et al. Local and retrograde gene transfer into primate neuronal pathways via adeno-associated virus serotype 8 and 9. Neuroscience. 2011;193:249-258. doi:10.1016/j.neuroscience.2011.06.080.

Matsuda S, Miura E, Matsuda K, et al. Accumulation of AMPA receptors in autophagosomes in neuronal axons lacking adaptor protein AP-4. Neuron. 2008;57(5):730-745. doi:10.1016/j.neuron.2008.02.012

Mattera R, Park SY, De Pace R, Guardia CM, Bonifacino JS. AP-4 mediates export of ATG9A from the trans-Golgi network to promote autophagosome formation. Proc Natl Acad Sci U S A. 2017;114(50):E10697-E10706. doi:10.1073/pnas.1717327114

Morgan CJ, Pyne-Geithman GJ, Jauch EC, Shukla R, Wagner KR, Clark JF and Zuccarello M (2004). Bilirubin as a cerebrospinal fluid marker of sentinel subarachnoid hemorrhage: a preliminary report in pigs. J Neurosurg 101:1026-1029

Mutlu A, Livanelioglu A, Gunel MK. Reliability of Ashworth and Modified Ashworth scales in children with spastic cerebral palsy. BMC Musculoskelet Disord. 2008;9:44. Published 2008 Apr 10. doi:10.1186/1471-2474-9-44.

Nathwani AC, Tuddenham EG, Rangarajan S, et al. Adenovirus-associated virus vector-mediated gene transfer in hemophilia B. N Engl J Med 2011;365(25):2357-65 doi: 10.1056/NEJMoa1108046

Nathwani AC, Reiss UM, Tuddenham EG, et al. Long-term safety and efficacy of factor IX gene therapy, 2014

Pardridge WM. 1991. Title: Peptide drug delivery to the brain, Chapter: Transnasal and intraventricular delivery of drugs. Raven Press, New York. (ISBN: 0881677930 9780881677935)

Pardridge WM. Drug transport in brain via the cerebrospinal fluid. Fluids Barriers CNS 2011;8(1):7 doi: 10.1186/2045-8118-8-7.

Peterson MR, Cherukuri V, Paulson JN, et al. Normal childhood brain growth and a universal sex and anthropomorphic relationship to cerebrospinal fluid. J Neurosurg Pediatr. 2021;28(4):458-468. Published 2021 Jul 9. doi:10.3171/2021.2.PEDS201006

Samaranch L, Salegio EA, San Sebastian W, Kells AP, Bringas JR, Forsayeth J and Bankiewicz KS (2013) Strong cortical and spinal cord transduction after AAV7 and AAV9 delivery into the cerebrospinal fluid of nonhuman primates. Hum Gene Ther 24:526-32. doi: 10.1089/hum.2013.005

Samaranch L, Salegio EA, San Sebastian W, Kells AP, Foust KD, Bringas JR, Lamarre C, Forsayeth J, Kaspar BK and Bankiewicz KS (2012) Adeno-associated virus serotype 9 transduction in the central nervous system of nonhuman primates. Hum Gene Ther 23:382-9. doi: 10.1089/hum.2011.200

Saraiva J, Nobre RJ and Pereira de Almeida L (2016). Gene therapy for the CNS using AAVs: The impact of systemic delivery by AAV9. J Control Release 241:94-109. doi: 10.1016/j.jconrel.2016.09.011.

Snyder BR, Gray SJ, Quach ET, Huang JW, Leung CH, Samulski RJ, Boulis NM and erici T (2011) Comparison of Adeno-Associated Viral Vector Serotypes for Spinal Cord and Motor Neuron Gene Delivery. Hum Gene Ther. 22(9):1129-1135

Sullivan HG, Miller DJ, Griffith RL, Carter W and Rucker S (1979). Bolus versus steady-state infusion for determination of CSF outflow resistance. Ann. Neurology. 5:228-238

Toh WH, Tan JZ, Zulkefli KL, Houghton FJ, Gleeson PA. Amyloid precursor protein traffics from the Golgi directly to early endosomes in an Arl5b- and AP4-dependent pathway. Traffic. 2017;18(3):159-175. doi:10.1111/tra.12465

Zacharia A, Zimine S, Lovblad KO, Warfield S, Thoeny H, Ozdoba C, Bossi E, Kreis R, Boesch C, Schroth Gand Hüppi PS (2006). Early Assessment of Brain Maturation by MR Imaging Segmentation in Neonates and Premature Infants. American Journal of Neuroradiology. 27:972-977

Clinical Trial Protocol

A Phase 1 Open-label Intrathecal Administration of MELPIDA to Determine the Safety and Efficacy for Patients with Spastic Paraplegia Type 50 (SPG50) caused by a Mutation in the AP4M1 gene

**Clinical Trial Protocol #:**	MELPIDA for SPG50
**Protocol Version #:**	6
**Protocol Date:**	June 24th, 2022
**Phase of Study:**	Phase I
**Sponsor:**	James Dowling, MD, PhD and<br>The Hospital for Sick Children
**Sponsor Address:**	The Hospital for Sick Children<br>555 University Avenue<br>Toronto, ON Canada M5G 1X8
**HC**	TBD
**FDA IND**	TBD

CONFIDENTIALITY STATEMENT

This document contains confidential information, which should not be copied, referred to, released or published without written approval from The Hospital for Sick Children and James Dowling. Investigators are cautioned that the information given in this brochure might be subject to change and revision. Any conclusion regarding efficacy and safety must be considered provisional.

SIGNATURE PAGE AND REVISION HISTORY

______________________________ ______________________________

Dr J Dowling, MD Date

The Hospital for Sick Children
555 University Ave
Toronto, ON
M5G 1X8

Amendments: The following sections/appendices have been updated:

Section/Appendix	Description	Date
Amendment 1 [date]		

TABLE OF CONTENTS

1. STUDY SYNOPSIS 7

2. INTRODUCTION AND BACKGROUND 9

3. RATIONALE FOR THE STUDY 10

4. STUDY OBJECTIVES & ENDPOINTS 11
4.1. Primary Objectives 11
4.2. Primary EndpPoints 11
4.3. Secondary Objectives 11
4.4. Secondary Endpoints 11
4.5. Exploratory EndpointsObjectives 11

5. INVESTIGATIONAL PLAN 12
5.1. Study Design 12
5.2. Investigational Product 12
5.3. Packaging 12
5.4. Labeling and Storage 12
5.5. Dose and Route 12
5.5.1. Dose Selection Rationale 13

6. SCHEDULE OF EVENTS 18
6.1. Screening: 18
6.2. Enrollment and pre-dosing schedule: 19
6.3. Dosing day: 20
6.3.1. Administration of Study intervention (MELPIDA): 21
6.3.2. Anesthesia Safety 21
6.3.3. Post-Procedure Recovery 21
6.4. Day 2 21
6.5. Days 7, 14, 21, 28 (+/- 2 days) 22
6.6. Months 3, 6, 9 and 12 (+/- 14 days) 22
6.7. Months 18, 24, 36, 48, 60 (+/- 14 days) 22
6.8. Specific Study Procedures 26
6.8.1. Nerve conduction study (NCS) 26
6.8.2. Magnetic Resonance Imaging of Brain (MRI) 26
6.8.3. Liver Ultrasound 26
6.8.4. Lumbar puncture 26
6.9. Adverse Event and Concomitant Medication Monitoring 27
6.10. Immune Modulation Protocol 27

7. INCLUSION/EXCLUSION CRITERIA 29
7.1. Inclusion Criteria 29
7.2. Exclusion Criteria 29
7.3. Participant Withdrawal 30

8. STATISTICAL ANALYSIS 31
8.1. Data Monitoring 31
8.1.1. General Plan 31
8.1.2. Monitoring Entity 31

8.2. Plans for Assuring Participant Safety, Adverse Event Collection, and Reporting 31
8.3. Definitions 32
8.3.1. Adverse Event 32
8.3.2. Classification of Adverse Events 32
8.4. Dose Limiting Toxicity 34
8.5. Reporting Procedures to the REB 35
8.6. Reporting Procedures to Health Canada 35
8.7. Reporting Procedures to the Research Ethics Board 36
8.8. Protocol Deviations and Continuing Review 37
8.9. Stopping Rules 37

9. DATA COLLECTION 37
9.1. Database Locks 38
9.2. Study Monitoring Plan 38
9.3. Quality Assurance of Data 38

10. INSTRUMENTS FOR THE ASSESSMENT OF DISEASE 39
10.1. Modified Ashworth Scale 39
10.2. Tardieu Scale 40

11. PRIVACY AND CONFIDENTIALITY 42

12. REFERENCES 43

List of Abbreviations

AAV	adeno-associated virus
Ab	antibody
AE	Adverse event
Ag	antigen
ALT	Alanine aminotransferase
ALP	Alkaline phosphatase
AP-4	Adaptor Protein complex
AP4M1	Adaptor protein complex, μ4
AST	Aspartate aminotransferase
ATG9A	Autophagy Related 9A
BCH	Boston Children's Hospital
BGH	Bovine growth hormone
BUN	Blood urea nitrogen
Ca	calcium
CBC	Complete blood counts
CDMO	Contract development and manufacturing organization
CK-MB	Creatine kinase – isotype MB
Cl	chloride
CMC	Chemistry, manufacturing and controls
CNS	Central nervous system
CO_2	Carbon dioxide
Cr	Creatinine
CRP	C-reactive protein
CSF	Cerebrospinal fluid
CTA	Clinical Trial Application
DAPI	4′,6-diamidino-2-phenylindole
DNA	Deoxyribonucleic acid
DTI	Diffusion tensor imaging
EEG	electroencephalogram
EKG	electrocardiogram
ESR	Erythrocyte sedimentation rate
FDA	Food and Drug Administration
GFP	Green fluorescent protein
GGT	Gamma-glutamyl transferase
GLP	Good laboratory practice
h	Human
hAP4M1opt	Human optimized AP4M1
HSP	Hereditary spastic paraplegia
HC	Health Canada
HCT	hematocrit
HIV	Human immunodeficiency virus
HTLV1	Human T-Lymphotropic Virus Type 1
IND	Investigational New Drug
INR	International normalized ratio
iPSCs	Induced pluripotent stem cells
IRNHS	International registry Natural History Study
IT	Intrathecal

ITR	Inverted terminal repeat(s)
K	potassium
kg	Kilogram
LFT	Liver function tests
LP	Lumbar puncture
MCV	Mean corpuscular volume
MOI	Multiplicity of Infection
MRI	Magnetic resonance imaging
Na	sodium
NCS	Nerve conduction studies
NAb	Neutralizing antibody
NHP	Non human primates
NIH	National Institutes of Health
opt	Optimized
OOPD	Office of Orphan Products Development
PACU	Post anesthesia care unit
PBMC	Peripheral blood mononuclear cell
PI	Principal investigator
PICU	Pediatric intensive care unit
PLT	platelets
PPD	Purified protein derivative
ProBNP	Pro B-type Natriuretic peptide
PT	Prothrombin time
PTT	Partial Prothrombin time
sc	Self-complimentary
SickKids	The Hospital for Sick Children
SPG50	Spastic Paraplegia 50
RNA	Ribonucleic acid
SAE	Serious adverse event
SUSAR	Suspected unexpected serious adverse reaction
TB	tuberculosis
TGN	Trans-Golgi network
Tn	troponin
US	Unites States of America
UTSW	University of Texas Southwestern Medical Center
VCC	Viralgen Vector Core, Spain
vg	Vector genome(s)
WT	Wild type

1. STUDY SYNOPSIS

Title	A Phase 1 Open-label Intrathecal Administration of MELPIDA to Determine its Safety and Efficacy for Patients with Spastic Paraplegia Type 50 (SPG50) caused by Mutation in the AP4M1 gene.
Study Description	This will be a first-in-human Phase I, open-label, single dose clinical study of MELPIDA administered intrathecally (IT) through a lumbar puncture (LP) to a single subject with confirmed pathogenic mutations in the AP4M1 gene.
Number of Subjects	N = 1
Clinical Study Phase	Phase I
Sponsor	Dr James Dowling and The Hospital for Sick Children, Toronto
PI	Dr. James Dowling
Study Objectives	Primary outcome: determination of the safety and tolerability of MELPIDA in patients with SPG50, based on development of toxicity<br>Secondary outcome: preliminary exploration of efficacy
Study Intervention	MELPIDA, a recombinant serotype 9 adeno-associated virus (AAV) encoding a codon-optimized human AP4M1 transgene
Study Dose	A single intrathecal infusion of 10 mL at 1E14 vg/mL for a total dose of 1E15 vg
Study Population	Children with a confirmed mutation in the AP4M1 gene
Study Duration	The total study duration is 5 years post dosing.<br>The participant will be tested at screening/baseline (-28 to -7 days), return for dosing, and then follow-up visits post-dosing on Days 7 (+/-2), 30 (+/-2), 60 (+/-2), 90 (+/-14), 180 (+/-14), 270 (+/-14), 360 (+/-14), 540 (+/-14), and 720 (+/-14) days, then annually for the last 3 years.
Inclusion Criteria	• Age < 5 years old<br>• Confirmed diagnosis of SPG50 disease by:<br>• Genomic DNA mutation analysis demonstrating homozygous or compound heterozygous, pathogenic and/or potentially pathogenic variants in the *AP4M1* gene<br>• Clinical history or examination features consistent with SPG50 and that include neurologic dysfunction<br>• Parent/legal guardian willing to provide written informed consent for their child prior to participation in the study<br>• Subject able to comply with all protocol requirements and procedures
Exclusion Criteria	• Inability to participate in study procedures (as determined by the site investigator)<br>• Presence of a concomitant medical condition that precludes lumbar puncture (LP) or use of anesthetics<br>• History of bleeding disorder or any other medical condition or circumstance in which lumbar puncture is contraindicated according to local institutional policy<br>• Inability to be safely sedated in the opinion of the clinical anesthesiologist<br>• Active infection, at the time of dosing, based on clinical observations

	• Concomitant illness or requirement for chronic drug treatment that in the opinion of the PI creates unnecessary risks for gene transfer<br>• Inability of the patient to undergo MRI according to local institutional policy<br>• Inability of the patient to undergo any other procedure required in this study<br>• The presence of significant non-SPG50 related CNS impairment or behavioral disturbances that would confound the scientific rigor or interpretation of results of the study<br>• Have received an investigational drug within 30 days prior to screening or plan to receive an investigational drug (other than gene therapy) during the study.<br>• Enrollment and participation in another interventional clinical trial<br>• Contraindication to MELPIDA or any of its ingredients<br>• Contraindication to any of the immune suppression medications used in this study<br>• Clinically significant abnormal laboratory values (GGT, ALT, and AST, or total bilirubin > 3 × ULN, creatinine ≥ 1.5 mg/dL, hemoglobin [Hgb] < 6 or > 20 g/dL; white blood cell [WBC] > 20,000 per cmm) prior to gene replacement therapy. Patients with an elevated bilirubin level that is unequivocally the result of neonatal jaundice shall not be excluded
Study Design	This will be a first-in-human Phase I, open-label, single dose clinical study of MELPIDA administered intrathecally through a lumbar puncture to a single subject with confirmed pathogenic mutations in the AP4M1 gene.
Primary Endpoints	Incidence of anticipated treatment-related toxicities, Grade 3 or higher, will be determined from the collection of occurrence and severity of serious adverse events (SAEs). In addition, change from baseline in nerve conduction velocity and amplitude, and determination of liver safety (e.g. laboratory and image studies) will be conducted at timepoints listed in the Schedule Of Events
Secondary Endpoints	Efficacy will be determined by the stability or improvement in spasticity as assessed using the Modified Ashworth scale (MAS) and Tardieu scale.
Exploratory Endpoints	• Bayley Scales of Infant and Toddler Development 4th edition (Fine Motor, Gross Motor, Cognitive & Language)<br>• Vineland (Comprehensive Parent/Caregiver Form)<br>• Log Book of Seizure frequency and duration<br>• Log Book of Number of Falls<br>• Clinical Global Impression of Overall Change by Physician (CGI)
Sample Size	This is a single dose study in a single subject

The Schedule of Events can be found in Table 2.

2. INTRODUCTION AND BACKGROUND

MELPIDA is a gene therapy product being developed for the treatment of Spastic Paraplegia Type 50 (SPG50), which is one of a group of four genetic disorders (SPG47, SPG50, SPG51 and SPG52) comprising AP-4 related Spastic Paraplegia (AP4-SPG). Inherited in an autosomal recessive pattern, AP-4- SPG is caused by biallelic pathogenic variants in one of 4 genes that encode components of the heterotrimeric adaptor protein complex 4 (AP4). Mutations in any of the components result in disrupted AP-4 function, and result in a common, shared clinical phenotype (Behne et al, 2020; Ebrahimi-Fakhari et al, 2020; Ebrahimi-Fakhari et al, 2018). Adaptor protein complexes such as AP-4 play key roles in signal-mediated trafficking of integral membrane proteins. They mediate vesicle formation and the cargo contained within these vesicles (Jamra et al, 2011). While the precise function of the AP-4 complex is not fully understood, recent data suggests it plays an important role in protein sorting through the golgi, including regulation of trafficking of components required for autophagy (Davies et al., 2018). Deficiency in AP-4 leads to progressive neurodegeneration.

AP-4-HSP is an ultra-rare autosomal recessive disease with ~156 patients identified worldwide, 59 of which have the SPG50 subtype. There are approximately 9 patients with SPG50 in North America (OMIM #612936) (source: Ebrahimi-Fakhari et al, 2020), ClinicalTrials.gov Identifier: NCT04712812. SPG50 is caused by biallelic pathogenic variants in the AP4M1 gene.

The AP4-deficiency syndrome (AP-4-HSP) is characterized by progressive spasticity, microcephaly, intellectual deficiency, dysmorphic traits, and growth retardation (Roubertie et al., 2018). Symptoms of AP-4-HSP begin in infancy, though patients are often not correctly identified and diagnosed until age 5 to 10 years. Patients experience progressive spastic paraplegia in the first decade of life, resulting in quadriplegia by adolescence or early adulthood with associated wheelchair dependence. There is also the presence of severe, progressive cognitive impairment. Epilepsy is an important co-morbidity present in the majority of cases (Ebrahimi-Fakhari et al, 2018). Only a few affected individuals have been identified to survive beyond age 30 year, though the extent of early mortality is yet to be fully elucidated (Ebrahimi-Fakhari et al, 2020).

Based on an AP-4-HSP natural history study currently in progress at Boston Children’s Hospital (BCH), it is evident that disease severity ranges from child to child, but that most children fall into the severely affected (i.e. severe spasticity with paralysis and severe cognitive impairment) category. A small proportion of children, considered least severe, are able to speak in short sentences, walk with an abnormal gait, and have few to no seizures early on in the disease (less than 10 years of age). However, most children in this less severe category still experience progressive decline, ultimately losing the ability to walk and becoming quadriplegic between the ages of 10 and 20 years.

The majority of children with the SPG50 subtype of AP-4-HSP conform to a severe presentation, and are completely non-verbal, have microcephaly, never walk, have epilepsy and are severely cognitively impaired by the age of 10. It is not known how patients are affected later in life as very few have been identified beyond the age of 30. SPG50 is thus a degenerative neurological disease, affecting both cognitive and motor capabilities. Importantly, there is significant care

giver burden, as all patients eventually require complete support for all activities of daily living from family and/or caregivers. There are no treatments currently available for patients with SPG50.

Additional details on the disease pathophysiology and its progression are available in the Investigator's Brochure (Section 2).

3. RATIONALE FOR THE STUDY

SPG50 is a monogenetic disease caused by biallelic variants in the *AP4M1* gene. *APM41* encodes a subunit of the AP complex; mutations in *APM41* result in failure to produce functional APM41 protein and impairment of the AP complex. Due to the small size of the *APM41* gene, and the loss of expression/function nature of AP4M1 mutations, SPG50 is ideally suited for gene replacement therapy. MELPIDA is a recombinant serotype 9 adeno-associated virus (AAV9) encoding a codon-optimized human AP4M1 transgene (Figure 1). The final product consists of AAV9 capsids that are packaged with the self-complementary AAV genome comprising a mutant AAV2 inverted terminal repeat (ITR) with the D element deleted, the synthetic "UsP" promoter, codon-optimized human AP4M1 deoxyribonucleic acid (DNA) coding sequence, the bovine growth hormone (BGH) polyadenylation signal, and wild-type AAV2 ITR. As a gene therapy, MELPIDA is expected to provide a fully functional human AP4M1 cDNA copy to targeted neuronal and non-neuronal cells of the participant. Production of a fully functional AP4M1 subunit is hypothesized to halt neurodegeneration through the production of functional AP complex.

Figure 1 Schematic of MELPIDA

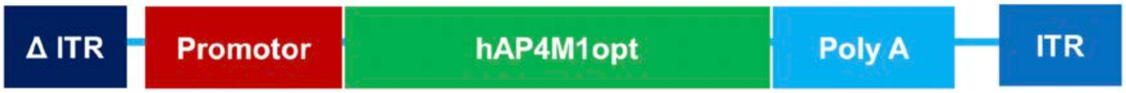

MELPIDA is an AAV9-based gene therapy vector that expresses the fully functional form of AP4M1 under thecontrol of a synthetic promoter. MELPIDA will be delivered intrathecally and is designed to achieve stable, potentially life-long expression of AP4M1 in non-dividing cells. This clinical study is a first-in-human study designed to assess safety and tolerability of MELPIDA in SPG50 participants, as well as examine the clinical impact of the gene therapy on disease progression.

Numerous investigators have utilized recombinant AAV9 directed at the central nervous system (CNS) in on-going gene therapy clinical trials (clinical trial.gov identifiers NCT02122952, NCT02362438, NCT02725580,NCT02716246, NCT03315182). These vectors are non-pathogenic, non-replicating, and transduce non-dividing cells. However, the recombinant vectors are incapable of coding viral proteins or actively integrating with the hostgenome, making them

ideal vectors for gene delivery. Additionally, AAV9 can be purified in large quantities at high concentrations for potential use in delivering a functional copy of a gene to cells with aberrant, disease-causing mutations. In disorders of neurologic origin, targeted CNS-focused administration (via intrathecal administration) achieves broad transgene distribution throughout the CNS. An approach utilizing intrathecal (IT) administration of AAV9 was first advanced as a treatment of Giant Axonal Neuropathy (GAN). The laboratory of Dr. Steven Gray, in partnership with Hannah's Hope Fund, initiated the first intrathecal AAV9 gene therapy trial, which was a first-in-human Phase I gene therapy clinical trial for GAN, in collaboration with Dr. Carsten Bonnemann at the US National Institutes of Health Clinical Center (NCT02362438) in 2015.

There are no approved treatments for SPG50, leaving an unmet medical need for this serious, rare, progressive, and ultimately fatal neurodegenerative disease.

4. STUDY OBJECTIVES & ENDPOINTS

4.1. Primary Objectives

The primary objective of this study is to evaluate the safety and tolerability of a single dose of MELPIDA administered intrathecally to a single child with SPG50 disease.

4.2. Primary Endpoints

Incidence of unanticipated anticipated treatment-related toxicities, Grade 3 or higher, will be determined from the collection of occurrence and severity of serious adverse events (SAEs). In addition, change from baseline in nerve conduction velocity and amplitude, and determination of liver safety (e.g. laboratory and image studies) will be conducted at timepoints listed in the Schedule Of Events

4.3. Secondary Objectives

The secondary objectives will be efficacy of the drug.

4.4. Secondary Endpoints

Efficacy will be determined by the stability or improvement in spasticity as assessed using the Modified Ashworth scale (MAS) and Tardieu scale. These assessments are summarized in the Table 2 and explained in section 6.4.2. 6.0 Investigational Plan

4.5. Exploratory Endpoints

Participants will undergo motor function, neuropsychological, and disease burden assessments every 3 months starting at screening/baseline to 24 months, then annually until 5 years post-dose. Additional assessments to evaluated as exploratory objectives include:

- Bayley 4(Fine Motor, Gross Motor, Cognitive & Language)
- Vineland (Comprehensive Parent/Caregiver Form)
- Log Book Seizures
- Log Book # Of Falls
- Clinical Global Impression of Overall Change by Physician (CGI)

5. INVESTIGATIONAL PLAN

5.1. Study Design

This will be a first-in-human Phase I, open-label, single dose clinical study of MELPIDA administered intrathecally (IT) through a lumbar puncture (LP) in a single subject with confirmed pathogenic mutations in the AP4M1 gene and clinical signs/symptoms of SPG50 disease.

5.2. Investigational Product

MELPIDA vials will be formulated as a concentrated stock in phosphate-buffered saline (PBS) containing 5% D-sorbitol and 0.001% Poloxamer 188, and stored at ≤ -60˚C until the day of the administration. The solution will be thawed within 4 hours prior to administration and diluted to the appropriate final dosage concentration and volume using PBS with 5% D-sorbitol and 0.001% Poloxamer 188 (if necessary).

5.3. Packaging

MELPIDA is supplied as a 2 mL Daikyo CZ® vial containing 1.15 mL of a sterile clear solution. Each mL contains 1E14 vector genome-containing particles (vg) of MELPIDA in phosphate buffered saline (PBS) containing 5% sorbitol and 0.001% Poloxamer 188

5.4. Labeling and Storage

MELPIDA and diluent are labeled with the lot/batch number, individual vial number, contents, and manufacture date, along with a warning that they are for investigational use only. They will be stored at or below -80˚C in Room 11123D at the Hospital for Sick Children. This is a locked, certified and monitored freezer located within the Hospital for Sick Children, Research Pharmacy, where the drug product will be stored.

5.5. Dose and Route

The participant will have a spinal needle inserted percutaneously at the lumbar level into the intrathecal space of the spinal column (L4/L5 interspace). A volume of CSF approximately equal to the infusion volume is withdrawn from the lumbar thecal sac. With the patient in the

Trendelenberg position (head down), the vector solution is then infused at a rate of 1 mL per minute for a total of 10 mL for participants 4 years of age and older (see Table 1 for volume adjustments for younger participants). The participant will remain side-lying in the Trendelenburg position (head down) at 15 degrees for one (1) hour following administration, during which time the patient will be turned (from left to right side/right to left side) every 15 minutes. Dosing volumes will be calculated per Table 1, depending on final vector product concentration. The procedure will be performed in a procedure unitwith an anesthesiologist or qualified physician present to administer sedation as needed. Participant will stay in the Pediatric Intensive Care Unit (PICU) overnight. As with the NIH GAN study, an immune suppression regimen will be utilized. Prophylactic enteral prednisone or prednisolone and sirolimuswill be administered to participant. Additional immunosuppression with tacrolimus will be administered as defined in the immune modulation protocol (Section 6.10).

Table 1 Dose Extrapolation Based on Age and Brain Size

Age (years)	Brain Volume (approx. cm3)	Infusion volume (mL)	Total IT High Dose (E14 vg)
4+	1312	10	10
3	1180	9	9
2	1080	8.2	8.2
1	955	7.3	7.3
0.5	525	4	4
Newborn	400	3	3

5.5.1. Dose Selection Rationale

5.5.1.1. Justification of clinical study dose

Nonclinical studies have evaluated toxicity, safety, tolerability, expression and biodistribution of MELPIDA in various models including normal (C57BL/6J) mice, an Ap4m1 knock-out (KO) mouse model of SPG50, Sprague Dawley (SD) rats and non-human primates (NHPs). In vitro studies have also been conducted in patient derived fibroblasts.

These studies support the following conclusions:

- Fibroblasts from 2x patients with SPG50 transduced with MELPIDA (using an AAV2 capsid) restored autophagy related 9A (ATG9A) trafficking and hence AP4 function, at multiplicity of infection (MOI)s of 1E2 to 1E5 vg/cell. This study showed a dose-dependent reduction in ATG9A staining at the TGN, AP4E1 localization to the TGN and unchanged staining for TGN46 with phenotypic rescue in up to 77% of fibroblasts and no associated toxicity.
- An in vivo efficacy study is ongoing in WT, heterozygous and homozygous Ap4m1 knock out (KO) mice dosed intrathecally at post-natal day (PND) 7 to 10 or PND 90 with no

treatment, vehicle, low (1.25E11), mid (2.5E11) or high (5E11) doses of MELPIDA and assessed for potential phenotypic rescue. Interim results demonstrated that MELPIDA increased hAP4M1opt mRNA in all brain regions at 3 weeks post dosing, and improved impaired behaviors, induced minimal immune responses, and did not lead to elevation of serum markers of toxicity at 5 and 8 months post dosing.

- An in vivo 12-month non-GLP toxicology study was carried out in WT C57BL/6J mice dosed intrathecally at the age of 7 weeks with vehicle, low (1.25E11), or high (5E11) doses of MELPIDA. Results demonstrated that MELPIDA was generally safe and well tolerated. Dose-dependent hAP4M1opt mRNA expression was noted in all brain regions at 4 weeks post IT injection, with expression sustained up to at least 12 months post infusion, confirming that MELPIDA reached and achieved transgene expression at the targeted site of action. There were no effects on body weight, hematology or clinical signs; minimal effects on clinical chemistry were noted. Several male animals were found to have hepatocellular adenoma's which are expected in these mice as they age (up to 51% in males aged 9 to 15m).
- An in vivo 3-month GLP toxicology and biodistribution study in WT Sprague Dawley (SD) rats dosed intrathecally at the age of 7 weeks with 0 (vehicle), 0.36E12, 1.1E12, or 3.3E12 vg/rat of MELPIDA was well tolerated. Findings were limited to neurobehavioral effects such as increased excitability and activity and decreases in body weight at 3.3E12 vg, and microscopic findings in the lumbar dorsal nerve roots, lumbar dorsal root ganglion, cauda equina in the injection site, and peripheral nerves (sciatic/tibial nerves). Due to the nature of the neuronal degeneration noted in the lumbar dorsal root ganglion at ≥ 1.1E12 vg and the absence of recovery in this finding, it was considered adverse. Based on these results, the no-observed-adverse-effect level (NOAEL) was considered to be 3.6E11 vg.
- An in vivo 3-month non-GLP toxicology and biodistribution study is ongoing in WT Cynomolgus monkeys (Macaca fascicularis) dosed intrathecally at the age of 2 to 4 years with 0 (vehicle), 8.4E13, or 1.68E14 vg/MELPIDA. Preliminary results indicate MELPIDA was well tolerated with no animals needing to be euthanized. There were no effects on body weight, weight gains, or appetite. There were no MELPIDA-related clinical observations. Clinical observations including, but not limited to, hunched posture, erected fur, tremors, decreased muscle tone, and decrease activity, were considered procedure-related and not due to the administration of MELPIDA due to their low incidence, transient or sporadic nature, or similar incidence in the control group. MELPIDA-related neurological changes were noted on Day 94 for Male No. 3201 and consisted of abnormal general attitude and motor function (slight tremors in the hindlimbs) and decreased proprioceptive positioning in the left hindlimb. There was no irritation noted at the puncture site. Intrathecal administration of MELPIDA at 1.68E14 vg resulted in a slight decrease in mean sural nerve conduction velocity of 11% on Day 45, relative to the control group, accompanied by a decrease in mean response amplitude of 27%. Male No. 3201 exhibited the most significant change with nerve conduction velocity of 44 m/sec compared to a mean of 52 m/sec in control animals. On Days 77 and 92, a significant decrease in mean nerve conduction velocity of 26% and 28%, respectively, was noted at 1.68E14 vg. The changes in conduction velocity were accompanied by associated decreases in mean sural nerve response amplitude which was reduced by 69% on Day 77 and by 74% on Day 92. Male No. 3201 was the most impacted, exhibiting the most pronounced changes with nerve conduction velocity of 34 m/sec (39%

change relative to mean control value) on Day 77 and 32 m/sec (36%) on Day 92 compared to 52-53 m/sec in control animals. The most pronounced decrease in response amplitude was also noted in Male No. 3201, particularly on Day 92, during which an amplitude of 1.2 µV was noted compared to a mean amplitude of 10.2 µV in control animals. No changes in peroneal nerve conduction velocity or amplitude and no changes in the onset latency of the cauda equina were noted up to Day 92. In addition, there were no changes in conduction velocity or amplitude for the sural nerve at 8.4E13 vg.

The findings from these various nonclinical studies provide the proof of concept to support the potential of benefit of MELPIDA to patients with SGP50.

Previous toxicology studies in rodents and large animals have indicated that the potential side effects emerging from the AAV9 capsids were minimal and manageable (Gougeon et al, 2021). Clinical experience for intrathecal AAV9 administration is emerging from several active human trials using AAV9–mediated gene replacement for treating CNS disorders initiated starting in 2015 (GAN, NCT02362438; CLN3, NCT03770572; CLN6, NCT02725580; MPS I, NCT03580083; SMA, NCT03461289, CLN7, NCT04737460, GM2, NCT04798235), with no serious safety concerns publicly disclosed from any of those trials. The primary anticipated complications from intrathecal AAV administration are likely to be anti-capsid immune responses, which appear to be manageable with transient immunosuppressive regimens (Gougeon et al, 2021; Ramsingh et al, 2018). Approximately 47% of humans are seropositive for AAV9, and naturally-occurring AAV9 is not known to cause any human disease (Boutin et al, 2010).

Vector distribution of MELPIDA after a single intrathecal administration measured in two studies was consistent with expected AAV9 biodistribution.

Tissue transduction and expression of the biologically active gene product in vivo were demonstrated in both WT rats, and WT and Ap4m1 KO mice. Efficacy endpoints were measured only in the Ap4m1 KO mouse model, which demonstrated a dose response in expression of hAP4M1 mRNA across both sexes, with a dose of 5E11 vg providing maximal and near normalization of behavioral tests and a lower dose of 2.5E11 providing significant (albeit lower) benefit to the mice. Of note, the lowest dose of 1.25E11 vg in mice did not provide a clear behavioral benefit, indicating a minimally effective dose of 2.5E11 vg in mice.

Safety data was gathered in rodents and NHPs. All studies indicated MELPIDA was generally safe and well tolerated at all doses with some neurobehavioral effects such as increased excitability and activity and decreases in body weight in the rat GLP study at 12 weeks post dose at the highest dose of 3.3E12 vg, which corresponds to a human dose of 1.8E15 vg. Other toxicities of note included neuronal degeneration in the lumbar dorsal root ganglion at doses of ≥ 1.1E12 vg with no recovery. The NOAEL was considered to be 3.6E11 vg corresponding to a human dose of 2.0E14 vg. In the NHP non-GLP study, NCV was reduced in the sural nerve in one animal at the 1.68E14 vg dose, corresponding to a human dose of 2E15 vg.

Safety concerns in humans are (i) possible immunological issues and (ii) the potential for DRG toxicity. The immunological issues relate to the possibility of a cytotoxic lymphocyte response against an expressed foreign antigen and the likelihood of a deleterious immune response to the

high intrathecal load of AAV9 capsid. The anti-AAV9 response is theoretical, but dose-responsive pleocytosis has been reported (Gougeon et al, 2021; Abstract #637 by Bharucha-Goebel et al, 2019 at American Society of Gene and Cell Therapy) that was not associated with clinical symptoms. Pleocytosis was managed with an extended steroid regimen and justifies adding additional transient T cell suppression strategies (Chu et al, 2021). These findings support the incorporation of the immune management protocol proposed with MELPIDA treatment in the clinic.

The toxicity noted within the dorsal root ganglia (DRG) in NHPs was a histological finding without any clinical/functional correlate (Hinderer et al, 2018; Hordeaux et al, 2020a). It was noted with MELPIDA in the rat GLP study (CRL-5550008) and presumed in the non-GLP NHP study due to the slowing of NCV in the sural nerve at Day 92 after the highest dose of 1.68E14 vg. However, the clinical significance of this finding in humans remains unknown at the present time.

The proposed dose for this N of 1 patient is 1E15 vg in 10 mL (as the subject will be 4 yo when dosed). When considering the CSF volume in the various species, this corresponds to a dose equivalent of 7.0E12 vg per mL of CSF in humans, mice, rats and NHPs. The comparative absolute vg dose across species is 1E15 vg in humans, 8.4E13 vg in NHPs, 1.8E12 vg in rats, and 2.4E11 vg in mice (Table 15; Figure 22). The pharmacology studies in mice predict a benefit to patients at this dose, considering a dose of 2.5E11 vg in mice provided a clear behavioral benefit with a 5E11 vg dose providing a greater benefit. Of note, 1.25E11 vg (equivalent to 5E14 vg in a human) did not provide a clear behavioral benefit, justifying 1E15 vg as the minimally effective human dose.

Table 1 Relationship between preclinical study doses and proposed human intrathecal dose

Species	Low dose (vg)	Low dose per CSF (vg/mL)	Mid dose (vg)	Mid dose per CSF (vg/mL)	Upper dose (vg)	Upper dose per CSF (vg/mL)	CSF Volume (mL)	HED of NOAEL (vg per mL CSF)	Intended human dose (vg)	Intended human dose (vg/mL)	Safety margin (vg/mL)
Mouse	1.3E11	3.6E12	2.5E11	7.1E12	5.0E11	1.4E13**	0.035	1.4E13			2.12x**
Rat	3.6E11	1.4E12	1.1E12*	4.4E12*	3.3E12	1.3E13	0.25	4.4E12	1E15	6.6E12	0.6x*
NHP	8.4E13*	7.0E12*	-	-	1.7E14	1.4E13	12	7E12			1.1x*
Human ≥4 y							140				

HED, human equivalent dose; CSF Volumes taken from the following references: Morgan et al, 2004; Sullivan et al, 1979; Pardridge, 2011; Pardridge, 1991.
*NOAEL in rat had minimal microscopic findings in lumbar dorsal nerve roots (note NHP study is still preliminary)
**based on upper mouse dose per CSF volume (vg/mL)

Figure 2 Summary of nonclinical findings to support clinical dosing

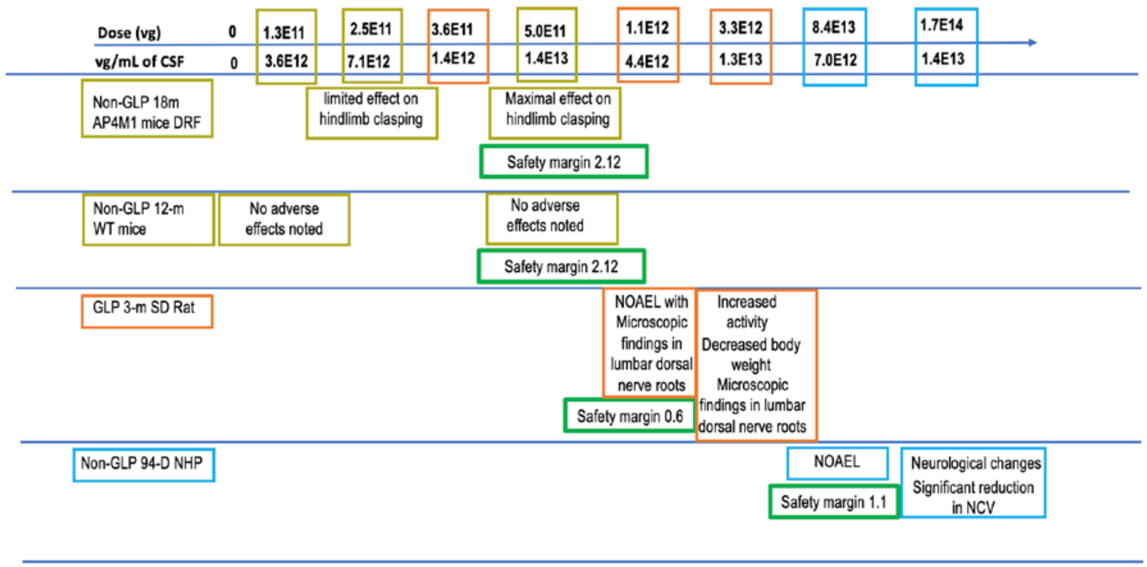

A feature of some SPG50 patients is microcephaly. Since dosing extrapolation across species is based on CSF or brain volume, this raises a possible concern that patients with microcephaly may receive a higher relative dose, since dosing extrapolations assume a patient with normal CSF and brain volume. According to Peterson et al (2021) and Centers for Disease Control growth charts, a patient with microcephaly (3rd percentile) would have a brain volume approximately 6% lower than an average person. A <10% correction factor for dosing would not be expected to have a strong impact on either safety or efficacy. Conversely, the added trial complexity and increased sedation risk to do a detailed volumetric MRI does not seem justified to impose such a corrective dosing factor individualized for each pediatric patient, especially those younger than 4 y. Considering this, the proposed plan is for any patient ≥4 years old to receive the same 1E15 vg dose in 10 mL.

In terms of safety, studies in mice up to 5E11 vg (equivalent to 2.0E15 vg human dose) were tolerated well to one year post-injection, with no significant drug-related effects on survival, body weight, body condition, blood chemistry, or histopathology. Studies in rats found increased activity, reduced weight, and histopathological findings at 3.3E12 vg (equivalent to 1.8E15 vg in humans), whereas lower doses were better tolerated with only sporadic minimal to mild histopathological findings. Studies in NHPs found reduced sensory nerve conduction at 1.7E14 vg (equivalent to 2E15 vg in humans), but otherwise showed minimal in-life adverse effects, and the lower dose of 8.4E13 vg (equivalent to 9.7E14 vg in humans) was tolerated well. Overall, the toxicology studies across 3 species provide safety data up to an approximately 2-fold overdose in the human equivalent dose (HED). While the preclinical toxicology studies predict the possibility of dorsal root ganglion specific pathology at the human 1E15 vg dose, this HED was not associated with adverse clinical findings in the animals. Considering MELPIDA as a one-time treatment for this severe neurodegenerative condition for this single patient without an

option to redose, a proposed dose of 1E15 vg in this single patient should maximize benefit with acceptable risks. Preclinical pharmacology data in the SPG50 mouse model did not clearly support a benefit at any lower dose.

Based on studies in rodents, there is some debated evidence of increased cancer risk associated with AAV vectors (Bolt et al, 2020; Bell et al, 2006; Rosas et al, 2012; Donsante et al, 2007). One large-scale study in mice found no evidence for tumorigenesis following AAV administration (Bell et al, 2005), despite other studies having found evidence for limited AAV integration (Chandler et al, 2015, 2016). While clonal integration of wildtype AAV2 has been detected in patient hepatocellular carcinomas (Nault et al, 2015), integration and increased cancer risk from rAAVs has not been identified in a clinical setting, and it is considered a very low risk for the proposed clinical trial. Moreover, our vector utilizes a relatively weak promoter, minimizing the risk of transactivation (overexpression) of oncogenes if the vector genome integrates nearby.

In summary, AAV9-based AP4M1 gene therapy has promise to treat SPG50 disease, but as with any other new modality, it has some development challenges. Preclinical safety studies in mice, rats, and NHPs demonstrate a favorable safety profile of MELPIDA, at up to twice the proposed human dose of 1E15 vg (doses extrapolated across species by CSF volume, Table 15). Pharmacology studies in the AP4M1 KO mouse model demonstrated a dose-dependent benefit of MELPIDA, with the greatest benefit seen at a dose of 5E11 vg in mice (scaled to approximately 1E15 to 2E15 vg in humans by CSF volume). Importantly, the safety concerns noted in the toxicology studies are balanced against the severe unmet medical need of this patient population and the potential benefit of intrathecal administration of MELPIDA.

6. SCHEDULE OF EVENTS

6.1. Screening:

The participant’s parents/legal guardians will provide written informed consent for their child. The informed consent process can take place remotely via video call; the study and the risks/ benefits to study participation will be discussed in detail. If consent is obtained virtually via video conference, the family will be emailed a copy of the consent form to sign and return electronically. No study procedures will occur prior to consent/assent.

Between 28 and 8 days before potential dosing, the participant will be screened in-person at the Hospital for Sick Children and inclusion/exclusion criteria assessed. If the requirements (all of the inclusion criteria and none of the exclusion criteria) are met, the participant will be enrolled. The PI will also confirm that local care can supply adequate support to the subject in between visits.

At the screening visit, confirmation of genetic diagnosis, medical history, a review of concomitant medications, and the following clinical evaluations will be performed:

- A complete physical exam

- Vital signs – including heart rate, respiration rate, blood pressure, temperature, and oxygen saturation
- Height and weight
- EKG – a 15-lead electrocardiogram will record the participant's electrical heart rhythm
- A neurologic exam to include (but not be limited to) the testing of cranial nerves, muscle bulk/tone andstrength, sensation, cerebellar function, involuntary movements, myotatic reflexes, toe sign, gait, and stance.
- Liver ultrasound
- Blood and urinalysis
    - safety labs - Complete blood count (CBC) with differential, coagulation (INR, PT, PTT), ESR, CRP, Na, K, Cl, Ca, CO_2, BUN, Cr, glucose, ALT, AST, total bilirubins, direct bilirubins, ALP, GGT, serum total protein, cardiac safety panel (Tn, ProBNP, CK-MB), and urinalysis
    - Screening labs - Specifically for screening, the following will be assessed: HIV Ab, Hepatitis A Ab, Hepatitis B Surface Ab, Hepatitis Surface Ag, Hepatitis C Core Ab, PPD skin test for TB, HTLV1
- Spasticity assessments – the Modified Ashworth and Tardieu scales will be administered by a physical therapist to assess spasticity

The following exploratory assessments will also be performed at screening:

- AAV9 Antibody titers
- ELISPOT on whole blood for T cell response to AAV9 and A4PM1
- Bayley Scales of Infant and Toddler Development 4th edition (Fine Motor, Gross Motor, Cognitive & Language)
- Vineland Adaptive Behaviour Scale (Comprehensive Parent/Caregiver Form)
- Log Book of Seizures – starting at the screening visit, the number and duration of seizures will be recorded daily
- Log Book Number of Falls – starting at the screening visit, the number of falls will be recorded daily
- Clinical Global Impression of Overall Change by Physician (CGI)
- Nerve conduction studies

6.2. Enrollment and pre-dosing schedule:

Following the Screening study visit, if the participant is eligible for enrollment, he will begin the immunosuppression regimen. Starting 5-7 days before dosing, the participant will begin

Sirolimus loading at 1mg/m^2 every 4 hours (load only given on one day). Starting the day after the sirolimus load, the sirolimus dose will be lowered to 1 mg/m^2/day divided into 0.5 mg/m^2/dose twice a day , with the goal level being below the level where toxicity may be anticipated (> 15ng/ml). Sirolimus levels will be checked at Day -5. If levels are not toxic on Day -5, will continue maintenance dose and complete pre-dose assessments on Day -6 for dosing the next day. If levels are within toxic levels on Day -5, dose will be adjusted accordingly, and participant will complete seven day regimen per QI's discretion. The half-life of sirolimus is such that 5 days is sufficient to see steady state levels, so levels may be checked starting at that time.

The day before dosing, the following procedures will be done:

- Physical exam
- Vital signs
- Recording of adverse events and concomitant medications
- Main efficacy outcome measures (Ashworth and Tardieu scales)
- Exploratory measures (Vineland, Bayley, log books of seizures and falls, CGI)
- Safety laboratories

6.3. Dosing day:

The following will be done on the day of dosing:

1) Exploratory labs: addition of Elispot and serum cytokines
2) Sirolimus dosing will be 1 mg/m^2/day divided into 0.5 mg/m^2/dose twice a day
3) Administration of additional immunosuppressants and analgesic:
    a. Methyl Prednisolone (IV) - 10 mg/kg to a maximum single dose 500 mg, infused over 30 minutes
    b. Acetaminophen - 15 mg/kg dose (maximum 650 mg/ dose)
    c. Diphenhydramine - 0.5 mg/kg/dose (maximum 50 mg/dose)
4) Lumbar puncture/Intrathecal administration of MELPIDA under anesthesia
    a. Removal of spinal fluid to equal volume of MELPIDA dose
    b. Cerebral-spinal fluid analysis, including cell count, differential, protein, glucose, gram stain, culture.
    c. Administration of MELPIDA (see below)
5) Collection of viral shedding samples (urine, saliva, stool) post dosing

6.3.1. Administration of Study intervention (MELPIDA):

A Pajunk atraumatic Sprotte needle (part number 321151-31A) will be inserted percutaneously at the lumbar level into the intrathecal space of the spinal column. Spinal needle placement will be confirmed using fluoroscopic intraoperative imaging (CArm-) scanner at the chosen injection site prior to and after vector administration.

A volume of CSF approximately equal to the infusion volume (10 ml) will be withdrawn from the lumbar thecal sac. The MELPIDA vector solution will be loaded into a 20 mL BD syringe, connected to the needle with 60 inch mini volume IV extension tubing and a Braun 4-way stopcock. The vector solution is then infused at a rate of 1 mL per minute, using a CareFusion Alaris 8110 syringe pump or similar pump able to deliver at a rate of 1 mL/min.

6.3.2. Anesthesia Safety

The participant will undergo a comprehensive pre-anesthesia evaluation prior to dosing (within two weeks of dosing). Physiologic monitoring in accordance with the standards set by the American Society of Anesthesiologists will be utilized while they are receiving analgesia/anesthesia and until they have fully recovered from its effects. Active warming devices will be used during anesthesia as needed since patients are prone to hypothermia during the anesthesia.

6.3.3. Post-Procedure Recovery

After the procedure, if not conducted in the Pediatric Intensive Care Unit (PICU), the participant will be transported to a Post Anesthesia Care Unit (PACU) or (PICU) with continuous pulse oximetry monitoring and oxygen if needed by bag/mask/nasal canula or blow-by. Vital signs including heart rate, respiratory rate, blood pressure, and pulse oximetry will be monitored every 15 minutes for thefirst 2 hours post infusion, every 30 minutes during the third and fourth hour post infusion, then hourly for 4 hours,and finally every 4 hours until discharge. In the event that abnormalities are detected, appropriate medical intervention will occur, including the possibility of extending the hospitalization and/or subsequent testing. If low oxygen saturation is observed (< 93%), the patient will be evaluated and treated as clinically indicated. This may include, but are not limited to, assessments on physical examination, initiating of supplemental oxygen therapy, measurement of blood oxygen levels by ABG (arterial blood gas), Chest X-ray, CT imaging of the chest, PFTs (pulmonary function tests),or aerosol therapy. In the unlikely event that a severe allergic reaction should occur, the medical and nursing staff will follow the anaphylaxis guidelines.

6.4. Day 2

Vital signs, physical exam, neurologic exam, safety labs and a review of concomitant medications and any adverse events will be assessed prior to discharge. The first dose of tacrolimus at 0.2 mg/kg/day divided into twice daily dosing; 0.1 mg/kg/dose (goal level: 2-15ng/mL) The first dose of prednisone/prednisolone at 1-1.4 mg/kg/day will also be given. When the attending physician determines that the participant is stable, then he may be discharged.

Immunosuppressive medications (prednisone/prednisolone, tacrolimus and sirolimus) will be continued daily starting from Day 2.

6.5. Days 7, 14, 21, 28 (+/- 2 days)

The participant will return to SickKids on Day 7, 14, 21 and 28. Vitals, safety labs, brief physical exam, viral shedding samples, concomitant medications and adverse events will be collected. On Day 7 and 21 exploratory labs will also be done. Immunosuppression with prednisone/prednisolone, tacrolimus and sirolimus will continue daily. Nerve conduction studies will be performed at 21 days.

6.6. Months 3, 6, 9 and 12 (+/- 14 days)

The participant will return to SickKids to repeat the majority of the study procedures (see Schedule of events), including all secondary outcome measures. Brain MRI and lumbar puncture with CSF analysis will be performed at 3, 6, 9, and 12 months (via one coordinated anesthesia, with MRI first). A liver ultrasound will be conducted at 6 and 12 months. Nerve conduction studies will be performed at 3, 6 and 12 months. If at month 3, there is no evidence of inflammation, prednisone/prednisolone tapering may begin at month 4 (see Immune Modulation Protocol Section 6.10). Tacrolimus and sirolimus dosing will continue without tapering. If there is evidence of inflammation at Month 3, prednisone/prednisolone tapering will not commence until after the next examination at Month 6. When there is no evidence of inflammation, then the schedule for tapering may commence. If signs of inflammation continue to be present at 6 months, immunosuppressive medicines will be continued, and MRI and LP will be repeated at 9 months to again assess.

6.7. Months 18, 24, 36, 48, 60 (+/- 14 days)

The month 18 study visit at SickKids will primarily be for safety (physical exam, vitals, height and weight, safety and exploratory labs) and efficacy assessments (spasticity, Bayley IV, and Vineland). Immunosuppression should be in the tapering phase for sirolimus and tacrolimus.

The participant/parents/guardians will be encouraged to contact the investigator for any suspected adverse event reporting between visits. Unscheduled visits may occur if the PI determines that they are necessary to assess safety, repeat labs, etc.

A complete schedule of events is found in Table 2.

Table 2 Schedule of Events

Visit/ Screen¥§ Day	-28 to -8	-7 to -1	-1	1	2	7	14	21	28	3 Month	6 Month	9 Month	12 Month	18 Month	24 Month	36 Month	48 Month	60 Month
Informed Consent	X																	
Genetic confirmation	X																	
Medical History	X																	
Vitals	X		X	X	X	X	X	X	X	X	X	X	X	X	X	X	X	X
Height and Weight	X			X						X	X	X	X	X	X	X	X	X
Physical Exam	X		X	X	X	X	X	X	X	X	X	X	X	X	X	X	X	X
Concomitant Medications §	X		X		X				X	X	X	X	X	X	X	X	X	X
Adverse Events §			X		X	X	X	X	X	X	X	X	X	X	X	X	X	X
Screen Labs*	X																	
Exploratory Labs****			X			X		X		X	X	X	X	X	X	X	X	X
Safety Labs**	X		X		X	X	X	X	X	X	X	X	X	X	X	X	X	X
MELPIDA Dosing				X														
PICU admit for dosing				X	X													
EKG	X			X						X	X	X	X		X	X	X	X
Neurologic Exam	X								X	X	X	X	X		X			
Lumbar Puncture				X						X	X	X	X		X			
CSF Analysis***				X						X	X	X	X		X	X	X	X
MRI	X****									X+	X	X	X		X		X	X
Ultrasound (Liver)	X										X		X		X	X	X	X
NCS	X							X		X	X		X		X	X	X	X
AAV NAb Titers	X		X		X	X	X	X		X	X		X		X	X	X	X
Anti A4PM1											X	X	X	X	X			
Viral shedding				X		X	X	X	X	X	X	X	X					
Immunosuppressants:																		
Prednisone/Prednisolone [10]					X	X	X	X	X	X [11]	X	X	X					
Acetaminophen [5]				X														
Diphenhydramine [6]				X														
Methylprednisolone (IV) [7]				X														
Tacrolimus (rapamycin) [8]					X	X	X	X	X	X	X [9]	X	X	X				
Sirolimus		X[1]	X[1]	X[2]	X[3]	X	X	X	X	X	X	X	X[4]	X				
Sirolimus Levels		X[16]																
Spasticity Assessments [13]	X		X							X	X	X	X	X	X	X	X	X

Bayley Scales of Infant and Toddler Development ed IV	X	X					X	X	X	X	X	X	X	X	X
Vineland Adaptive Behaviour Scale	X	X					X	X	X	X	X	X	X	X	X
Clinical Global Impression of Overall Change by Physician (CGI)	X	X					X	X	X	X	X	X	X	X	X
Log book of seizures [14]	X	X	X	X	X	X	X	X	X	X	X	X	X	X	X
Log book of falls [14]	X	X	X	X	X	X	X	X	X	X	X	X	X	X	X

¥Some visits are in-patient admissions and may last for more than the day of admittance.
§Between study visits there will be biweekly phone calls to assess con meds and AEs.
*Screening labs include HIV Ab, Hepatitis A Ab, Hepatitis B Surface Ab, Hepatitis Surface Ag, Hepatitis C Core Ab, PPD skin test for TB, HTLV1
**Safety labs include CBC With Differential, ESR, CRP, LFT, HCT, MCV, Na, K, Cl, CO_2, BUN, Cr, glucose, ALT, AST, Total biilrubins, Direct.bilirubins, ALP, GGT, Ca, INR, PT, PTT, Urinalysis, EKG, Cardiac Safety (Tn, ProBNP, CK-MB), Sirolimus Levels, Tacrolimus levels, Lipid Profile
***CSF Analysis includes cell count, differential, protein, glucose, gram stain, culture, oligoclonal bands, cytokine analysis.
****Exploratory Tests = AAV9 NAb, Serum Cytokine Analysis & EliSPOT
*****May use previous baseline MRI if within 2 years of dosing
X+ = MRI Brain & Spine With contrasts (at Baseline & 3M only)

1- Sirolimus load 1 mg/m^2 every 4h for 3 doses (load only given on one day), then 1 mg/m^2/day divided in twice a day dosing; 0.5 mg/m^2/dose (toxic level > 15 ng/ml)
2- Sirolimus maintenance dose 0.5 mg/m^2/dose twice a day dosing on day 1 (1 mg/m^2/day)
3- Sirolimus maintenance dose 0.5 mg/m^2/dose twice a day dosing DAILY from day 2 to Month 12 (1 mg/m^2/day)
4- Sirolimus taper may begin starting at Month 12. If any evidence of ongoing inflammation in any exam at Month 12, continue with 0.5 mg/m^2/dose twice a day doses DAILY (1 mg/m^2/day), and do not begin taper until results of next clinical evaluation of inflammation and reassess.
5- Acetaminophen 15 mg/kg dose (max 650 mg/ dose)
6- Diphenhydramine 0.5 mg/kg/dose (max 50 mg/dose)
7- Methylprednisolone (IV) 10 mg/kg to a max single dose 500 mg, infused over 30 minutes
8- Tacrolimus 0.1 mg/kg/dose twice DAILY; 0.2 mg/kg/day (goal level 2-15ng/ml) from day 2 to month 6
9- Tacrolimus - Based on clinical results of LP, MRIs at Month 6, tacrolimus taper may begin at Month 6 (Week 24). If any evidence of ongoing inflammation in any exam at Month 6, continue with 0.1 mg/kg/dose twice DAILY (0.2 mg/kg/day); do not begin taper until results of next clinical evaluation of inflammation and reassess
10- Prednisone/Prednisolone 1-1.4 mg/kg/day DAILY until the 3 month visit
11- Prednisone/Prednisolone - Based on clinical results of LP and MRIs at Month 3, prednisone/prednisolone taper may begin after this 3 month study visit. Criteria for tapering include no evidence of inflammation in CSF (based on LP) or evidence of brain or spine inflammation (MRI). If any evidence of ongoing inflammation in any exam at Month 3, continue with 1-1.4 mg/kg/day DAILY; do not begin taper until results of next clinical evaluation of inflammation and reassess
12- If inflammation evident at month 6, repeat MRI, LP and CSF analysis at month 9
13- Modified Ashworth and Tardieu scales to assess spasticity
14- Starting at screening, the number of falls and seizures will be recorded daily
15- Blood draw will take into account body weight, daily and 30 day reference maximums
16- Lab for Sirolimus levels on Day -5

6.8. Specific Study Procedures

6.8.1. Nerve conduction study (NCS)

The participant will undergo nerve conduction studies to evaluate for peripheral nerve injury. A baseline study will be performed within 28 days of dosing. Standard methodology, as used in routine clinical care, will be used. At least two sensory and one motor nerve in the upper and lower extremity will be sampled. Nerves to be tested (Sensory action potentials (SAP); Sural, peroneal superficialis, median and ulnar. Compound Muscle action Potentials (CMAP); Tibial, Median) Studies should be always done on the same side each time

Sensory action potentials (SAP) will be recorded with an orthodromic procedure for median, and ulnar nerves, antidromically in the superficial peroneal and sural nerves. Motor distal latencies, compound muscle action potential and minimal F-wave latencies will be recorded for median, and tibial nerves. No EMG will be done unless we observe an axonal neuropathy.

NCVs will be done at baseline, day 21, 3 month, 6 month, 12 month,24 monh, 48 month and 60 month after dosing.

6.8.2. Magnetic Resonance Imaging of Brain (MRI)

Brain MRI (with and without contrast) will be done at baseline and at 3 month intervals. Standard imaging sequences will be obtained (T1, T2, FLAIR, etc). MRI studies will be done at The Hospital for Sick Children. The primary role for brain MRI in the study is to evaluate for signs of inflammation and inflammatory change associated with the study drug. Of note, patients with SPG50 typically have structural brain abnormalities. On MRI, the following changes have been observed in SPG50 patients: (1) thin splenium of the corpus callosum, (2) absent or thin anterior commissure, (3) characteristic signal abnormalities of the forceps minor (“ears of the grizzly sign”), and (4) periventricular white matter. All MR imaging will be supervised by an attending pediatric neuroradiologist to ensure acquisition of complete, high quality scans.

6.8.3. Liver Ultrasound

A liver ultrasound will be conducted at pre-dose, Months 6 and 12 and then yearly. Liver imaging studies will be done at the Hospital for Sick Children. The primary role for the liver ultrasound to is monitor signs of liver toxicity associated with the study drug. All ultrasound imaging will be supervised by an attending pediatric neuroradiologist to ensure acquisition of complete, high quality scans.

6.8.4. Lumbar puncture

The participant will undergo one lumbar puncture via interventional radiology guidance. The participant will undergo additional post-dosing lumbar punctures as performed without IR

guidance by the study PI. For all LPs, a 21 gauge standard LP needle will be used. The area around lumbar 4/5 will be sterilely addressed. EMLA will be applied for local anesthesia. The needle will be inserted into the inter thecal space between L4/L5. An appropriate quantity of CSF will be removed for relevant laboratory studies. The needle will be removed, the site cleaned, and a sterile dressing will be applied.

6.9. Adverse Event and Concomitant Medication Monitoring

The participant's caregivers are encouraged to contact the study team whenever an adverse event or change in medication occurs in a timely manner. Long term adverse event monitoring will include annual study visits at SickKids.

6.10. Immune Modulation Protocol

In previous gene therapy studies, antigen specific T-cell responses to the AAV9 vector have been reported (Harrison et al, 1977). This is an expected response between 2- and 12-weeks following gene transfer, even when administered IT. One possible consequence to such antigen specific T-cell responses is clearance of the transduced cells and loss of transgene expression.

To reduce the risk of the host immune response to the AAV9-based MELPIDA, an initial proposal for an immunosuppression regimen has been designed based on advice from investigators in the ongoing trial of AAV9 gene transfer to CSF for giant axonal neuropathy (Clinicaltrials.gov # NCT02362438).

<u>1 Week Prior to Vector Administration:</u>

- Sirolimus load: 1 mg/m2 every 4 hours x 3 doses (load only given on one day)
- Starting the day after the sirolimus load, begin enteral daily dosing at 1 mg/m2/day, divided in twice per day dosing, 0.5mg/m2/dose (toxic level: >15 ng/mL)
- Sirolimus levels will be checked at Day -5
  - If levels are not toxic on Day -5, will continue maintenance dose and complete pre-dose assessments on Day -6 for dosing the next day.

If levels are toxic on Day -5, dose will be adjusted accordingly and participant will complete seven day regimen, per QI's discretion.

<u>Day of Vector Administration (Day 1):</u>

- Acetaminophen (15 mg/kg/dose enteral; maximum 650 mg per dose)
- Diphenhydramine (0.5 mg/kg/dose enteral; maximum 50 mg/dose)
- IV methylprednisolone (10 mg/kg to a maximum single dose of 500 milligrams, infused over 30 minutes)

<u>Day after Vector Administration (Day 2):</u>

- Begin daily enteral prednisone/prednisolone at 1-1.4 mg/kg/day x 3 months
- Continue enteral daily sirolimus dosing at 1mg/m2/day, divided in twice per day dosing, 0.5 mg/m2/dose (toxic level: >15ng/mL)

- Tacrolimus at 0.2 mg/kg/day divided into twice daily dosing; 0.1 mg/kg/dose (goal level: 2-15 ng/mL)

Maintenance:
- Enteral prednisone/prednisolone at 1-1.4 mg/kg/day x 3 months, then taper according to schedule
- Sirolimus 1 mg/m2/day divided in twice per day dosing; 0.5mg/m2/dose (toxic level: >15 ng/mL). If there are signs or symptoms of transgene mediated CNS inflammation by examination, brain imaging, and/or laboratory testing, longer administration of immunomodulatory medications and possibly addition of other immunomodulatory agents may be required
- Tacrolimus at 0.2 mg/kg/day divided into twice daily dosing; 0.1 mg/kg/dose (goal level: 2-15ng/mL); tacrolimus will be continued for 6 months and will begin taper by 7 months after gene transfer. The taper will be started if there are no signs or symptoms of transgene mediated CNS inflammation by examination, brain imaging, and/or laboratory testing, which if present may require longer administration of immunomodulatory medications

Monitoring:
- Weekly BP checks x 4 weeks
- Sirolimus troughs every 1 week x 4, then every 3 months x 4, then every 6 months x 2 and then annually x 3
- Tacrolimus troughs every 1 week x 4, then every 3 months x 4, then every 6 months x 2 and then annually x 2
- CBC with differential testing at every blood draw
- Monthly fasting lipid profile while on immunomodulation and at PI's discretion

Tapering:

- Prednisone/prednisolone may be tapered starting after the 3 month visit. Taper will occur over 4 weeks. Dose will be decreased to approximately 75% of maintenance in taper week 1 (approximately = most convenient dosing, given options related to formulation, that is about a 25% reduction). Dose will be decreased to approximately 50% of maintenance for taper week 2. Dose will be decreased to approximately 25% of maintenance for taper week 3. Medicine will be stopped after week 3.

- Tacrolimus may be tapered beginning after the 6 month visit. The medicine will be decreased by 0.05 weekly (so week 1 of taper will be 0.1 mg/kg for AM dose and 0.05 mg/kg for PM dose, week 2 of the taper will be 0.05 mg/kg/dose, week 3 will be 0.05 mg/kg in AM and 0.025 in PM, week 4 0.025 mg/kg/dose, week 5 stop)

- Sirolimus may be tapered beginning after the 12 month visit. It will be tapered similarly to tacrolimus.

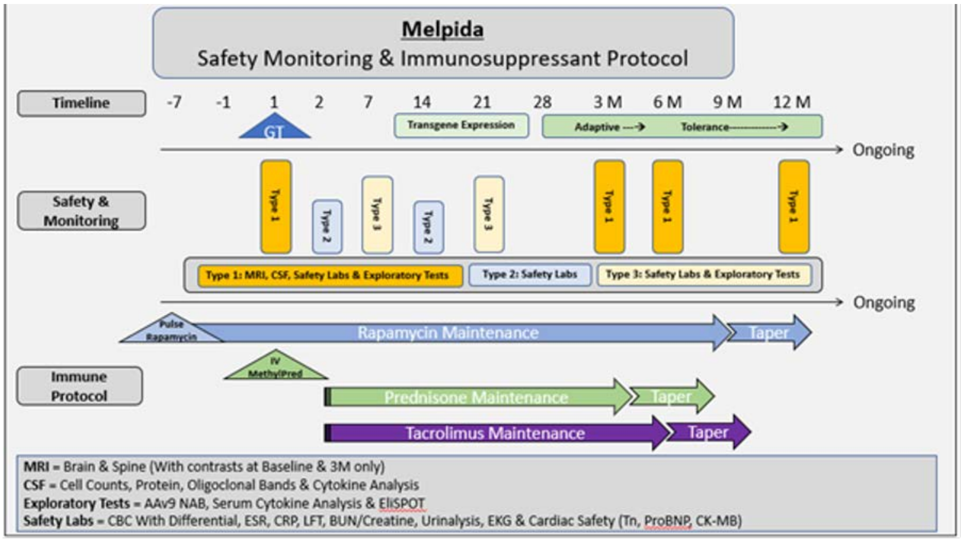

7. INCLUSION/EXCLUSION CRITERIA

7.1. Inclusion Criteria

- Age <5 years old
- Confirmed diagnosis of SPG50 disease by:
  - Genomic DNA mutation analysis demonstrating homozygous or compound heterozygous, pathogenic and/or potentially pathogenic variants in the AP4M1 gene
  - Clinical history or examination features consistent with SPG50 and that include neurologic dysfunction

- Parent/legal guardian willing to accompany the participant to all study visits and who will provide permission for their child's participation.

7.2. Exclusion Criteria

- Inability to participate in the clinical evaluation
- Presence of a concomitant medical condition that precludes lumbar puncture or use of anesthetics
- Bleeding disorder or any other medical condition or circumstance in which a lumbar puncture is contraindicated according to local institutional policy
- Inability to be safely sedated in the opinion of the clinical anesthesiologist
- Active infection based on clinical observations

- Concomitant illness or requirement for chronic drug treatment that in the opinion of the PI creates unnecessary risks for gene transfer
- Any item which would exclude the patient from being able to undergo MRI according to local institutional policy
- Any other situation that would exclude the patient from undergoing any other procedure required in this study
- The presence of significant non-SPG50 related CNS impairment or behavioral disturbances that would confound the scientific rigor or interpretation of results of the study
- Have received an investigational drug within 30 days prior to screening or plan to receive an investigational drug (other than gene therapy) during the study.
- Enrollment and participation in another interventional clinical trial
- Contraindication to MELPIDA or any of its ingredients
- Contraindication to any of the immune suppression medications used in this study
- Clinically significant abnormal laboratory values (GGT, ALT, and AST, or total bilirubin > 2 × ULN, creatinine ≥ 1.0 mg/dL, hemoglobin [Hgb] < 8 or > 18 g/dL; white blood cell [WBC] > 20,000 per cmm) prior to gene replacement therapy. Patients with an elevated bilirubin level that is unequivocally the result of neonatal jaundice shall not be excluded

7.3. Participant Withdrawal

The participant/parents/guardians will be consented prior to their enrollment in the study. They will be made aware that participation in the study is voluntary and they can withdraw at any time. Participants are free to withdraw from participation in the study at any time upon request. If possible, the study site should attempt to have the participant return for one last study visit (for t = 36 month study visit procedures).

Early withdrawal may also occur for any of the following reasons:
1. Protocol deviation (at the Investigator’s discretion)
2. Investigator discretion
3. Study termination by Investigator
4. Lost to follow up
5. Participant enrollment in a different interventional study for SPG50

An investigator may discontinue or withdraw a participant from the study for the following reasons:
- If any clinical adverse event (AE), laboratory abnormality, or other medical condition or situation occurs such that continued participation in the study would not be in the best interest of the participant
- Disease progression which requires discontinuation of the study intervention
- If the participant meets an exclusion criterion (either newly developed or not previously recognized) that precludes further study participation

Follow-up Procedures following Withdrawal
For the safety of the participant, every effort will be made for a final evaluation (per t = 36 month study visit). This includes follow-up for any unresolved adverse events.

8. STATISTICAL ANALYSIS

Data collected from this study will be presented in aggregate. Rates of adverse events and serious adverse events will be reported by category. Given that this is an N of 1 trial, analytic statistics at a population level will not be possible. Descriptive statistics will be provided. Furthermore, rates of change in disability scores will beprovided, but the study is not powered to discern the effect size of the therapy.

8.1. Data Monitoring

8.1.1. General Plan

For this Phase I study of Gene Therapy the safety oversight will be focused not only on the initial treatment but also on an extended observation.

8.1.2. Monitoring Entity

Dr. James Dowling (SickKids Toronto) is the lead investigator and will be responsible for assuring ongoing safety monitoring of the trial. All medical decisions will be made in the best interest of the participant. Management of toxicities will be at the discretion of the lead investigator in consultation with experts in the field.

8.2. Plans for Assuring Participant Safety, Adverse Event Collection, and Reporting

Expected adverse events could be due to the infusion and are listed above. Other adverse events could be due to immunomodulatory drugs or to an inflammatory response to the AAV.

Maximum efforts will be undertaken to ensure the safety of study participant as per the requirements of HC and SickKids REB.

The primary and secondary endpoint assessment is identifying the safety and tolerability of intrathecal administration of MELPIDA. Monitoring for safety will be performed by recording and evaluating type and occurrences of Adverse Events (AEs), concomitant medication usage, and by conducting physical examinations, vital sign assessments, cardiovascular evaluations, and laboratory evaluations (chemistry, hematology, coagulation,immunology).

8.3. Definitions

8.3.1. Adverse Event

An Adverse Event (AE) is any untoward medical occurrence associated with the use of an intervention in a study participant, which does not necessarily have a causal relationship with the intervention. An AE can therefore be any unfavourable and unintended sign (including an abnormal laboratory finding), symptom or disease temporally associated with the use of the intervention, whether or not considered related to the investigational intervention.

Stable chronic conditions which are present prior to entry in the study and do not worsen are not considered AE. These pre-existing conditions will be documented in the participant's medical history.

These events will be reviewed by the PI and determined if they are clinically significant requiring adjustments to medications or interventions as per HC regulations. Reporting to HC and REB are outlined in Section 8.5, Section 8.6 and Section 8.7.

Clinically significant signs and symptoms or lab abnormalities will be recorded as an AE. This could include a laboratory result for which there is no intervention, but the abnormal value suggests a disease or organ toxicity. The PI will evaluate all AEs with respect to Seriousness, Severity (intensity or grade), and Causality (relationship to study agent and relationship to research) according to the following guidelines. All AEs will be classified in accordance with the CTCAE v.5. AEs will be coded in accordance with the most current version of the MedDRA coding dictionary.

.

8.3.2. Classification of Adverse Events

Monitoring AEs requires that they be classified as to seriousness, expectedness, and potential relationship to the investigational product, all of which drive the reporting process.

8.3.2.1. Seriousness

A serious adverse event (SAE) is one that:

- Results in death,
- Is life-threatening (the participant was in immediate danger of death from the event as it occurred),
- Requires inpatient hospitalization or prolongation of existing hospitalization,
- Results in persistent or significant disability/incapacity, or
- Is a congenital anomaly/birth defect in the offspring of a participant.

All SAEs that occur after any patient has been enrolled, before vector dosing, during vector dosing, or up through the last study visit, whether or not they are related to the study, must be recorded on case report forms.

CTCAE v.5 provides a grading system that is used to categorize the severity of adverse events, as follows:

- Grade 1 Mild: transient, requires no special treatment or intervention, does not interfere with daily activities
- Grade 2 Moderate: alleviated with simple treatments, may limit daily activities
- Grade 3 Severe: requires therapeutic intervention and interrupts daily activities
- Grade 4 Life-threatening or disabling
- Grade 5 Death

An SAE, as defined above, encompasses CTCAE grades 4 and 5, and any Grade 3 event that requires or prolongs hospitalization, or that is disabling. Other SAEs that are considered Important Medical Events (IME) requiring medical judgement that need reporting is when the event does not fit the outcomes listed above, but the event mayjeopardize the patient and may require medical or surgical intervention (treatment) to prevent one of the other outcomes.

8.3.2.2. Expectedness

The purpose of reporting is to provide new, important information on serious reactions or events previously unobserved or undocumented. Therefore, all AEs will be evaluated as to the expectedness of its occurrence as follows:

- Unexpected: An unexpected AE or adverse drug reaction is one for which the nature or severity is not consistent with information in the protocol, consent form, or Investigator’s brochure.
- Expected: While there remains limited data on the safety of AAV9 and AAV based gene therapy programs in general, some AEs have emerged in across different programs. Ones that have been noted in multiple individuals and in more than one patient include:
    a. Transient thrombocytopenia
    b. Transient transaminitis
- An AE is considered expected if it is known to be associated with any of the study procedures (i.e., blood draw sticks, LPs, etc)
- Expected adverse events <u>due to underlying disease</u> are listed below:
    - Worsening of spasticity
    - Worsening of ataxia
    - Worsening seizures
    - Progressive atrophy in brain noted in follow-up MRIs

8.3.2.3. Causality

Causality assessment is required in clinical investigations to help determine which events require expedited reporting. The PI must make the determination of relationship to the investigational product for each AE (Unrelated,Possibly Related, Probably Related, or Definitely Related). The PI should decide whether, in his/her medical judgment, there is a reasonable possibility that the event may have been caused by the investigational product. If no valid reason exists for suggesting a relationship, then the AE should be classified as “unrelated.” If there is anyvalid reason, even if undetermined, for suspecting a possible causative relationship between the investigational product and the occurrence of the AE, then the AE should be considered “related.” If the relationship between the AE/SAE and the investigational product is determined to be “possible” or “probable”, the event will be considered to be related to the investigational product for the purposes of expedited regulatory reporting.

The following criteria will be used to determine causality:

- Unrelated: The event is clearly related to other factors, such as the participant’s clinical state or non-study drugs or interventions.
- Possibly Related: The event follows a compatible temporal sequence from the time of administration of the study agent, but could have been produced by other factors such as the participant’s clinical state or non-studydrugs or interventions.
- Probably Related: The event follows a reasonable temporal sequence from the time of study agent administration, and cannot be reasonably explained by other factors such as the participant’s clinical state or non-study drugs or interventions.

8.4. Dose Limiting Toxicity

Dose limiting toxicity (DLT) is defined as any SAE or AE that is possibly, probably, or definitely related to the investigational product. This would include any AE Grade 3 or greater event, according to the CTCAE v.5; these classificationsare outlined below:

- Grade 1 Mild: transient, requires no special treatment or intervention, does not interfere with daily activities
- Grade 2 Moderate: alleviated with simple treatments, may limit daily activities
- Grade 3 Severe: requires therapeutic intervention and interrupts daily activities
- Grade 4 Life-threatening or disabling
- Grade 5 Death

Other Adverse Events

Other adverse events (OAEs) may be identified by the PI. Significant AEs of particular clinical importance, other than SAEs and those AEs leading to discontinuation of the participant from the study, will be classified as OAEs.

8.5. Reporting Procedures to the REB

The REB will have access to review of participant data during the course of the study through access to the participant's eCRFs. All SAEs will also be reported to the REB. Requests for additional data can be made by communicating the request to the PI.

8.6. Reporting Procedures to Health Canada

As the PI is the sponsor-Investigator, the PI will notify HC of potential serious risks, from clinical trials or any other source, as soon as possible. For fatal or life threatening events, the PI will report to HC with 7 days and when neither fatal or life threatening, they will report within 15 days, as per HC regulations.

Information that qualifies reporting:

- *Serious and unexpected suspected adverse reaction.* The PI will report any suspected adverse reaction thatis both serious and unexpected. The PI will report an adverse event as a suspected adverse reaction only if there is evidence to suggest a causal relationship between the drug and the adverse event, such as
    - single occurrence of an event that is uncommon and known to be strongly associated with drug exposure.
    - One or more occurrences of an event that is not commonly associated with drug exposure, but is otherwise uncommon in the population exposed to the drug.
    - An aggregate analysis of specific events observed in a clinical trial (such as known consequences of the underlying disease or condition under investigation or other events that commonly occur in the study population independent of drug therapy) that indicates those events occur more frequentlyfollowing treatment.

- *Findings from other studies.* The PI will report any findings from epidemiological studies, pooled analysisof multiple studies, or clinical studies that suggest a significant risk in humans exposed to the drug.
- *Findings from animal or in vitro testing.* The PI will report any findings from animal or in vitro testing, whether or not conducted by the sponsor, that suggest a significant risk in humans exposed to the drug.
- *Increased rate of occurrence of serious suspected adverse reactions.* The sponsor must report anyclinically important increase in the rate of a serious suspected adverse reaction over that listed in the protocol or investigator brochure.
- *Submission of CTA safety reports.* The PI will submit each CTA safety report in a narrative format or in an electronic format that HC can process, review, and archive. Reports of overall

findings or pooled analyses from published and unpublished in vitro, animal, epidemiological, or clinical studies must be submitted in a narrative format.

- *Unexpected fatal or life-threatening suspected adverse reaction reports.* The PI will also notify HC of any unexpected fatal or life-threatening suspected adverse reaction as soon as possible but in no case laterthan 7 calendar days after the PI's initial receipt of the information.

8.7. Reporting Procedures to the Research Ethics Board

Suspected unexpected serious adverse reactions (SUSARs) will be reported to the REB within 5 workingdays of discovery if they follow the following definition:

An event that meets ALL three (3) of the following criteria:

- Unexpected (in nature, severity, or frequency), AND
- Probably or definitely related to participation in research, AND
- Suggests the investigational product places subjects or others at a greater risk of harm than previously known or recognized.

All other research-related events and reports will be summarized at annual continuing review (AR) or notice of study closure, whichever comes first. That includes, but is not limited to:

- Noncompliance events (e.g., deviations)that do not meet the SickKids REB definition of either serious or continuing noncompliance
- AEs/SAEs that do notmeet ALL 3 SUSAR criteria
- Events/reports the sponsor wants submitted to the SickKids REB
- Other safety reports
- Monitoring/audit reports
- Any other new information since the last REB review

A Summary Report of AEs will be prepared by the PI annually and will be sent to the REB at continuing review. The Summary Report will contain the following information:

- A statement as to whether or not the frequency of AEs exceeded what was expected and indicated in the informed consent.
- A statement that if safety concerns are identified, they will be communicated promptly to the investigators.

8.8. Protocol Deviations and Continuing Review

Protocol deviations and unanticipated problems will be reported to the SickKids REB as per their reporting guidelines.

The following items will be reported to the SickKids REB in summary at the time of Continuing Review:

- Serious and non-serious unanticipated problems,
- Expected serious adverse events that are possibly, probably, or definitely related to the investigational product,
- Serious adverse events that are not related to the investigational product
- All adverse events, except expected AEs and death granted a waiver of reporting,
- Any trends or events which in the opinion of the investigator should be reported, and
- Any protocol-specific reporting requirements (as applicable).

8.9. Stopping Rules

Not applicable for this single participant study.

9. DATA COLLECTION

Data will be collected at specified time intervals as outlined in the protocol. Once the participant and/or parent/guardian has signed the informed consent/assent form, data can then be collected, including pertinent retrospective medical records per PI discretion.

Source data is all information, original records of clinical findings, observations, or other activities in a clinical trialnecessary for the reconstruction and evaluation of the trial. Source data are contained in source documents. Examples of these original documents, and data records include: hospital records, clinical and office charts, pathology reports, laboratory notes, memoranda, participants' diaries or evaluation checklists, pharmacy dispensingrecords, recorded data from automated instruments, copies or transcriptions certified after verification as being accurate and complete, microfiches, photographic negatives, microfilm or magnetic media, digitized imaging data,x-rays, participant files, and records kept at the pharmacy, at the laboratories, and at medico-technical departmentsinvolved in the clinical trial.

The study electronic case report forms (eCRF) is where all data collection will be inputted for the study. All data requested on the eCRF will be recorded by the clinical operations team consisting of the clinical research coordinators/managers and research nurses. The electronic data capture (EDC) platform used in this clinical trial will be REDCap. It supports regulatory trials to ensure Good Clinical Practice (GCP). All missing data must be explained, and it will have automatic data verification in place to ensure complete and accurate data is entered. REDCap will be

accessed via a secure personalized login, thus allowing for role-appropriate access, and providing audit trails for data entry,exports, and reports. Trial data will be entered using the participant identification number. PHI will not be shared outside of REB approved entities.

De-identified data may be transferred to support regulatory submissions for purposes relating to MELPIDA (study drug).

AAV8 Nab, anti AP4M1, Serum Cytokine Analysis & EliSPOT will be sent to an external laboratory for analysis. Saliva, urine and stool samples will be collected to assess viral shedding. These samples will be sent and analyzed at an external lab.

9.1. Database Locks

For key deliverables requiring analysis of the trial data, an export of the entire database from REDCap will be performed at such periodic intervals in order to have a locked dataset from which all results will be generated. At trial end, a final lock and export will occur after all data queries are resolved and statistical analysis will be performed. The final trial results and publications will be prepared from this locked dataset.

9.2. Study Monitoring Plan

This study will be monitored according to the outline in the protocol. The PI will allocate adequate time for such monitoring activities. The data entered into REDCap will be reviewed and verified for accuracy by the Investigator.

SickKids Hospital Research Clinical Research Quality and Education (CRQE) will also provide trial monitoring. The ongoing data monitoring responsibilities are performed by the SickKids Hospital PI, Dr. Dowling, and the CRQE, to monitor the study progress and will function independently from the study team. The PI will also ensurethat SickKids Hospital REB and CRQE or other compliance/quality assurance reviewers are given access to all the above noted study-related documents and study-related facilities and has adequate space to conduct monitoring visits. The PI will permit study-related monitoring, audits, and inspections by the government regulatory bodies such as the HC.

9.3. Quality Assurance of Data

Quality assurance (QA) processes are in place to ensure the data will be collected and entered into the EDC accurately and consistently.

10. INSTRUMENTS FOR THE ASSESSMENT OF DISEASE

10.1. Modified Ashworth Scale

Modified Ashworth Scale Instructions

General Information (derived Bohannon and Smith, 1987):

- Place the patient in a supine position
- If testing a muscle that primarily flexes a joint, place the joint in a maximally flexed position and move to a position of maximal extension over one second (count "one thousand one")
- If testing a muscle that primarily extends a joint, place the joint in a maximally extended position and move to a position of maximal flexion over one second (count "one thousand one")
- Score based on the classification below

Scoring (taken from Bohannon and Smith, 1987):

0	No increase in muscle tone
1	Slight increase in muscle tone, manifested by a catch and release or by minimal resistance at the end of the range of motion when the affected part(s) is moved in flexion or extension
1+	Slight increase in muscle tone, manifested by a catch, followed by minimal resistance throughout the remainder (less than half) of the ROM
2	More marked increase in muscle tone through most of the ROM, but affected part(s) easily moved
3	Considerable increase in muscle tone, passive movement difficult
4	Affected part(s) rigid in flexion or extension

10.2. Tardieu Scale

TARDIEU SCALE

This scale quantifies muscle spasticity by assessing the response of the muscle to stretch applied at specified velocities.

Grading is always performed at the same time of day, in a constant position of the body for a given limb. For each muscle group, reaction to stretch is rated at a specified stretch velocity with 2 parameters x and y.

Velocity to stretch (V)

**V1**	As slow as possible
**V2**	Speed of the limb segment falling
**V3**	As fast as possible (> natural drop)

V1 is used to measure the passive range of Motion. (PROM). Only V2 and V3 are used to rate spasticity

Quality of muscle reaction (X)

**0**	No resistance throughout passive movement
**1**	Slight resistance throughout, with no clear catch at a precise angle
**2**	Clear catch at a precise angle, followed by release
**3**	Fatigable clonus (<10secs) occurring at a precise angle
**4**	Unfatigable clonus (>10secs) occurring at a precise angle
5.	Joint Immobile

Angle of muscle reaction (Y)

Measure relative to the position of minimal stretch of the muscle (corresponding at angle)

Spasticity Angle

**R1**	Angle of catch seen at Velocity V2 or V3
**R2**	Full range of motion achieved when muscle is at rest and tested at V1 velocity

Boyd, Graham 1999

- A large difference between R1 & R2 values in the outer to middle range of normal m. length indicates a large dynamic component
- A small difference in the R1 & R2 measurement in the middle to inner range indicates predominantly fixed contracture

Testing Positions

Upper Limb

To be tested in a sitting position, elbow flexed by 90° at the recommended joint positions and velocities.

**Shoulder**	Horizontal Adductors	V3	
	Vertical Adductors	V3	
	Internal Rotators	V3	
**Elbow**	Flexors	V2	Shoulder adducted
	Extensors	V3	Shoulder abducted
	Pronators	V3	Shoulder adducted
	Supinators	V3	Shoulder adducted
**Wrist**	Flexors	V3	
	Extensors	V3	
	Fingers		Angle PII of digit III- MCP
	Palmar Interossei + FDS	V3	Wrist resting position

Lower Limb

To be tested in supine position, at recommended joint positions and velocities

**Hip**	Extensors	V3	Knee extended
	Adductors	V3	Knee extended
	External Rotators	V3	Knee flexed by 90
	Internal Rotators	V3	Knee flexed by 90
**Knee**	Extensors	V2	Hip flexed by 30
	Flexors	V3	Hip flexed
**Ankle**	Plantarflexors	V3	Knee flexed by 30

11. PRIVACY AND CONFIDENTIALITY

Participant confidentiality and privacy is strictly held in trust by the participating Investigators, their staff, and the Sponsor(s). This confidentiality is extended to cover testing of biological samples and relevant genetic tests in addition to the clinical information relating to participant. Therefore, the study protocol, documentation, data, and all other information generated will be held in strict confidence.

All research activities will be conducted in as private a setting as possible.

Any research information obtained about the patient in this study will be kept confidential. A patient will not be identified by name, only by unique study ID number. The patient’s name or any identifying information will not appear in any reports published as a result of this study. All identifying information will be kept behind 2 security measures or as per equivalent institutional policy, under the supervision of the study/site PI and will not be transferred outside of the hospital.

The study monitor, auditor and other authorized representatives of the Sponsor, representatives of the Research Ethics Board (REB) or HC may inspect all documents and records required to be maintained by the Investigator, including but not limited to, medical records and pharmacy records for the participant in this study. The clinical study site will permit access to such records.

Study participant research data, which is for purposes of statistical analysis and scientific reporting, will be transmitted to and stored in REDcap. This will not include the participant’s contact or identifying information. Rather, the participant and their research data will be identified by a unique study identification number. The study data entry and study management systems used by SickKids research staff will be secured and password protected. At the end of the study, all study databases will be de-identified and archived.

12. REFERENCES

Aguilar RC, Boehm M, Gorshkova I, et al. Signal-binding specificity of the mu4 subunit of the adaptor protein complex AP-4. J Biol Chem. 2001;276(16):13145-13152. doi:10.1074/jbc.M010591200

Bailey RM, Armao D, Nagabhushan Kalburgi S, Gray SJ. Development of Intrathecal AAV9 Gene Therapy for Giant Axonal Neuropathy. Mol Ther Methods Clin Dev. 2018;9:160-171. Published 2018 Feb 15. doi:10.1016/j.omtm.2018.02.005

Behne R, Teinert J, Wimmer M, et al. Adaptor protein complex 4 deficiency: a paradigm of childhood-onset hereditary spastic paraplegia caused by defective protein trafficking. Hum Mol Genet. 2020;29(2):320-334. doi:10.1093/hmg/ddz310

Bohannon RW, Smith MB. Interrater reliability of a modified Ashworth scale of muscle spasticity. Phys Ther. 1987 Feb;67(2):206-7. doi: 10.1093/ptj/67.2.206. PMID: 3809245.

Bradbury AM, Bagel JH, Nguyen D, et al. Krabbe disease successfully treated via monotherapy of intrathecal gene therapy. J Clin Invest. 2020;130(9):4906-4920. doi:10.1172/JCI133953

Bucher T, Colle MA, Wakeling E, Dubreil L, Fyfe J, Briot-Nivard D, Maquigneau M, Raoul S, Cherel Y, Astord S, Duque S, Marais T, Voit T, Moullier P, Barkats M and Joussemet B (2013) scAAV9 Intracisternal Delivery Results in Efficient Gene Transfer to the Central Nervous System of a Feline Model of Motor Neuron Disease. Hum Gene Ther 24:670-82. doi: 10.1089/hum.2012.218

Burgos PV, Mardones GA, Rojas AL, et al. Sorting of the Alzheimer's disease amyloid precursor protein mediated by the AP-4 complex. Dev Cell. 2010;18(3):425-436. doi:10.1016/j.devcel.2010.01.015

Caviness VS, Kennedy DN, Richelme C, Rademacher J, Filipek PA. The human brain age 7-11 years: a volumetric analysis based on magnetic resonance images. Cereb Cortex 1996;6(5):726-36 doi: 10.1093/cercor/6.5.726.

Chen X, Snanoudj-Verber S, Pollard L, et al. Pre-clinical Gene Therapy with AAV9/AGA in Aspartylglucosaminuria Mice Provides Evidence for Clinical Translation. Mol Ther. 2021;29(3):989-1000. doi:10.1016/j.ymthe.2020.11.012

Choudhury SR, Hudry E, Maguire CA, Sena-Esteves M, Breakefield XO and Grandi P (2017). Viral vectors for therapy of neurologic diseases. Neuropharmacology120:63-80. doi: 10.1016/j.neuropharm.2016.02.013

Davies AK, Itzhak DN, Edgar JR, et al. AP-4 vesicles contribute to spatial control of autophagy via RUSC-dependent peripheral delivery of ATG9A. Nat Commun. 2018;9(1):3958. Published 2018 Sep 27. doi:10.1038/s41467-018-06172-7.

De Pace R, Skirzewski M, Damme M, et al. Altered distribution of ATG9A and accumulation of axonal aggregates in neurons from a mouse model of AP-4 deficiency syndrome. PLoS Genet. 2018;14(4):e1007363. Published 2018 Apr 26. doi:10.1371/journal.pgen.1007363.

Dekaban AS. Changes in brain weights during the span of human life: relation of brain weights to body heights and body weights. Ann Neurol 1978;4(4):345-56 doi: 10.1002/ana.410040410.

Ebrahimi-Fakhari D, Behne R, Davies AK, Hirst J. AP-4-Associated Hereditary Spastic Paraplegia. In: Adam MP, Ardinger HH, Pagon RA, et al., eds. GeneReviews®. Seattle (WA): University of Washington, Seattle; 1993, updated 2018.

Ebrahimi-Fakhari D, Teinert J, Behne R, et al. Defining the clinical, molecular and imaging spectrum of adaptor protein complex 4-associated hereditary spastic paraplegia. Brain. 2020;143(10):2929-2944. doi:10.1093/brain/awz307

Gray SJ, Matagne V, Bachaboina L, Yadav S, Ojeda SR and Samulski RJ (2011) Preclinical Differences of Intravascular AAV9 Delivery to Neurons and Glia: A Comparative Study of Adult Mice and Nonhuman Primates. Mol Ther. (6):1058-1069.

Gray SJ. Timing of Gene Therapy Interventions: The Earlier, the Better. Mol Ther. 2016;24(6):1017-1018. doi:10.1038/mt.2016.20

Harrison T, Graham F, Williams J (1977). Host-range mutants of adenovirus type 5 defective for growth in HeLa cells. Virology, 77:319-329.

Haurigot V, Marco S, Ribera A, Garcia M, Ruzo A, Villacampa P, Ayuso E, Anor S, Andaluz A, Pineda M, Garcia-Fructuoso G, Molas M, Maggioni L, Munoz S, Motas S, Ruberte J, Mingozzi F, Pumarola M and Bosch F (2013) Whole body correction of mucopolysaccharidosis IIIA by intracerebrospinal fluid gene therapy. J Clin Invest. doi: 10.1172/JCI66778

Hirst J, Irving C, Borner GH. Adaptor protein complexes AP-4 and AP-5: new players in endosomal trafficking and progressive spastic paraplegia. Traffic. 2013;14(2):153-164. doi:10.1111/tra.12028

Ivankovic D, Drew J, Lesept F, et al. Axonal autophagosome maturation defect through failure of ATG9A sorting underpins pathology in AP-4 deficiency syndrome. Autophagy. 2020;16(3):391-407. doi:10.1080/15548627.2019.1615302

Jameel M, Klar J, Tariq M, et al. A novel AP4M1 mutation in autosomal recessive cerebral palsy syndrome and clinical expansion of AP-4 deficiency. BMC Med Genet. 2014;15:133. Published 2014 Dec 14. doi:10.1186/s12881-014-0133-2

Manns MP, Czaja AJ, Gorham JD, et al. Diagnosis and management of autoimmune hepatitis. Hepatology. 2010;51(6):2193-2213. doi:10.1002/hep.23584

Markakis EA, Vives KP, Bober J, Leichtle S, Leranth C, Beecham J, Elsworth JD, Roth RH, Samulski RJ and Redmond DE, Jr. (2010) Comparative transduction efficiency of AAV vector serotypes 1-6 in the substantia nigra and striatum of the primate brain. Mol Ther 18:588-93.

Masamizu Y, Okada T, Kawasaki K, et al. Local and retrograde gene transfer into primate neuronal pathways via adeno-associated virus serotype 8 and 9. Neuroscience. 2011;193:249-258. doi:10.1016/j.neuroscience.2011.06.080.

Matsuda S, Miura E, Matsuda K, et al. Accumulation of AMPA receptors in autophagosomes in neuronal axons lacking adaptor protein AP-4. Neuron. 2008;57(5):730-745. doi:10.1016/j.neuron.2008.02.012

Mattera R, Park SY, De Pace R, Guardia CM, Bonifacino JS. AP-4 mediates export of ATG9A from the trans-Golgi network to promote autophagosome formation. Proc Natl Acad Sci U S A. 2017;114(50):E10697-E10706. doi:10.1073/pnas.1717327114

Morgan CJ, Pyne-Geithman GJ, Jauch EC, Shukla R, Wagner KR, Clark JF and Zuccarello M (2004). Bilirubin as a cerebrospinal fluid marker of sentinel subarachnoid hemorrhage: a preliminary report in pigs. J Neurosurg 101:1026-1029

Mutlu A, Livanelioglu A, Gunel MK. Reliability of Ashworth and Modified Ashworth scales in children with spastic cerebral palsy. BMC Musculoskelet Disord. 2008;9:44. Published 2008 Apr 10. doi:10.1186/1471-2474-9-44.

Nathwani AC, Tuddenham EG, Rangarajan S, et al. Adenovirus-associated virus vector-mediated gene transfer in hemophilia B. N Engl J Med 2011;365(25):2357-65 doi: 10.1056/NEJMoa1108046

Nathwani AC, Reiss UM, Tuddenham EG, et al. Long-term safety and efficacy of factor IX gene therapy, 2014

Pardridge WM. 1991. Title: Peptide drug delivery to the brain, Chapter: Transnasal and intraventricular delivery of drugs. Raven Press, New York. (ISBN: 0881677930 9780881677935)

Pardridge WM. Drug transport in brain via the cerebrospinal fluid. Fluids Barriers CNS 2011;8(1):7 doi: 10.1186/2045-8118-8-7.

Peterson MR, Cherukuri V, Paulson JN, et al. Normal childhood brain growth and a universal sex and anthropomorphic relationship to cerebrospinal fluid. J Neurosurg Pediatr. 2021;28(4):458-468. Published 2021 Jul 9. doi:10.3171/2021.2.PEDS201006

Samaranch L, Salegio EA, San Sebastian W, Kells AP, Bringas JR, Forsayeth J and Bankiewicz KS (2013) Strong cortical and spinal cord transduction after AAV7 and AAV9 delivery into the cerebrospinal fluid of nonhuman primates. Hum Gene Ther 24:526-32. doi: 10.1089/hum.2013.005

Samaranch L, Salegio EA, San Sebastian W, Kells AP, Foust KD, Bringas JR, Lamarre C, Forsayeth J, Kaspar BK and Bankiewicz KS (2012) Adeno-associated virus serotype 9 transduction in the central nervous system of nonhuman primates. Hum Gene Ther 23:382-9. doi: 10.1089/hum.2011.200

Saraiva J, Nobre RJ and Pereira de Almeida L (2016). Gene therapy for the CNS using AAVs: The impact of systemic delivery by AAV9. J Control Release 241:94-109. doi: 10.1016/j.jconrel.2016.09.011.

Snyder BR, Gray SJ, Quach ET, Huang JW, Leung CH, Samulski RJ, Boulis NM and erici T (2011) Comparison of Adeno-Associated Viral Vector Serotypes for Spinal Cord and Motor Neuron Gene Delivery. Hum Gene Ther. 22(9):1129-1135

Sullivan HG, Miller DJ, Griffith RL, Carter W and Rucker S (1979). Bolus versus steady-state infusion for determination of CSF outflow resistance. Ann. Neurology. 5:228-238

Toh WH, Tan JZ, Zulkefli KL, Houghton FJ, Gleeson PA. Amyloid precursor protein traffics from the Golgi directly to early endosomes in an Arl5b- and AP4-dependent pathway. Traffic. 2017;18(3):159-175. doi:10.1111/tra.12465

Zacharia A, Zimine S, Lovblad KO, Warfield S, Thoeny H, Ozdoba C, Bossi E, Kreis R, Boesch C, Schroth Gand Hüppi PS (2006). Early Assessment of Brain Maturation by MR Imaging Segmentation in Neonates and Premature Infants. American Journal of Neuroradiology. 27:972-977
