# Supplementary material for: AAV gene therapy for hereditary spastic paraplegia type 50: a phase 1 trial in a single patient
Source: Nat Med. 2024 Jun 28;30(7):1882–7. doi: 10.1038/s41591-024-03078-4 (PMC11271397; doi:10.1038/s41591-024-03078-4)
Supplement: Supplementary file 2 — Reporting Summary [file 41591_2024_3078_MOESM2_ESM.pdf]

Corresponding author(s): Dowling

Last updated by author(s): Mar 29, 2024

Reporting Summary

Nature Portfolio wishes to improve the reproducibility of the work that we publish. This form provides structure for consistency and transparency in reporting. For further information on Nature Portfolio policies, see our Editorial Policies and the Editorial Policy Checklist.

Statistics

For all statistical analyses, confirm that the following items are present in the figure legend, table legend, main text, or Methods section.

n/a	Confirmed	
☐	☒	The exact sample size (n) for each experimental group/condition, given as a discrete number and unit of measurement
☐	☒	A statement on whether measurements were taken from distinct samples or whether the same sample was measured repeatedly
☐	☒	The statistical test(s) used AND whether they are one- or two-sided<br>*Only common tests should be described solely by name; describe more complex techniques in the Methods section.*
☒	☐	A description of all covariates tested
☒	☐	A description of any assumptions or corrections, such as tests of normality and adjustment for multiple comparisons
☒	☐	A full description of the statistical parameters including central tendency (e.g. means) or other basic estimates (e.g. regression coefficient) AND variation (e.g. standard deviation) or associated estimates of uncertainty (e.g. confidence intervals)
☐	☒	For null hypothesis testing, the test statistic (e.g. F, t, r) with confidence intervals, effect sizes, degrees of freedom and P value noted<br>*Give P values as exact values whenever suitable.*
☒	☐	For Bayesian analysis, information on the choice of priors and Markov chain Monte Carlo settings
☐	☒	For hierarchical and complex designs, identification of the appropriate level for tests and full reporting of outcomes
☒	☐	Estimates of effect sizes (e.g. Cohen's d, Pearson's r), indicating how they were calculated

Our web collection on statistics for biologists contains articles on many of the points above.

Software and code

Policy information about availability of computer code

Data collection	REDCap 14.1.4
Data analysis	Statistical analysis of ELISpot data was performed using GraphPad Prism.

For manuscripts utilizing custom algorithms or software that are central to the research but not yet described in published literature, software must be made available to editors and reviewers. We strongly encourage code deposition in a community repository (e.g. GitHub). See the Nature Portfolio guidelines for submitting code & software for further information.

Data

Policy information about availability of data

All manuscripts must include a data availability statement. This statement should provide the following information, where applicable:

- Accession codes, unique identifiers, or web links for publicly available datasets
- A description of any restrictions on data availability
- For clinical datasets or third party data, please ensure that the statement adheres to our policy

All data relevant to supporting the findings reported in this study are available within the paper and in the Supplementary Information. Restrictions apply to some information related to the study, which are protected per institutional review board requirements. Sequence and structure of MELPIDA are included as Extended Data Figure 10.
For all data inquiries, please contact Ana Stosic (ana.stosic@sickkids.ca) and/or James Dowling (james.dowling@sickkids.ca). Data or Material Transfer Agreements

may be required and will be assessed at the time of request.

Research involving human participants, their data, or biological material

Policy information about studies with human participants or human data. See also policy information about sex, gender (identity/presentation), and sexual orientation and race, ethnicity and racism.

Reporting on sex and gender	As an n=1 study, sex and gender are not considered as relevant variables and are thus not presented.
Reporting on race, ethnicity, or other socially relevant groupings	As an n=1 study, race and ethnicity are not considered as relevant variables and are thus not presented.
Population characteristics	This is an n=1 study. Patient (male) was 4 years old at the time of dosing.
Recruitment	Details of recruitment are given in the clinical trial protocol, provided as an appendix. The subject was recruited through the SickKids neurology clinic. Bias is difficult to assess as the patient is the only known patient with SPG50 in Canada.
Ethics oversight	Notice to proceed was obtained from Health Canada. Ethics oversight was provided by the regulatory ethics board (REB) at the Hospital for Sick Children.

Note that full information on the approval of the study protocol must also be provided in the manuscript.

Field-specific reporting

Please select the one below that is the best fit for your research. If you are not sure, read the appropriate sections before making your selection.

☒ Life sciences ☐ Behavioural & social sciences ☐ Ecological, evolutionary & environmental sciences

For a reference copy of the document with all sections, see nature.com/documents/nr-reporting-summary-flat.pdf

Life sciences study design

All studies must disclose on these points even when the disclosure is negative.

Sample size	The study was designed to be a single patient (i.e. individualized) phase 1 clinical trial.
Data exclusions	No data points were excluded.
Replication	Certain outcome measures were confounded by subject participation. These are highlighted within the manuscript and in the Supplementary material. Multiple attempts were made to capture all data points. However, due to subject intolerance, some data (specifically related to the spasticity measures) were not able to be obtained.
Randomization	Randomization is not relevant to this study design, as the cohort was a single patient study.
Blinding	This was an open label, single patient, phase 1 study.

Reporting for specific materials, systems and methods

We require information from authors about some types of materials, experimental systems and methods used in many studies. Here, indicate whether each material, system or method listed is relevant to your study. If you are not sure if a list item applies to your research, read the appropriate section before selecting a response.

Materials & experimental systems

n/a	Involved in the study	
☒	☐	Antibodies
☒	☐	Eukaryotic cell lines
☒	☐	Palaeontology and archaeology
☒	☐	Animals and other organisms
☐	☒	Clinical data
☐	☒	Dual use research of concern
☒	☐	Plants

Methods

n/a	Involved in the study	
☒	☐	ChIP-seq
☒	☐	Flow cytometry
☒	☐	MRI-based neuroimaging

Clinical data

Policy information about clinical studies

All manuscripts should comply with the ICMJE guidelines for publication of clinical research and a completed CONSORT checklist must be included with all submissions.

Clinical trial registration	NCT06069687
Study protocol	Study protocol is provided as an appendix.
Data collection	All data was collected at the Hospital for Sick Children. Data collection points (i.e. when labs were drawn and assessment performed) are outlined in the methods section.
Outcomes	Pre-defined primary and secondary outcome measures were identified. These included safety (serum and CSF laboratory studies, nerve conduction studies, brain MRI, and clinical assessment for ad) and efficacy (spasticity scales, bayley scale). Safety studies represented the primary outcome(s). Spasticity measures were the nominated secondary outcomes. The Bayley scale, CGI, and Vineland scale were exploratory efficacy outcome measures.

Dual use research of concern

Policy information about dual use research of concern

Hazards

Could the accidental, deliberate or reckless misuse of agents or technologies generated in the work, or the application of information presented in the manuscript, pose a threat to:

No	Yes	
☒	☐	Public health
☒	☐	National security
☒	☐	Crops and/or livestock
☒	☐	Ecosystems
☒	☐	Any other significant area

Experiments of concern

Does the work involve any of these experiments of concern:

No	Yes	
☒	☐	Demonstrate how to render a vaccine ineffective
☒	☐	Confer resistance to therapeutically useful antibiotics or antiviral agents
☒	☐	Enhance the virulence of a pathogen or render a nonpathogen virulent
☒	☐	Increase transmissibility of a pathogen
☒	☐	Alter the host range of a pathogen
☒	☐	Enable evasion of diagnostic/detection modalities
☒	☐	Enable the weaponization of a biological agent or toxin
☒	☐	Any other potentially harmful combination of experiments and agents
