# Supplementary material for: AAV gene therapy for hereditary spastic paraplegia type 50: a phase 1 trial in a single patient
Source: Nat Med. 2024 Jun 28;30(7):1882–7. doi: 10.1038/s41591-024-03078-4 (PMC11271397; doi:10.1038/s41591-024-03078-4)
Supplement: Supplementary file 3 — MELPIDA certificate of analysis. [file 41591_2024_3078_MOESM3_ESM.pdf]

CERTIFICATE OF ANALYSIS

Name and type of product: MELPIDA - Sterile Suspension of Adeno-Associated Virus serotype AAV9 expressing the therapeutic human AP4M1 (AAV9.AP4M1).

Batch Number: G-GEMINIS-029

Date of manufacture: 6 JULY 2021 (vialing date).

Excipients: Water for Injection, 10mM Phosphate, 137mM NaCl, 2.7 mM KCl, 5% Sorbitol, 0.001% pluronic, pH7.4.

Quality grade: Gene Therapy investigational medicinal product for human use

Primary container: Daikyo CZ COP 2 mL vials and Westar stoppers from WEST Pharma

Expiry date/retest date: Stability studies on going.

Concentration: $1.0x10^{14}$ vg/mL(ITRddPCR method)

Storage conditions: $\leq$-60°C.

Specification no.: SP-GEMINIS.01

Name and address of the manufacturer:

VIRALGEN - Parque Tecnológico de Gipuzkoa - Paseo Mikeletegi 83, 2ª Planta.

CP20009 San Sebastián, SPAIN. Tel: +34 943 477 733.

MIA Reference: 6711E.

SAFETY ASSAYS					
QUALITY ATTRIBUTE	SPECIFICATION	METHOD	PRODUCTION STEP	TEST SITE	RESULT
Sterility	No growth	Filtration<br>EP 2.6.1; USP<71><br>LTMI/009	Drug Product	Biolab	No growth
Endotoxin EU/mL	≤0.20	Kinetic Chromogenic<br>E.P 2.6.14; USP<85><br>PNT-CC-015	Drug Product	Viralgen	<0.05
Mycoplasma[1]	No detected	PCR end Point<br>EP 2.6.7<br>PNT-CC-001	Transfection Pool	Viralgen	No detected
Mycobacteria	No detected	Quantitative PCR<br>EP 2.6.16	Transfection Pool	SGS-Vitrology	No detected
Adventitious Virus	No cytopathic effect, no haemagglutination, No haemadsorption	In vitro cell assay 3 cell lines<br>EP 2.6.16; USP <1050>	Transfection Pool	SGS-Vitrology	No cytopathic effect, No haemagglutination, No haemadsorption
Bioburden (at transfection pool)	< 10CFU/mL	Direct seeding<br>EP 2.6.12; USP<61><br>PNT-CC-018	Transfection Pool	Viralgen	< 10CFU/mL
Bioburden (prior sterile filtration)	≤ 1CFU/10 mL (TAMC)	Filtration<br>EP 2.6.12; USP<61><br>PNT-CC-026	Drug Substance	Viralgen	< 1CFU/10 mL
Appearance	Colorless, clear to slightly opalescent, free of visible particles	Visual Inspection<br>EP 2.9.20; USP<790><br>PNT-CC-017	Drug Product	Viralgen	Colorless, clear to slightly opalescent, free of visible particles
Container Clossure Integrity [2]	No dye penetration	Dye Penetration<br>EP 3.2.9; USP<1207><br>FTN-MTH309.V1	Filling (done with formulation buffer)	Eurofins	No dye penetration
Subvisible particles[3]	<6000 particles ≥10µm<br><600 particles ≥25µm	Method 1 Light Obscuration Particle Count Test. Test 1B<br>EP 2.9.19; USP<788>	Drug Product	Echevarne	121 particles ≥ 10 µm<br>1 particles ≥ 25 µm
Replication competent AAV	Report Result (based on ITRddPCR titer)	Infection of permissive cell line / rep qPCR	Drug Substance	Genosafe	No detected<br><10rcAAV in $1x10^{11}$ vg

STRENGHT ASSAYS					
QUALITY ATTRIBUTE	SPECIFICATION	METHOD	PRODUCTION STEP	TEST SITE	RESULT
Vector genome titer (vg/mL)	Report Results	ITRqPCR<br>EP 2.6.21; USP<1127><br>PNT-CC-005	Drug Substance	Viralgen	$3.04x10^{14}$
Vector genome titer (vg/mL)	$5x10^{13}$-$2x10^{14}$ (targeting $1x10^{14}$ vg/ml)	ITRddPCR<br>EP 2.6.21; USP<1127><br>PNT-CC-049	Drug Substance	Viralgen	$1.0 x10^{14}$
Vector genome titer(vg/mL)	Report Results	ITRqPCR<br>EP 2.6.21; USP<1127><br>PNT-CC-005	Drug Product	Viralgen	$3.78 x10^{14}$
Vector genome titer (vg/mL)	$5x10^{13}$-$2x10^{14}$ (targeting $1x10^{14}$ vg/ml)	ITRddPCR<br>EP 2.6.21; USP<1127><br>PNT-CC-049	Drug Product	Viralgen	$1.0 x10^{14}$
Infectious particles titer (TCID50/mL)	Report Result (TCID50/mL)	TCID50<br>ITR qPCR target amplification<br>PNT-CC-004	Drug Product	Viralgen	$2.42x10^{10}$

PURITY ASSAYS					
QUALITY ATTRIBUTE	SPECIFICATION	METHOD	PRODUCTION STEP	TEST SITE	RESULT
General purity	Detection of VP1, VP2 and VP3	SDS-PAGE/Silver Stain<br>EP 2.2.31;<br>USP<1056><br>PNT-CC-012	Drug Substance	Viralgen	Detection of VP1,VP2 and VP3:faint bands between 150Kda and 250 Kda
Residual Host Cell Protein (ng/mL)	Report Result	ELISA<br>EP 2.6.34;<br>USP<1132><br>SP-B.4401	Drug Substance	SGS-Vitrology	53.86
Residual Host Cell DNA-18S (pg/mL)	Report Result	Quantitative PCR of 18S DNA. 2 Amplicons: 123 & 254 base pairs.<br>EP 2.6.35;<br>USP<1130><br>PNT-CC-033	Drug Substance	Viralgen	**123 bp:**<br>$1.11x10^7$ (-DNase)<br>$1.06x10^7$ (+(DNAse)<br>**254 bp:**<br>$8.11x10^6$(-DNase)<br>$7.93x10^6$(+DNAse)
Residual Host Cell DNA E1A (pg/mL)	Report Result	Quantitative PCR of E1A<br>EP 2.6.35;<br>USP<1130><br>SP-M.8303	Drug Substance	SGS-Virology	$6.6 x10^6$ (-DNAse)
Residual plasmid DNA (copies/mL)	Report Result	Quantitative PCR of Kanamycin resistance gene<br>EP 2.6.21;<br>USP<1127><br>PNT-CC-014	Drug Substance	Viralgen	$1.85x10^{12}$ (-DNase)<br>$1.06 x10^{12}$ (+DNase)
Full/Empty particles ratio	>50% full	CryoTEM<br>V6725	Drug Substance	Vironova	79% filled<br>11% empty<br>10% uncertain
Vector Aggregation	Report Result	nsTEM<br>V7915	Drug Substance	Vironova	84% individual particles <40 nm<br>16% aggregates >40 nm

PURITY ASSAYS					
**QUALITY ATTRIBUTE**	**SPECIFICATION**	**METHOD**	**PRODUCTION STEP**	**TEST SITE**	**RESULT**
Residual chemicals (cells lysis reagent) (ppm)	Report Result	Refer to DMF	Drug Substance	Dochuchem	<LOD (1.01)
Residual chemicals (clarifying reagent) (ppm)	Report Result	Refer to DMF	Drug Substance	Dochuchem	<LOD (0.10)
Residual chemicals (transfection reagent) (ppm)	Report Result	Refer to DMF	Drug Substance	Dochuchem	<25.41<br>>LOD (12.71)
Residual chemicals (antifoam) (ppm)	Report Result	Refer to DMF	Drug Substance	Dochuchem	<LOD (5)
Residual chemicals (Iodixanol) (ppm)	Report Result	HPLC	Drug Substance	Dochuchem	<0.22<br>>LOD (0.11)
Residual immunoaffinity ligand (ng/mL)	Report Result	ELISA<br>EP 2.7.1; USP<1103><br>PNT-CC-037	Drug Substance	Viralgen	212.15

IDENTITY ASSAYS					
**QUALITY ATTRIBUTE**	**SPECIFICATION**	**METHOD**	**PRODUCTION STEP**	**TEST SITE**	**RESULT**
Genomic identity	100% conform to sequence of reference	Sanger Sequencing PNT145	Drug Substance	Outsourced	100% conform to sequence of reference
Protein identity	Detection of VP1, VP2 and VP3	SDS-PAGE/Western Blot<br>PNT-CC-003	Drug Substance	Viralgen	Detection of VP1, VP2 and VP3

GENERAL QUALITY ASSAYS					
QUALITY ATTRIBUTE	SPECIFICATION	METHOD	PRODUCTION STEP	TEST SITE	RESULT
Packaging	Clear and readable labels. Container without breakage or leakage	Visual Inspection	Drug Product	Viralgen	Clear and readable labels. Container without breakage or leakage
Osmolality mOsm/Kg	537-637	Freezing Point Depression EP 2.2.35; USP <785> PNT-CC-016	Drug Product	Viralgen	572
pH	7.1-7.7	Potentiometry EP 2.2.3; USP <791> PNT-CC-044	Drug Product	Viralgen	7.3
Extractable Volume[4]	No less than nominal volume	EP 2.9.17; USP<697> PNT-CC-024	Filling (done with formulation buffer)	Viralgen	No less than nominal volume
Particle size distribution	Report Result	Dynamic Light Scattering PNT-CC-053	Drug Product	Viralgen	100% of particles with size 23.89-24.45 nm

[1] Mycoplasma test performed by the validated nucleic amplification (NAT) alternative method as referred in USP <63> - (introduction section).

[2] CCIT test performed on vials containing formulation buffer.

[3] Subvisible particles tested according to EP 2.9.19, with 5 vials of Drug Product (5 mL) with a 1/5 dilution

[4] Extractable volume performed on vials containing formulation buffer.

[redacted]
